# Structural and functional dissection of differentially expressed tomato WRKY transcripts in host defense response against the vascular wilt pathogen (*Fusarium oxysporum* f. sp. *lycopersici*)

**DOI:** 10.1371/journal.pone.0193922

**Published:** 2018-04-30

**Authors:** Mohd Aamir, Vinay Kumar Singh, Manish Kumar Dubey, Sarvesh Pratap Kashyap, Andleeb Zehra, Ram Sanmukh Upadhyay, Surendra Singh

**Affiliations:** 1 Laboratory of Mycopathology and Microbial Technology, Centre of Advanced Study in Botany, Institute of Science, Banaras Hindu University, Varanasi, Uttar Pradesh, India; 2 Centre for Bioinformatics, School of Biotechnology, Institute of Science, Banaras Hindu University, Varanasi, Uttar Pradesh, India; 3 Division of Crop Improvement and Biotechnology, Indian Institute of Vegetable Research, Indian Council of Agricultural Research (ICAR), Varanasi, Uttar Pradesh, India; Bhabha Atomic Research Centre, INDIA

## Abstract

The WRKY transcription factors have indispensable role in plant growth, development and defense responses. The differential expression of *WRKY* genes following the stress conditions has been well demonstrated. We investigated the temporal and tissue-specific (root and leaf tissues) differential expression of plant defense-related *WRKY* genes, following the infection of *Fusarium oxysporum* f. sp. *lycopersici* (*Fol*) in tomato. The genome-wide computational analysis revealed that during the *Fol* infection in tomato, 16 different members of *WRKY* gene superfamily were found to be involved, of which only three WRKYs (*SolyWRKY4*, *SolyWRKY33*, and *SolyWRKY37*) were shown to have clear-cut differential gene expression. The quantitative real time PCR (qRT-PCR) studies revealed different gene expression profile changes in tomato root and leaf tissues. In root tissues, infected with *Fol*, an increased expression for *SolyWRKY33* (2.76 fold) followed by *SolyWRKY37* (1.93 fold) gene was found at 24 hrs which further increased at 48 hrs (5.0 fold). In contrast, the leaf tissues, the expression was more pronounced at an earlier stage of infection (24 hrs). However, in both cases, we found repression of *SolyWRKY4* gene, which further decreased at an increased time interval. The biochemical defense programming against *Fol* pathogenesis was characterized by the highest accumulation of H_2_O_2_ (at 48 hrs) and enhanced lignification. The functional diversity across the characterized WRKYs was explored through motif scanning using MEME suite, and the WRKYs specific gene regulation was assessed through the DNA protein docking studies The functional WRKY domain modeled had β sheets like topology with coil and turns. The DNA-protein interaction results revealed the importance of core residues (Tyr, Arg, and Lys) in a feasible WRKY-W-box DNA interaction. The protein interaction network analysis revealed that the SolyWRKY33 could interact with other proteins, such as mitogen-activated protein kinase 5 (MAPK), sigma factor binding protein1 (SIB1) and with other WRKY members including WRKY70, WRKY1, and WRKY40, to respond various biotic and abiotic stresses. The STRING results were further validated through Predicted Tomato Interactome Resource (PTIR) database. The CELLO2GO web server revealed the functional gene ontology annotation and protein subcellular localization, which predicted that SolyWRKY33 is involved in amelioration of biological stress (39.3%) and other metabolic processes (39.3%). The protein (SolyWRKY33) most probably located inside the nucleus (91.3%) with having transcription factor binding activity. We conclude that the defense response following the *Fol* challenge was accompanied by differential expression of the *SolyWRKY4*(↓), *SolyWRKY33*(↑) and *SolyWRKY37*(↑) transcripts. The biochemical changes are occupied by elicitation of H_2_O_2_ generation and accumulation and enhanced lignified tissues.

## Introduction

Tomato (*Lycopersicon esculentum* Mill.) is one of the most widespread vegetable crop grown across the globe. However, the growth and economic productivity of tomato are well constrained by various biotic and abiotic stress conditions [1, 2]. The pathogenic fungus *Fusarium oxysporum* f. sp. *lycopersici* (*Fol*) is one of the major cause of economic losses covered in tomato production [3]. The fungus is a soil-borne pathogen, which infects tomato plant through roots, and develops vascular wilt disease [4]. The incidence of Fusarium wilt disease in tomato is very high (25–55%), and has been reported from various regions of India [5–7]. The possible economic losses in tomatoes due to *Fol* pathogenesis may rise up to 80% under the favorable weather conditions [8]. It has been reported that the mycotoxins secreted by the fungus have a phytotoxic effect, causes pathogenicity, and play a crucial role in vascular wilt disease development [9]. The exposure of toxic metabolites of the fungus may have moderate toxicity to animal cells, and develop opportunistic infections in humans [10].

The plant defense mechanism and resistance against pathogen challenged conditions are well orchestrated by extensive transcriptional reprogramming in their genome, which occurs both in the systemic tissues as well as at the infection sites, in a multi dynamic and temporal manner. These changes are manifested and occupied by the changes in the expression of genes [11]. It has been suggested that during the exposure of stress, plant transcription factors, that belongs to multiple families, participate and play a critical role in stress mitigation or other adjustment mechanisms, through modulating the gene expression patterns [12]. Transcription factors (TFs) could be defined as proteins, that regulates the transcriptional changes, following the stress conditions through their interaction with *cis -*elements present in the promoter region of stress-responsive genes [13] and therefore, regulating the expression of stress-inducible genes [13,14] required for activation of host (plant) defense mechanism [15]. The gene expression profiling and the mechanism of plant defense response following the infection of *F*.*oxysporum* f. sp. *ciceri* (*Foc*), causing vascular wilt disease, in chickpea is well studied [16]. The increased expressions of defense-related genes in tomato plant under the *Fol* challenged conditions have been well documented [17]. It has been suggested through several studies that during the pathogenic attack, plants respond well through several defense-related mechanisms such as activation of defense signaling pathways, reactive oxygen species (ROS) generation, accumulation of H_2_O_2,_ reinforcement of cell wall through deposition of lignin and suberin, at the infection sites, activation and expression of plant pathogenesis-related (PR) proteins [18] which reflects, the involvement of systemic acquired resistance (SAR), that is mediated by salicylic acid (SA) hormonal signaling [19]. The influence of plant cell wall modification following the pathogenic challenge is well reported through several studies [20, 21]. It has been reported that during the pathogen (fungal) challenged conditions plant accumulate soluble and cell wall-bound (CWB) phenolic compounds which impart resistance against pathogen attack as a part of antimicrobial defense arsenals [22]. The positive correlation between the amount of lignin deposition and pathogen resistance is well evidenced [23]. The plant resistance is driven by oxidative burst, and enhanced lignification following the infection of *Fusarium oxysporum* f. sp. *cubense* (*Foc*) was observed in banana plants [24]. Overall, all these changes contribute towards plant defense response [25]. It has been reported that, in tomato *WRKY* genes, regulate the various developmental processes, and also play a crucial role in defense signaling generated against multiple stresses, and have distinct spatial and temporal gene expression pattern [26].

The *WRKY* gene superfamily represents a large group of transcription factors, with characteristic WRKY domain, and has widespread distribution in plants [27]. The most convincing feature of the WRKY domain, is the presence of highly conserved heptapeptide sequence “WRKYGQK” locating at their N-terminal end, and the presence of zinc finger motifs C_2_H_2_ (C–X_4_−_5_–C–X_22_−_23_–H–X_1_–H) or C_2_HC (C–X_7_–C–X_23_–H–X_1_–C) at their C-terminus [28, 29]. The number of the zinc finger motifs also vary and usually, the presence of one or two motifs, were reported to be involved in WRKY specific W-box (C/T) TGAC(C/T) DNA binding [30]. These WRKY TFs may work as the repressor as well as activators since they get involved in the regulation of both repression and de-repression of the crucial physiological and developmental processes in plants [31]. The defense signaling network encompasses many defense-associated genes, which along with WRKY member or other signaling proteins modulates the overall expression of stress-responsive genes, through auto-regulation, cross-regulation or protein-protein interactions, to fine tune the defense response against multiple stresses [31, 32–34]. The modulation of a transcriptional regulatory network with signaling component and other interacting proteins is a critical process involved in the activation or repression of defense-related signal transduction pathways [35]. The extensive cross-communication between the different hormonal signaling pathways is always required to fine-tune the transcriptional programme, necessary for determining the resistance against invaders and trade-off with plant development [36].

The direct role of WRKY proteins in plant defense response has been well demonstrated in several studies. For example, *Arabidopsis* mutants for *WRKY70* gene have been reported to be more susceptible to both necrotrophic as well as biotrophic pathogens such as bacterial pathogen *Erwinia carotovora* and other fungal pathogens including *Botrytis cinerea* and *Erysiphe cichoracearum* [37–39]. It has been reported that some WRKY members, including WRKY4, WRKY33, WRKY40, WRKY60 and a redundant WRKY18, play a positive role in providing resistance to plants against necrotrophic pathogens [40, 41], and their induction in response to biotrophic, hemibiotrophic and necrotrophic fungi is well studied [42]. Moreover, *Arabidopsis wrky33* mutant has been demonstrated to have a higher susceptibility to necrotrophic pathogens but respond normally against biotrophic pathogens [40]. The regulation of the hormonal cross-talk between the defense signaling pathways, against the diverse types of microbial pathogens, occurs at the transcriptional level [43]. For example, some WRKYs may regulate the cross-talk by activating the expression of JA/ET-regulated genes, but repressing the SA regulated genes [43]. The fine-tuning of gene regulation for gene expression in a direct and antagonistic manner requires WRKY TFs that may act as transcriptional activators or repressors in a gene-specific manner [43]. The WRKY transcription factors regulate the crosstalk by activating expression of genes associated with JA/ET-mediated signaling pathways, including some encoding transcriptional repressor that suppresses SA regulated gene expression [43].

The extensive perusal of literature revealed that till date, no information is available regarding the expression of the plant defense-related *WRKY* genes, following the infection of vascular wilt pathogen, *Fol* in tomato. Therefore, the aim of the present study was to evaluate the tissue-specific expression profile of tomato defense-related *WRKY* genes at the different time interval, under the *Fol* challenged conditions. Our study aimed to obtain novel insights into genome wide microarray analysis of differentially expressed *WRKY* genes in tomato under *Fol* challenged conditions, and to study their expression profiles, symptom characteristics at the biochemical level and their interaction network, The evolutionarily aspects of characterized WRKYs in tomato and other related members were investigated. Further, the structural and functional attributes of reported WRKYs were also explored to unravel the functional defense mechanism of WRKY specific gene regulation (WRKY-W-box DNA interaction) occurring at the molecular level using computational approaches.

## Materials and methods

### A. *In-vitro* experiments

#### Fungal isolate, plant and growth conditions

The pathogenic culture of the wilt pathogen Fusarium oxysporum f. sp. lycopersici (Fol) was obtained from the Department of Mycology and Plant Pathology, Institute of Agricultural Sciences, Banaras Hindu University (BHU) and was further subcultured and incubated at 25 ± 2°C. The PDA agar slants were stored for further studies. The tomato seeds (S-22 variety) susceptible (83.67%) to Fol pathogen [44] were surface sterilized in 70% ethanol followed by treatment with 0.6% mercuric chloride, for 2 min and then were washed three times with the autoclaved distilled water. The sterilized seeds were further sowed in the fresh plastic pots (having 08 cm diameter) containing the sterilized soil mixed with vermiculate (2:1). A total of five replicates for each treatment were prepared and maintained at 16 hrs light/8hrs dark in a greenhouse at 28–29° C following the protocol [45].

#### *Quantitative real time PCR* (*qRT-PCR) analysis*

Fungal Inoculum Preparation

To prepare the fungal inoculum, the spore suspension from seven days old pathogenic culture of Fusarium *oxysporum* f. sp. *lycopersici* was employed. The spore suspension was prepared by following the protocol [46]. In brief, the suspension was prepared by adding the sterile distilled water to the Petri-dishes, with gentle removal of spores by using a glass spreader and filtering the heterogeneous suspension through four layered muslin cloth to prevent the fungal mycelium. Further, the filtered suspension was adjusted with sterile distilled water to obtain a minimum density of 2 x 10^5^ to 2 x 10^6^ spores mL^-1^ and quantified with the help of hemocytometer.

Treatment of Plant material

The tomato plants were allowed to grow and when they attained a height of 15 cm (5–6 weeks old), were treated with the spore suspension of Fol following the methods as suggested by [45]. The root and leaf tissue samples from both control (untreated) and Fol challenged samples were collected at different time interval 0 hrs (control), 24 hrs and 48 hrs for gene expression (quantitative real time PCR) studies. The leaf tissues were further used for biochemical assessment of H_2_O_2_ produced under Fol induced biotic stress at 48 hrs.

Gene Expression Analysis

The relative expression levels of distinctly upregulated defense-related *WRKY* genes in different tissues (root and leaf) were evaluated at the different time intervals. The total RNA was extracted and isolated from the tomato leaves by using TRI reagent (Ambion) along with RNAase-free DNAase treatment (Qiagen) to remove the genomic DNA contamination. The 1^st^ strand cDNA was synthesized from 1.0 μg of total RNA by employing the first strand cDNA synthesis kit (Bio-Rad) in the 20μL reaction volume and by following the manufacturer’s instructions (Bio-Rad). RT-PCR was carried out using 1^st^ strand cDNA as template (2 μl) in 50 μl reaction volume containing 36 μl of H_2_O, 5 μl of 10X PCR buffer, 3 μl of 25 mM MgCl_2_, 1 μl of 10 mM dNTP mix, 1μl of each 10 mM sense and anti-sense primers and 1 μl of Taq DNA polymerase (Fermentas Life Sciences) [47]. The specific primer pairs for the quantitative real-time RT-PCR (qPCR) were designed by using the web-based primer designing tool Primer 3 http://primer3.ut.ee/ [48] (Table 1) and the obtained primer sequences were further checked and validated through the online tool Primer Blast at https://www.ncbi.nlm.nih.gov/tools/primer-blast/ [49]. The PCR programming was performed at 94°C (5 min) for 1 cycle followed by 35 cycles at 94°C (30 sec), annealing at 50–55°C depending upon melting temperature (Tm) of primers for 30 sec and extension 72°C (40 sec), and finally 1 cycle at 72°C (10 min). The PCR fragments integrity was further checked on a 1.2% agarose gels for their electrophoretic separation. The qPCR was performed in triplicates using SYBR Green fluorescence dye (Qiagen, USA) and were analyzed by using iQ-SYBR Green Supermix (Bio-Rad, CA, USA) on iQ5 thermocycler (Bio-Rad, CA, USA) with iQ5 Optical System Software version 2.0 (Bio-Rad, CA, USA) following the protocols as mentioned. The difference between the C_t_ values of the target gene and the housekeeping gene actin (that act as the constitutive control) were calculated (ΔC_t_ value) for the respective templates to normalize the expression values of a targeted gene under study. The ΔC_t_ value was calculated for finding the fold change (FC) in the relative gene expression level compared to control and was calculated as follows: ΔC_t_ = C_t_ (target gene) − C_t_ (constitutive control gene). The relative transcript levels were determined following the formula 1000 × 2 – ΔC_t_ [50]. The differential expression of the replicated count data obtained for the healthy control and *Fol* challenged tissues were further used for clustering the replicated count data and relative fold change expression values for Bioconductor R [51] analysis to generate Heatmap for expression profile changes in tissue-specific and temporal manner.

**Table 1:** List of tomato *WRKY* gene specific primers both forward(F) and reverse (R) used in quantitative PCR along with housekeeping gene actin used as constitutive control.

S.No	Gene Name	Primer Sequence(5–3’)	T_m_	GC%
1	SolyWRKY4 Forward Primer	CGTTGCACATACCCTGGATG	58.98	55.00
2	SolyWRKY4 Reverse Primer	GGCCTCCAAGTTGCAATCTC	59.19	55.00
3	SolyWRKY33 Forward Primer	CCACCTCCTTCACTTCCATT	57.11	50.00
4	SolyWRKY33 Reverse Primer	GATGGAAAACTCCCAGTCGT	57.53	50.00
5	SolyWRKY37 Forward Primer	CAGATGCAGCAGTTCAAAGG	57.37	50.00
6	SolyWRKY37 Reverse Primer	CTTCGAGGGACACATGTTGA	57.54	50.00
7	Actin (Constitutive control) Forward primer	GAAATAGCATAAGATGGCAGACG	58.9	43.5%
8	Actin Reverse	ATACCCACCATCACACCAGTAT	58.4	45.5%

#### Histopathological tests

Preparation of Culture extract

The culture filtrate (crude extract having toxic metabolites) of *Fol* was used for the assessment of disease symptoms (necrotic lesions) and effect of *Fol* toxins on host tissues. For the preparation of culture filtrate, five fungal discs each of 5 mm diameter were retrieved from fresh culture plate and were inoculated in 250 mL conical flask containing PDB broth medium. The flasks were kept on a rotatory shaker for the period of 20 days maintained at 28°C with 120 rpm. At harvesting, the mycelium mat was removed from the filtrate and further filtered with using Whatman 03 mm filter paper. The broth cultures were then filter sterilized through a millipore filter (pore diameter of 0.2 μm) [52]. The culture filtrate toxic metabolites from were further used for histopathological tests.

Treatment of Plant material

For histopathological tests, the young and fresh leaf tissues were slightly punctured with sandpaper and were uniformly sprayed with 0.5 mL crude culture filtrate (toxic metabolites) derived from 20 days old *Fol* culture grown in 250 mL PDB broth (as mentioned above) on the leaves attached with plants following the protocol [52]. A control leaf (attached) was also maintained along with the treated samples and was sprayed with sterile distilled water Simultaneously, some other leaves that were detached from tomato plants washed with water, and given the same treatment, then the leaves were kept in a tray (on blotting paper rinsed with water to provide moist conditions) at room temperature under sterile conditions [53]. The leaf tissue samples (both treated and control) were regularly monitored for next 48 hrs and were further used for histochemical analysis to observe the H_2_O_2_ accumulated (DAB staining) and assessment of cell death (Evans blue staining).

#### Biochemical assessment of plant defense response

DAB Staining for Detection of H_2_O_2_ Accumulation

Histochemical staining of the leaves was done to visualize the accumulated H_2_O_2_ using 3, 3-diaminobenzidine (DAB) following the given protocol [54]. After 48 hrs the leaves attached with plant were cut with a sterilized blade above the base of petiole and were dispensed in a beaker containing 50 mL of DAB-HCl (1mg/mL) (pH 5.6). The detached and control leaf samples were dispensed separately. Further, all the samples were incubated in a humid growth chamber for 12 hrs (overnight) in a dark place [53]. After DAB uptake, the leaves were cleared in 96% boiling ethanol and examined under the light microscope. The accumulated H_2_O_2_ is visualized as a reddish-brown coloration.

H_2_O_2_ Quantification

The quantification of the accumulated H_2_O_2_ was done in the leaf tissues that were collected at 0, 24, 48, 72 and 96 hrs from both control and pathogen challenged tomato plants. The leaf tissue sample from each treatment (0.1g) was crushed under 4°C in an ice bath having 2.0 mL of 0.1% (w/v) trichloroacetic acid (TCA). The treated samples were then centrifuged for 10 min at 12,000 × g. The recovered supernatants (0.5 mL) was dissolved with 01 mL of 1M potassium iodide (KI) solution in 10 mM potassium phosphate buffer at pH 7.0 and were further kept at room temperature for 5 min. The spectrophotometric measurement of the oxidized product was done at 390 nm [55]. The amount of H_2_O_2_ accumulated in the control/treated samples were measured by the standard curve correlation obtained through known concentrations of H_2_O_2_ and were expressed as nmol H_2_O_2_ g^−1^ fresh weight.

Evans Blue Staining to Detect Cell Death

The histochemical analysis using Evans blue staining was made to assess the extent of the cell death. The leaves from both control and *Fol* treated samples were boiled for 60 sec in a 50 mL freshly prepared solution containing phenol (99.5%), lactic acid (85% w/w), glycerol (99.5% pure) and distilled water (1:1:1:1) dissolved with 20 mg/mL Evans blue. The treated tissues after boiling were left for overnight in 10 mL solution of chloral hydrate (2.5g/mL) made of water. The leaf tissues were then transferred on a clean glass slide for observation under the microscope. The cell death was measured based on the intake of coloration (dark blue) by dead tissues compared to unstained control samples.

Histochemical Staining for Assessment of Lignification

The assessment of plant defense response in terms of lignified tissues was measured following the method as suggested [56]. The hand sectioned transverse sections (TSs) of tomato stem from the second node was mounted in 1% phloroglucinol solution prepared with the 95% ethanol. The mounted samples were washed with 0.1 mL concentrated HCl solution and was placed then on a clean glass slide and covered with coverslip [56]. The stained samples were visualized under a compound light microscope (Nikon, Japan). The development of pink color showed the tissues having lignins.

#### Statistical analysis

All the statistical analysis and calculation were performed using SPSS version 1.6. The experiment performed in this study was done in triplicates and the experiment was repeated twice using a completely randomized design. The statistical data were expressed as the mean of three independent replications ± standard deviation and were interpreted through one-way analysis of variance (ANOVA) by using the average data of one experiment, while the mean separations were compared with Duncan’s multiple range test at the P ≤ 0.05 significance level.

### B. *In-silico* experiments

#### Data retrieval and gene expression analysis

For identification of the differentially expressed transcripts under the *Fol* challenged conditions the Gene Expression Omnibus (GEO) https://www.ncbi.nlm.nih.gov/gds/ [57, 58]. (GEO accession: GSE52336) was used [17]. Four samples were taken for GEO2R analysis from defined groups, two uninoculated control samples (GSM1263304_rep1 and GSM1263305_rep2) and two *Fol* inoculated and challenged plants (GSM1263308_rep1and 12623309_rep2). The GEO2R analysis was performed to find out the value distribution and profile graph of the expressed genes and the gene information based on default parameter. The differentially expressed gene data were collected in tabulated format (S1 Table) and a separate table was made to sort out the transcripts whose expression were found to be differentially expressed (upregulated in treated) (S2 Table) and downregulated in the control samples (S3 Table) following the pathogen challenged conditions. The only WRKY transcripts that were found to be upregulated in the treated samples were collected by using their EST accession number. Further NCBI Blastx (search protein databases using a translated nucleotide query) was performed to compare the transcripts with the available protein sequences in the tomato database (*Solanum lycopersicum*; taxid: 4081). The EST transcripts having WRKY domain were used for BiGGEsTS (Blustering Gene Expression in Time Series) analysis http://kdbio.inescid.pt/software/biggests/ [59]. The BiGGEsT results revealed the differentially expressed (upregulated and downregulated) WRKY transcripts in both *Fol* inoculated and control samples. The tabulated gene expression datasets generated for *Fol* inoculated and *Fol* un-inoculated were further analyzed and validated through the ClustVis http://biit.cs.ut.ee/clustvis/ [60]. Based on the principal component analysis the heatmap plot for differentially expressed transcripts was generated.

#### Multiple sequence alignment and phylogenetic analysis

Genome-wide in silico microarray data analysis revealed that during *Fol* challenged conditions out of total 16 total differentially expressed WRKYs only three WRKY transcripts (*SolyWRKY4*, *SolyWRKY33*, and SolyWRKY37) were found to be distinctly upregulated. Of these three upregulated WRKY members only two members *SolyWRKY33* and *SolyWRKY37* were used for further studies. The functional characterization of the *SolyWRKY4* (XP_004235494.1) has been already reported in our previous publication [61].The Blast-p program research in the NCBI protein database (http://blast.ncbi.nlm.nih.gov/blast.cgi [62] was used to find sequential homologs for both SolyWRKY33 and SolyWRKY37. Blast annotations were filtered using subject to query coverage of >96% and with sequence identity > 62%. The BioEdit tool was further employed for alignment correction [63]. The Poisson correction was used for data analysis, and alignment gaps were removed. The maximum parsimony tree was used for the construction of phylogenetic tree with 1000 bootstrapping values using Molecular Evolutionary Genetics Analysis (MEGA) version 6.0 [64]. Multiple sequence alignment of the protein sequences representing the WRKY domain region for all the sequential homologs of the SolyWRKY33 and SolyWRKY37 was done to find out the percent consensus and conserved residues along the length of the entire protein in different members using CLC bio workbench http://www.clcbio.com. The functional domain region observed in each WRKYs were scanned and explored using ExPASy-Prosite (http://prosite.expasy.org/scanprosite/) [65]. The sequences were analyzed against the InterPro protein signature databases using Interproscan 5.0 web server (http://www.ebi.ac.uk/Tools/pfa/iprscan/) [66] to find out the potential function of proteins. The functionally conserved motifs in both SolyWRKY33 and SolyWRKY37 proteins were investigated using MEME (Multiple Expectation Maximization for Motif Elicitation (http://meme-suite.org/tools/meme) tool [67]. The number of potential motifs was set up to 10 with having an optimum motif width ranging between 10 and 30 residues, with zero or one occurrence per sequence. The characterized WRKYs were further analyzed for providing a comparative evaluation to identify the similarities and differences across the WRKY members having homologous and/or orthologous relationship with the SolyWRKY33 and SolyWRKY37 using the circos visualization http://circos.ca/ [68]. The circos results were determined based on the percentage identity matrices obtained through phylogenetic clustering using Clustal W at 0 percent cut-off filter values.

#### *Insilico* assessment of the structural and functional attributes of the upregulated WRKYs (WRKY33 and WRKY37) in tomato

Computational Modelling of Functional WRKY domain

The protein sequences of the identified genes were taken for homology modeling and DNA protein docking studies. The template proteins for homology modeling of the domain structure of SolyWRKY33 and SolyWRKY37 were searched using Blast-p program of the Protein Data Bank (www.rcsb.org/pdb/) [69] with available proteins having greater than 90% similarity. Structures relevant to C-terminal domain of AtWRKY4 (PDB ID: 1WJ2) and AtWRKY1 (PDB ID: 2AYD) protein molecules along with the structure of the C-terminal WRKY domain of AtWRKY4 complexed with W-box DNA (PDB ID: 2LEX) were used as templates for 3D structure prediction of the domain region lying in the SolyWRKY33 and SolyWRKY37 using MODELER module of Discovery studio 3.0 [70]. The functional N-terminal WRKY domain region and the C-terminal domain (CTD) from SolyWRKY33 and the functional WRKY domain for SolyWRKY37 were modeled using DS Modeller. The CTDof the modeled SolyWRKY33 was made to aligned over different templates (1WJ2, 2AYD and 2LEX) for finding the topological details using Pymol [71] and the visualization module of the DS Modeller [70] to find out the structural similarities and differences (deviations) and were measured in terms of RMSD calculations. The qualitative assessment for a structurally stable and reliable WRKY domain model was evaluated based on several parameters including the percentage residues lying in favoured, unfavoured or disallowed regions, the number of glycine and proline residues and orientation of their backbone dihedral angles including the phi (*φ*) and psi (*ψ*) angles with using the PROCHECK module of the PDBsum server http://www.ebi.ac.uk/pdbsum/ [72]. Furthermore, qualitative assessment measured in terms of stability and reliability of the predicted models was evaluated through the RAMPAGE server http://mordred.bioc.cam.ac.uk/rapper/rampage.php [73].

*In-silico* cis-Acting Regulatory DNA Element Analysis

The *cis-*acting regulatory element involved in the pathogen-induced defense signaling that constitutes the promoter region of the *WRKY* gene was searched from Plant *cis*-acting regulatory DNA elements (PLACE) database http://www.dna.affrc.go.jp/PLACE/ [74]. The full-length gene prediction was done for finding the promoter region of *WRKY* gene. The protein sequences were scanned using tBlastn programme, and were explored under the whole genome shotgun contigs(wgs) database by selecting organism as *Solanum lycopersicum* (taxid: 4081). The Blast results revealed the homologous sequences (BABP01013778.1: having the sequence identity 84% and the query coverages 96%) and were retrieved into the positive frame through the reverse complementation. The FGENESH programme (http://www.softberry.com/) was used to find out coding sequences, transcriptional start, and end sites.

DNA-Protein Interaction

The molecular docking analysis of WRKY domain from SolyWRKY33 and SolyWRKY37 proteins with W-Box DNA was carried by Hex 8.0 molecular docking software [75]. Hex is an interactive protein docking and molecular superposition program. For effective docking calculation the default parameters were set (Hex server module) with the correlation type:Shape + Electro + DARS; FFT Mode– 3D fast lite; Grid Dimension– 0.6; Receptor range– 180; Ligand Range– 180; Twist range– 360; Distance Range– 40. The DARS (Decoys As the Reference State) potential type correlation provides strong docking results [76] substantially better than the ones by the competing potentials, and must be employed with other energy terms (e.g., van der Waals and electrostatics) [76]. Further, the docked complexes were analyzed using visualization module of DS Studio 3.0 for further interaction studies.

Protein-Protein Interaction

The function of the characterized WRKYs were analyzed through protein-protein interaction network using STRINGS sever (Search Tool for the Retrieval of Interacting Genes/Proteins database version 10.0) (http://string-db.org/) [77]. The interaction networks provided the detailed information regarding the biological process and the pathways in which the role of characterized WRKYs has been reported. The other possible functional interaction partner for tomato WRKY proteins that were involved in the molecular mechanism of signaling and metabolic pathways at both cellular and systemic levels was searched and predicted from Predicted Tomato Interactome Resource (PTIR) database http://bdg.hfut.edu.cn/ptir/index.html [78] using comparative protein interactive network suggested for *Arabidopsis* WRKY33 interacting partners.

CATH/Gene3D Analysis

The WRKY protein sequences were analyzed for their structural classification using CATH server http://www.cathdb.info/ [79]. The CATH database provides the hierarchical classification of protein domains on the basis of their associated folding patterns for the entered query. The functional characterization of the query sequences was analyzed using FunFHMMer http://www.cathdb.info/search/by_funfhmmer web server [80] to evaluate their functional annotations based on gene ontological terms. Further, the ReviGO analysis, based on a hypergeometric distribution test was performed by fetching the identified GO IDs for characterizing their functional dimension on scattered plots using the ReviGO web server http://revigo.irb.hr/ [81]. The protein sequences were further verified for screening the properties of characterized WRKYs and their subcellular localization using CELLO2GO web server http://cello.life.nctu.edu.tw/cello2go/ [82].

## Results

### *In-silico* microarray analysis

**Fig 1:**
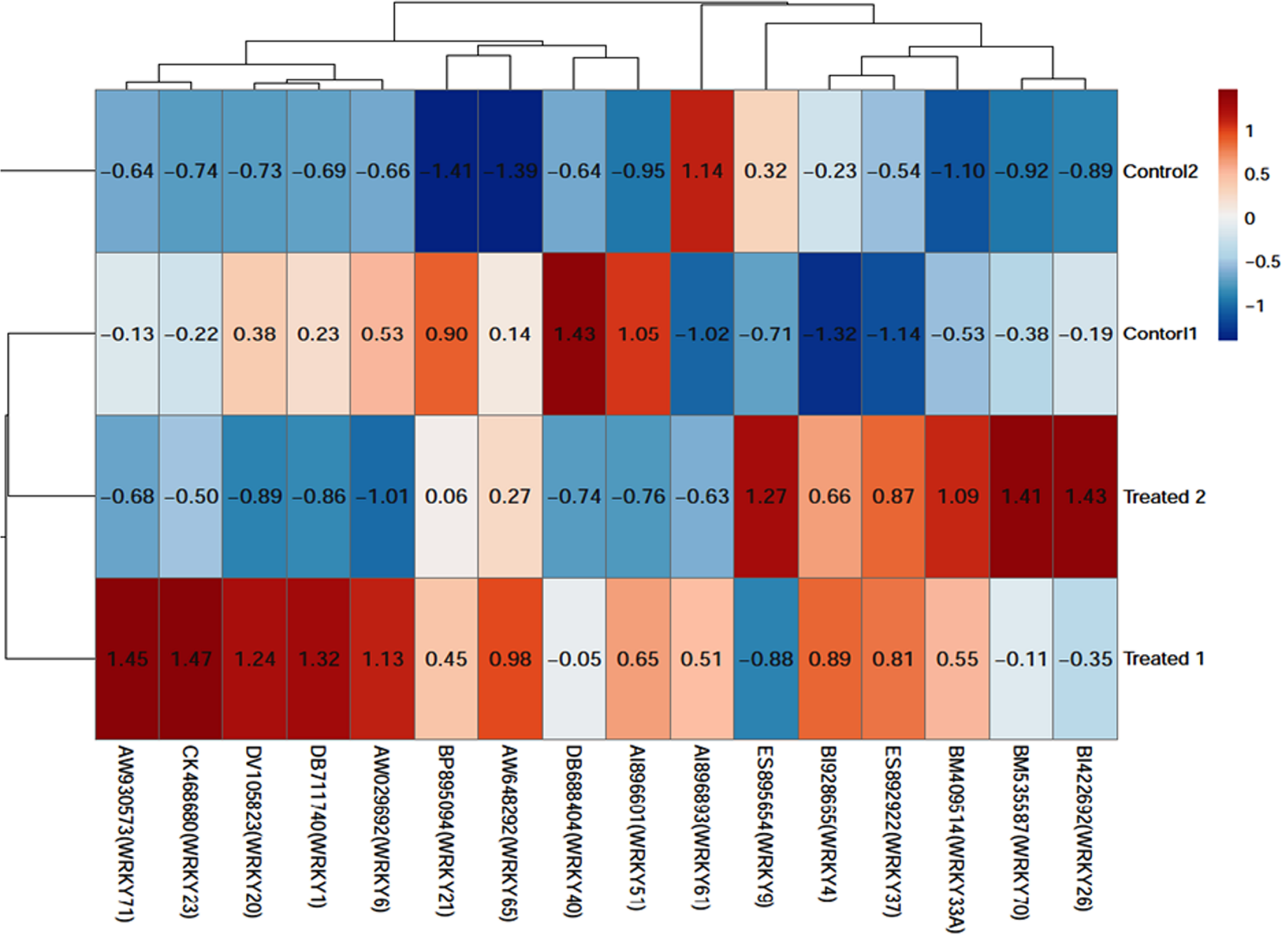
Heat map generated through ClustVis showing clustering of multivariate data values of differentially expressed and upregulated *WRKY* genes.

**Fig 2:**
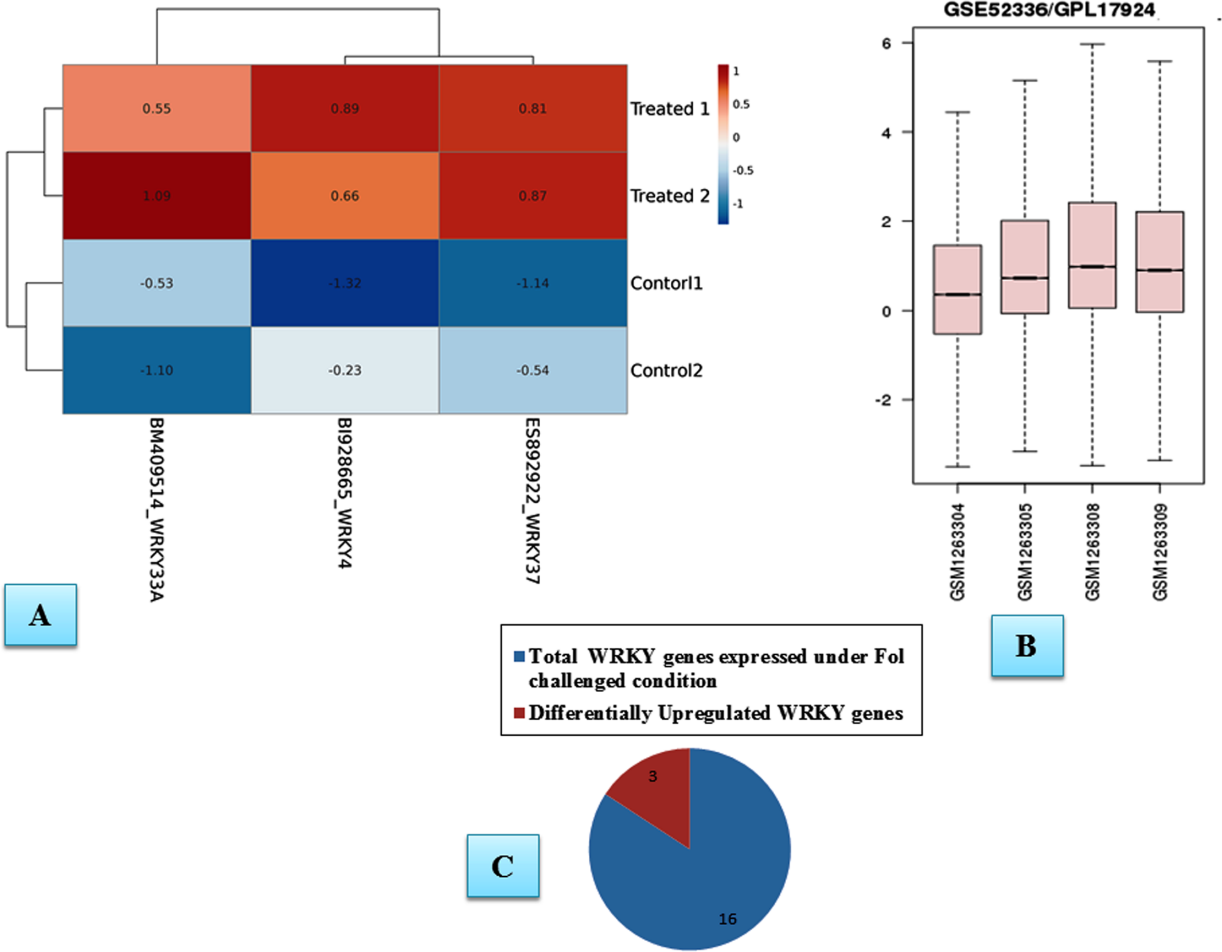
**A.** Heat map diagramme showing the differentially upregulated WRKY transcripts (SolyWRKY4, SolyWRKY33 and SolyWRKY37) **B.** Distribution of expression values for each selected samples (Two control GSM1263304_rep1 and GSM1263305 and two *Fol* challenged samples (GSM1263308_rep1 and 12623309_ rep2) represented in the form of box plot diagrame. The box plot diagrame for selected samples are not median centred (higher distribution values for treated samples compared to inoculated samples. **C.** The pie chart showing the number of differentially upregulated WRKY transcripts in tomato from total expressed *WRKY* genes under the *Fol* challenged conditions.

The genome-wide expression profiling by array showed that out of the total expressed transcripts only 16 transcripts from *WRKY* gene superfamily were found to be differentially expressed under *Fol* challenged conditions. Heat map of the differentially expressed and upregulated transcripts following the *Fol* inoculation have been shown (Fig 1). The heat map was based on clustering of multivariate data values using ClustVis The EST transcripts that were found to be differentially expressed under *Fol* challenged conditions were WRKY4 (EST accession ID: BI928665), WRKY33 (BM409514), WRKY37 (ES892922), WRKY40 (DB688404), WRKY23 (CK46868), WRKY70 (BM535587), WRKY71 (AW930573), WRKY26 (BI422692), WRKY51(BI209002), WRKY61 (AI896893), WRKY9 (ES895654), WRKY21 (BP895094), WRKY65 (AW648292) WRKY20, (DV105823), WRKY6 (AW029692) and WRKY1 (DB711740). The BLASTx results revealed the identity of an individual *SolyWRKY* transcript. The BiGGEsT gene expression analysis revealed all the differentially expressed *WRKY* transcripts (S1 Fig). However, among 16 expressed ESTs against *Fol* challenged conditions, only three WRKYs (*SolyWRKY4*, *SolyWRKY33*, and *SolyWRKY37*) were shown to have clear-cut differences in their expression profile and were found to show upregulation (red colour) in all the *Fol* challenged samples when compared to the healthy control samples (green colour) (Fig 2A) The GEO2R analysis revealed the distribution of expression values for each selected samples and was shown in the boxplot diagram (Fig 2B). The relative expression values shown on heat maps predicted the differential expression levels of the upregulated transcripts following the *Fol* challenged conditions compared to the untreated control samples. The pie chart representation was shown to represent the number of upregulated transcripts among the total number of differentially expressed WRKY transcripts (Fig 2C).

### Quantitative RT-PCR results

**Fig 3:**
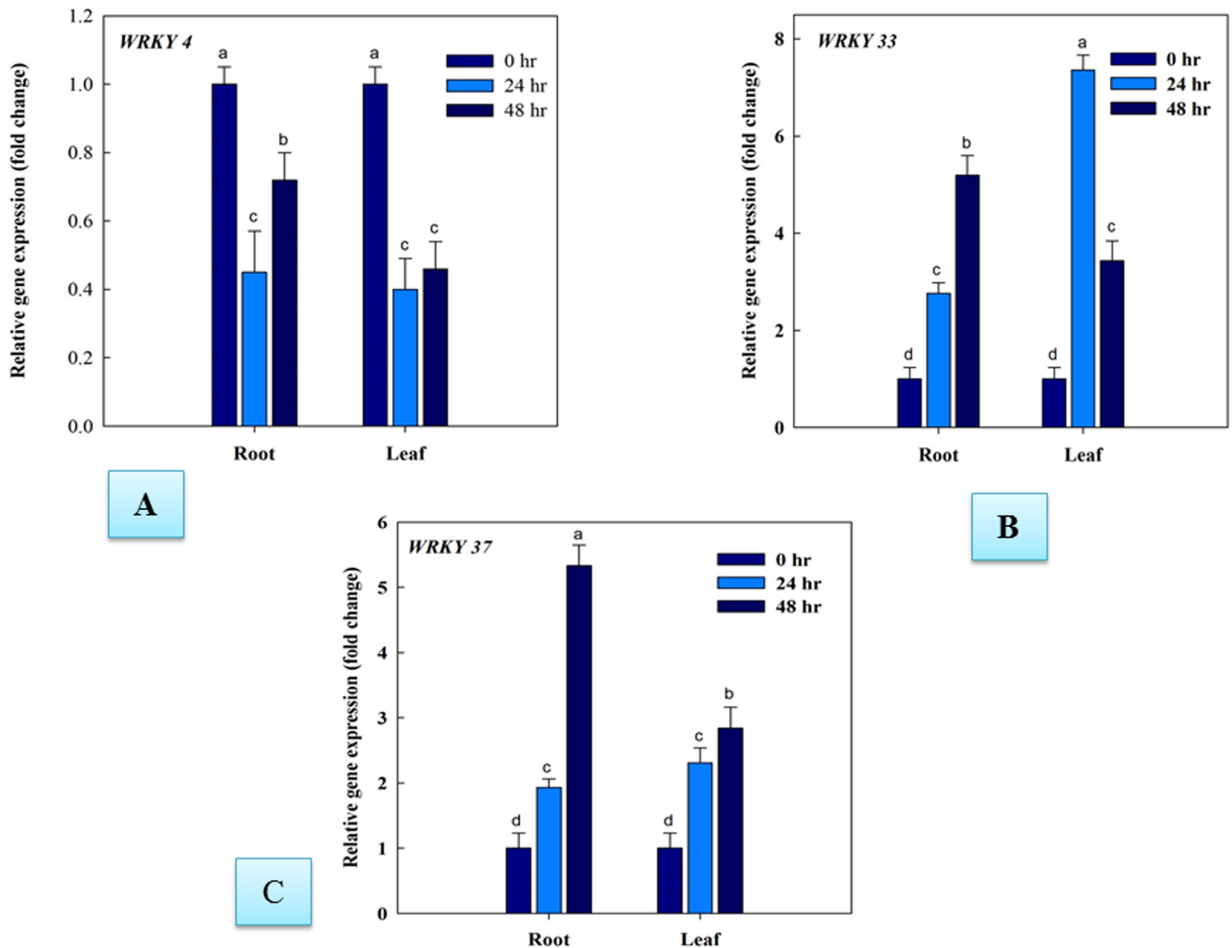
Quantitative PCR results showing the tissue specific differential expression of tomato *WRKY* genes expressed under the *Fol* challenged conditions at different time intervals (0, 24hrs and 48 hrs). The data represent the relative fold changes expression values of Fol treated samples compared to untreated (control) samples. The *SolyWRKY33* and *SolyWRKY37* genes were found to be upregulated as revealed through quantitative PCR. The *SolyWRKY4* genes were found to be downregulated in both root and leaf tissues.

**Fig 4:**
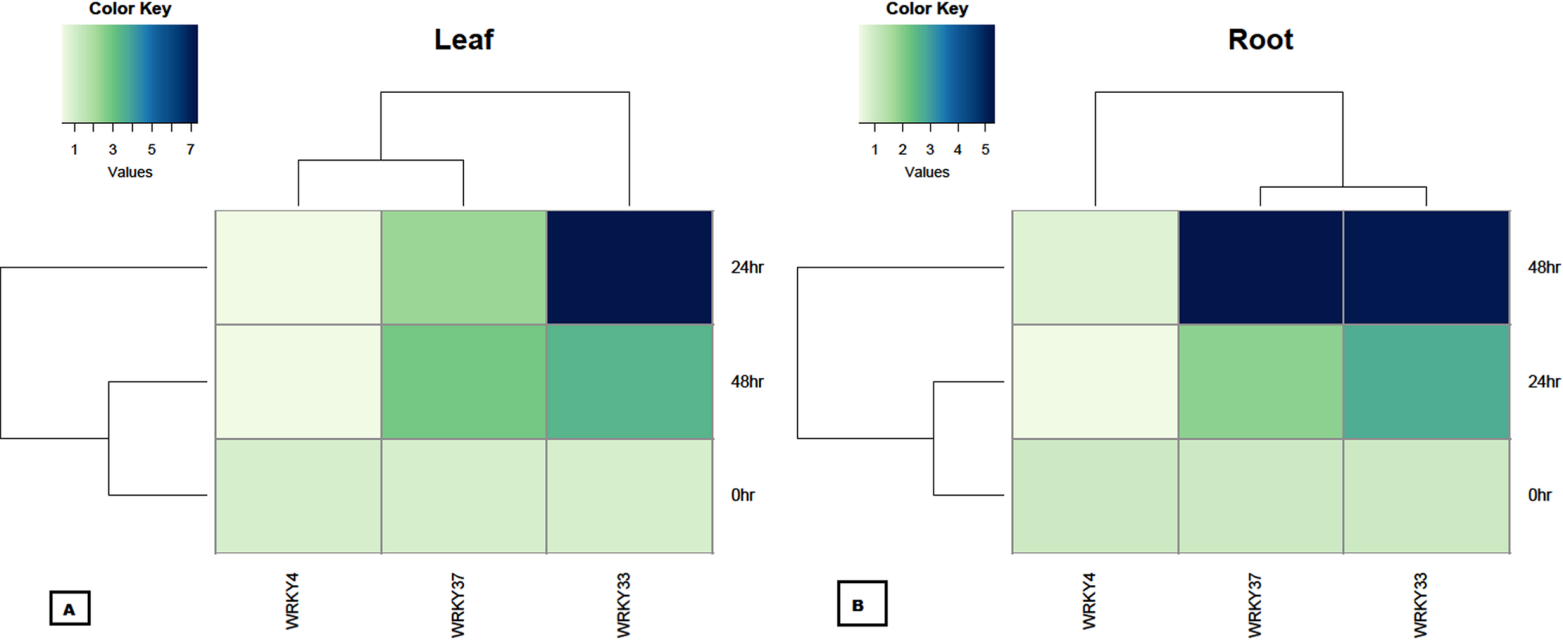
The differential expression of the replicated count data analyzed at different time interval and from different tissues. The heat map diagramme was generated through and Bioconductor R using the fold change expression values and were compared with control samples.

The quantitative RT-PCR (qRT-PCR) analysis was used to validate the microarray expression data only for those transcripts which had clear-cut differential upregulation in all the *Fol* inoculated samples among all the expressed WRKY members. The qRT-PCR analysis results revealed the distinct temporal and tissue-specific gene *WRKY* gene expression changes occurred during *Fol* challenged condition. Our results predicted that during *Fol* challenged condition in tomato increased expression of *SolyWRKY33* and *SolyWRKY37* gene, in infected root tissues was observed at 48hrs, (compared to uninoculated control) with maximum expression of *SolyWRKY37* (5.33 fold) and *SolyWRKY33* (5.19 fold). However, the expression of these genes at an earlier phase of infection (0–24 hrs) was comparatively less than that was found at 48 hrs. At an earlier phase of infection (0–24 hrs) the maximum expression of 2.76 fold increase in *SolyWRKY33* gene expression was recorded compared to *SolyWRKY37* (1.93 fold). In contrast, in leaf tissues, the maximum expression of the *SolyWRKY33* gene was observed at 24 hrs (7.36 fold) compared to *SolyWRKY37* (2.31 fold) and thereafter, a decreased expression was recorded at 48 hrs [*SolyWRKY33* (3.43 fold) and *SolyWRKY37* (2.84 fold)]. The qRT-PCR results validated our array based results for the expression of the *SolyWRKY33* and *SolyWRKY37* gene (Fig 3B and 3C). However, we got the repression (downregulation) of *SolyWRKY4* gene in qRT-PCR results in both root and leaf tissue samples (contrary to array studies where upregulation was recorded) under *Fol* challenged condition. It was found that the downregulation of *SolyWRKY4* gene in infected root tissues was more pronounced at 24 hrs (0.45 fold) which further decreased at 48 hrs (0.72) compared to control unchallenged samples whereas, in leaf tissues, the downregulation was found to be almost similar at 48 hrs (0.46 fold) (Fig 3A). Moreover, the tomato *WRKY* gene expression was found to be upregulated at increasing time interval from 0–48 hrs in all the treated tissues (root and leaf) in case of *SolyWRKY33*, *SolyWRKY37* and *SolyWRKY4* except for expression of *SolyWRKY33* in leaf tissues, where the relative expression (fold change) of *SolyWRKY33* gene was found to be decreased from 24–48 hrs. The qRT-PCR results confirmed the differential expression of the three identified, and upregulated transcripts following the *Fol* challenged conditions in different tissues. The heat map diagrame analyzed through Bioconductor R represented the relative expression values (fold changes) with respect to untreated control samples for tissue-specific (root and leaves) *WRKY* gene expression (Fig 4A and 4B) at different time interval under *Fol* challenged conditions.

### H_2_O_2_ generation, assessment of cell death and lignification

**Fig 5:**
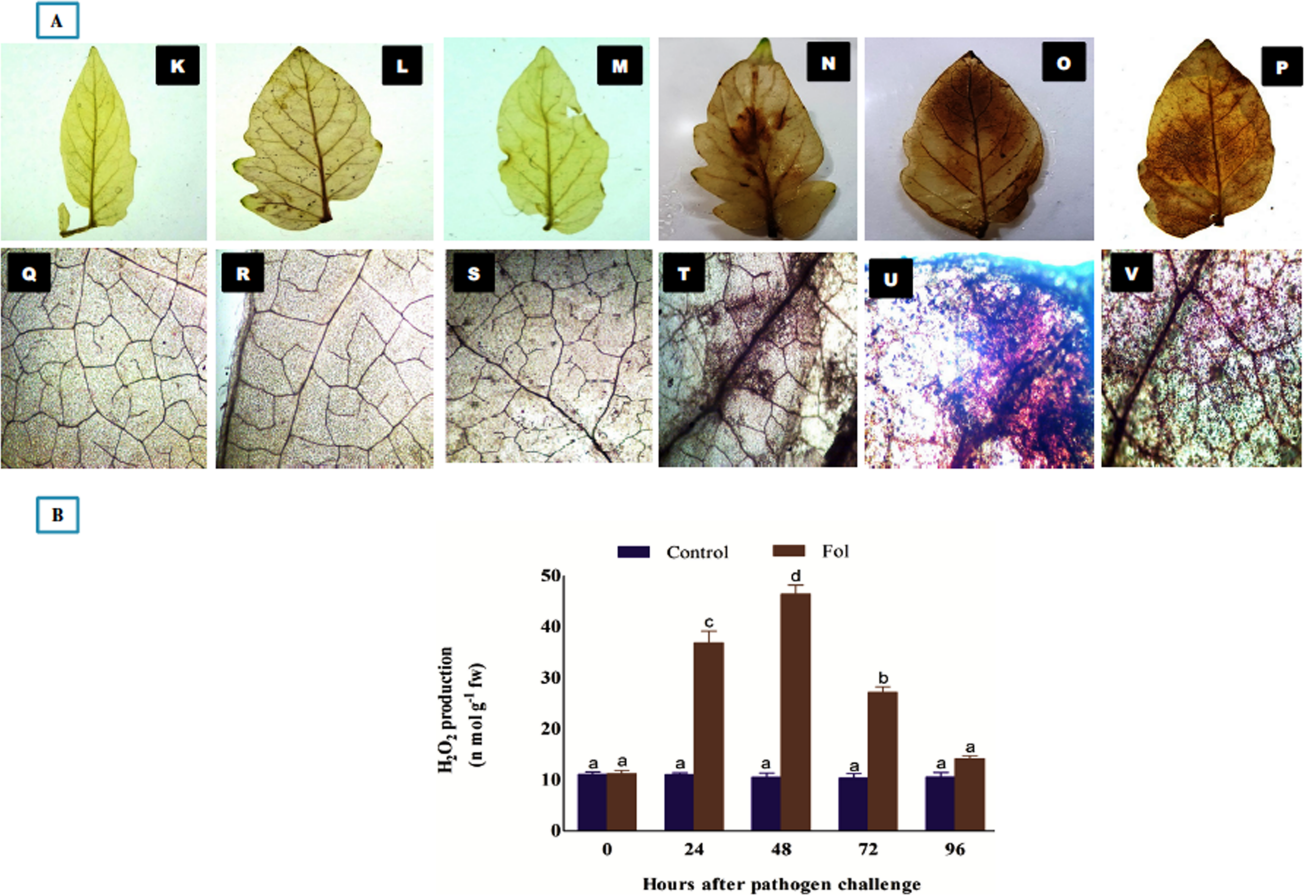
**A.** Histochemical staining for observation of accumulated H_2_O_2_ in control and *Fol* toxins exposed leaf tissues (DAB staining) at 48 hrs post inoculation of *Fol* toxins. **K, L and M.** General view of control leaf samples. **Q, R and S**. microscopic observation of control leaf tissues. **N and O**. *Fol* toxins exposed leaf tissues (attached) showing the accumulated H_2_O_2_ along the midrib, leaf margins and tips. **T and U**. Microscopic observation of the treated tissues accumulating higher amount of H_2_O_2_ along the midrib and leaf tips. **P and V**. Higher accumulation of H2O2 in leaf tissues (de-attached) **5B**. Biochemical assessment of H_2_O_2_ produced at different time interval. The H_2_O_2_ produced was higher at 24 hrs, become maximum at 48 hrs and decreases successively at increased time interval. The control tissues had more or less similar amount of H_2_O_2_ produced.

**Fig 6:**
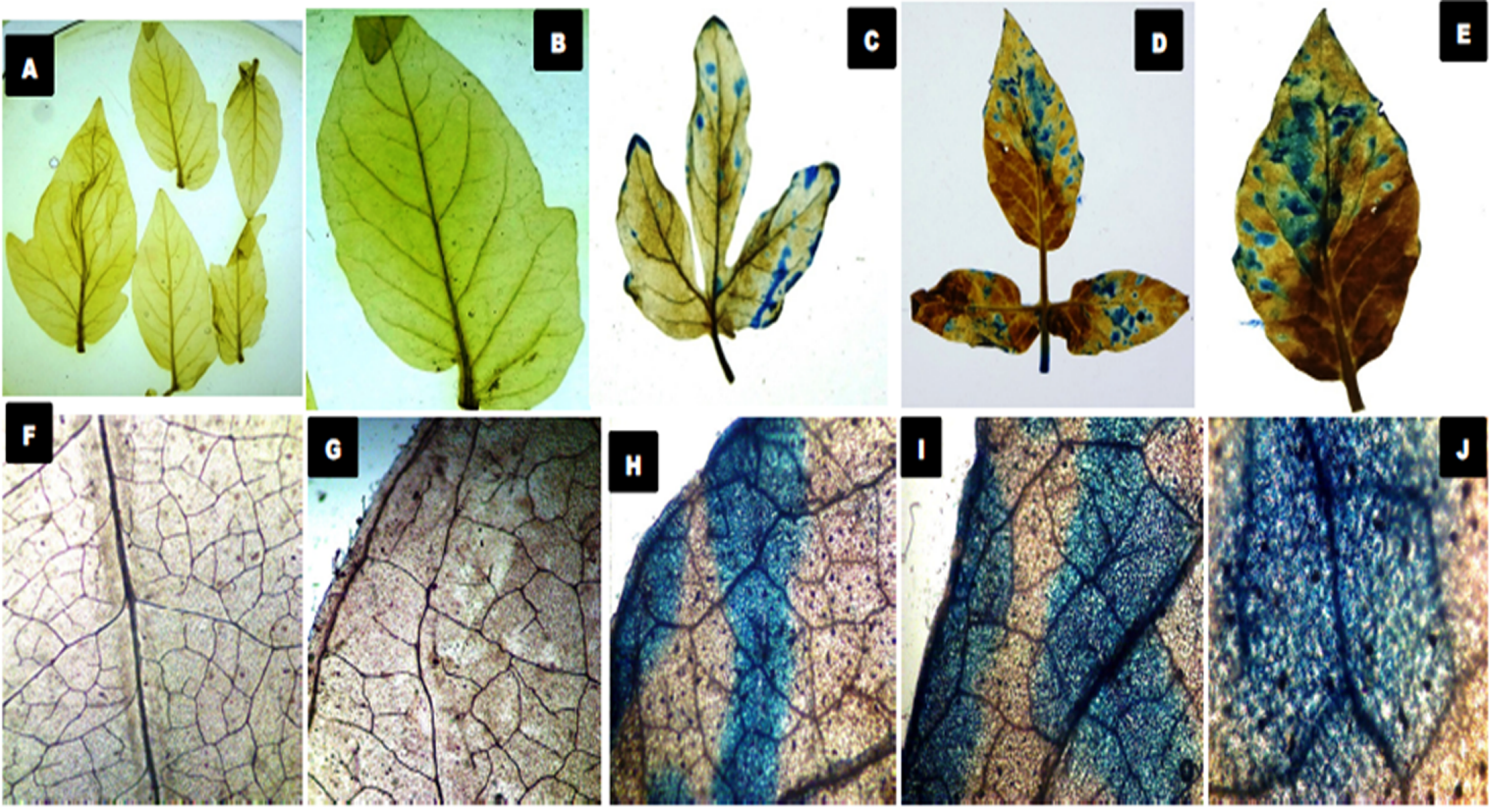
Histochemical analysis for the assessment of cell death. The leaves (both attached and detached with plants) were treated with culture filtrate of (*Fol*). The microscopic observation of the *Fol* toxins exposed necrotic lesions developed that eventually leads into cell death and was observed using Evan Blue stain. **A** and **B.** General view of control leaf samples. **C and D.** General view of *Fol* toxins exposed leaf tissues (attached) with having intense blue coloration showed the dead tissues. **E.** General view of *Fol* toxins exposed and unattached leaf tissue. **F** and **G.** Microscopic observation of control leaf samples. **H and I.** The microscopic observation of the leaf tissues (attached) showing the binding of Evans dye with dead tissues. **J** De-attached leaves observed at higher resolution.

**Fig 7:**
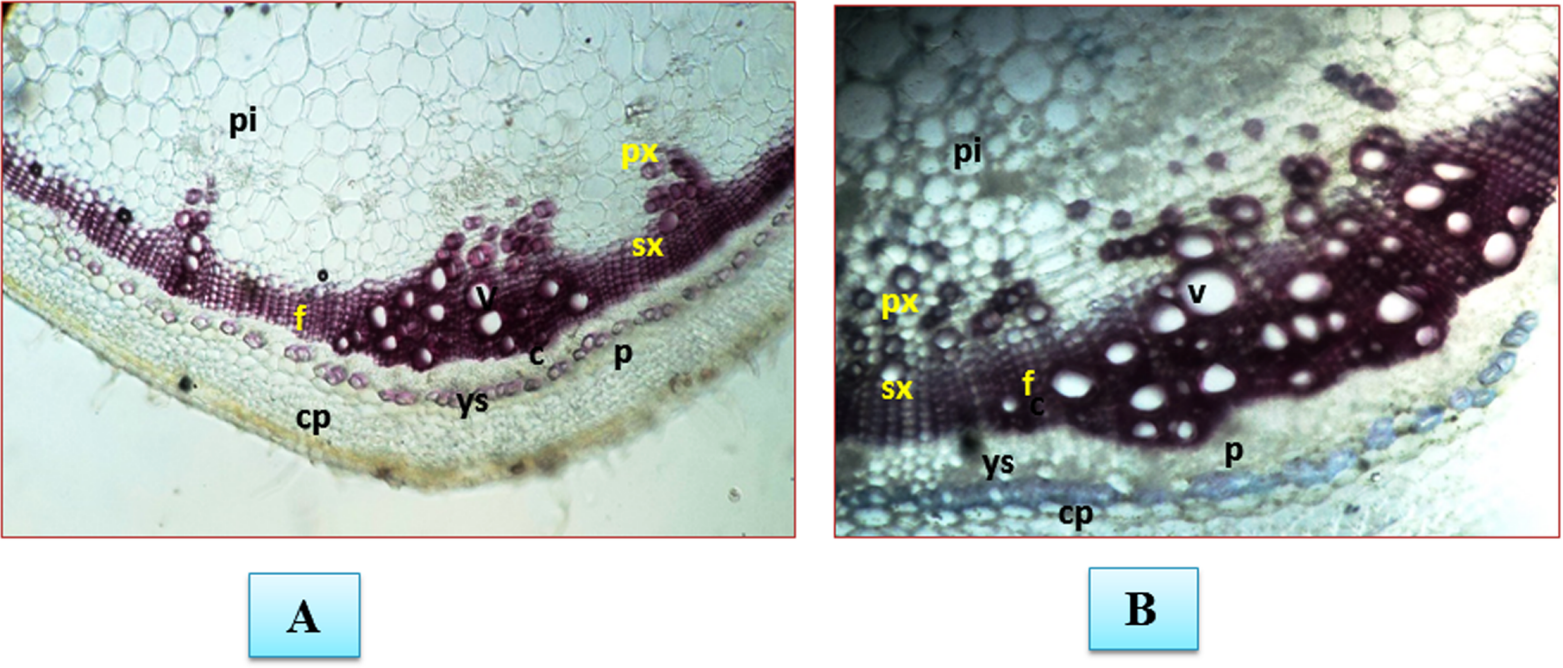
Assessment of plant defense response in the form of lignification. The pink colour shows the amount of lignified tissues. The shoot tissues between the second and third nodes from *Fol* challenged plants were collected after 3 weeks post inoculation. The photograph were taken after staining with phloroglucinol-HCl **A**. Control **B.** *Fol* challenged plants. Transverse section of tomato stem stained with pholoroglucinol-HCl at 2nd internode showing the lignified tissues in pink colour **px** = primary xylem; **sx** = secondary xylem; **f** = xylem; **p**i = pith; **p** = phloem; **c** = cambium; **v** = vessel; cp, cortical parenchyma. In control sample the amount of lignin deposition is less. The *Fol* challenged stem showed the intense pink coloration of the lignified tissues and the high intensity of pink colour in samples represent the high amount of lignified material deposited.

The sites for biochemical accumulation of H_2_O_2_ were visually analyzed through DAB staining. The H_2_O_2_ deposition sites were characterized by having an intense dark red-brown coloration in *Fol* treated samples (Fig 5A). It was observed that the maximum amount of H_2_O_2_ accumulated along the midrib of the leaf tissues. However, in some samples the *Fol* toxins exposed leaf tissues had the higher accumulation of H_2_O_2_ was also observed at leaf margin and leaf tip regions, as revealed from more reddish-brown coloration in these regions showed under microscopic view. The biochemical assessment of *Fol* induced biotic stress was measured in form of H_2_O_2_ produced in leaf tissues at 0, 24, 48, 72 and 96 hrs time interval. The amount of H_2_O_2_ was higher at 24 hrs post inoculation of *Fol* pathogen, becomes maximum at 48 hrs and then decreased successively on increasing time interval (Fig 5B) whereas the untreated control leaf tissue samples had more or less similar amount of H_2_O_2_ formed. The cell death was measured by histochemical microscopic observation of the necrotic lesions developed in *Fol* toxins exposed leaf tissues and untreated control samples. Evans blue dye was used as a marker for cell death (Fig 6). The plant cells from *Fol* challenged samples showed the light to the dark blue coloration of Evans blue stain. However, the intensity of blue coloration was found to be dependent on the extent of damaged tissues or dead cells present in between the healthy and live cells. The observed variation in color intensities was directly dependent on the extent of their destruction profile as the death cells took intense blue coloration. The transverse sections of tomato stem from *Fol* challenged plants under microscopic observation showed the increased amount of lignified tissues, as indicated by the pink color of phloroglucinol-HCl stained lignified tissues compared to control having a lesser amount of deposited lignins. The intensity of pink color intake represented the higher deposition of lignins. In control samples, the lignified material was much lesser (Fig 7A) than the *Fol* challenged stem (Fig 7B) where the higher amount of deposited lignins were found, and evidenced from their intense pink coloration.

### Identification of the functional domain in WRKY proteins

The Prosite results revealed the functional families to which each protein belong and was found to be the member of *WRKY* gene superfamily. The SolyWRKY33 Prosite scan analysis revealed the two functional WRKY domains (both at N-terminal and C-terminal end). In contrast, the SolyWRKY37 had only one WRKY domain. The results indicated the structural and functional diversity exists within the members which provides specificity to each member and that determines the *WRKY* gene-specific regulatory mechanism.

#### Analysis of WRKY domain

The InterProScan results further confirmed the presence of two WRKY-DNA binding domains at both N (IPR003657) and C terminal (IPR003657) ends in SolyWRKY33 (S7 Fig). In contrast, the SolyWRKY37 showed the presence of only one (C-terminal domain) (IPR003657) (S8 Fig). We reported the functional distribution of SolyWRKY33 and SolyWRKY37 proteins in two separate groups, SolyWRKY33 in group I due to the presence of two WRKY DNA binding domains and SolyWRKY37 in group II pertaining to the presence of only one WRKY domain. Furthermore, the distribution of zinc finger motif in the SolyWRKY33 at the NTD was found to be composed of C-X_4_-C-X_22_-HxH and the CTD contained C-X_4_-C-X_4_-H-X_18_-HXH. In contrast, the SolyWRKY37 zinc finger composition found was of the C-X_5_-C-KXV-HXH type, which further confirmed their allocation into the two separate groups.

#### Motif composition analysis

**Fig 8:**
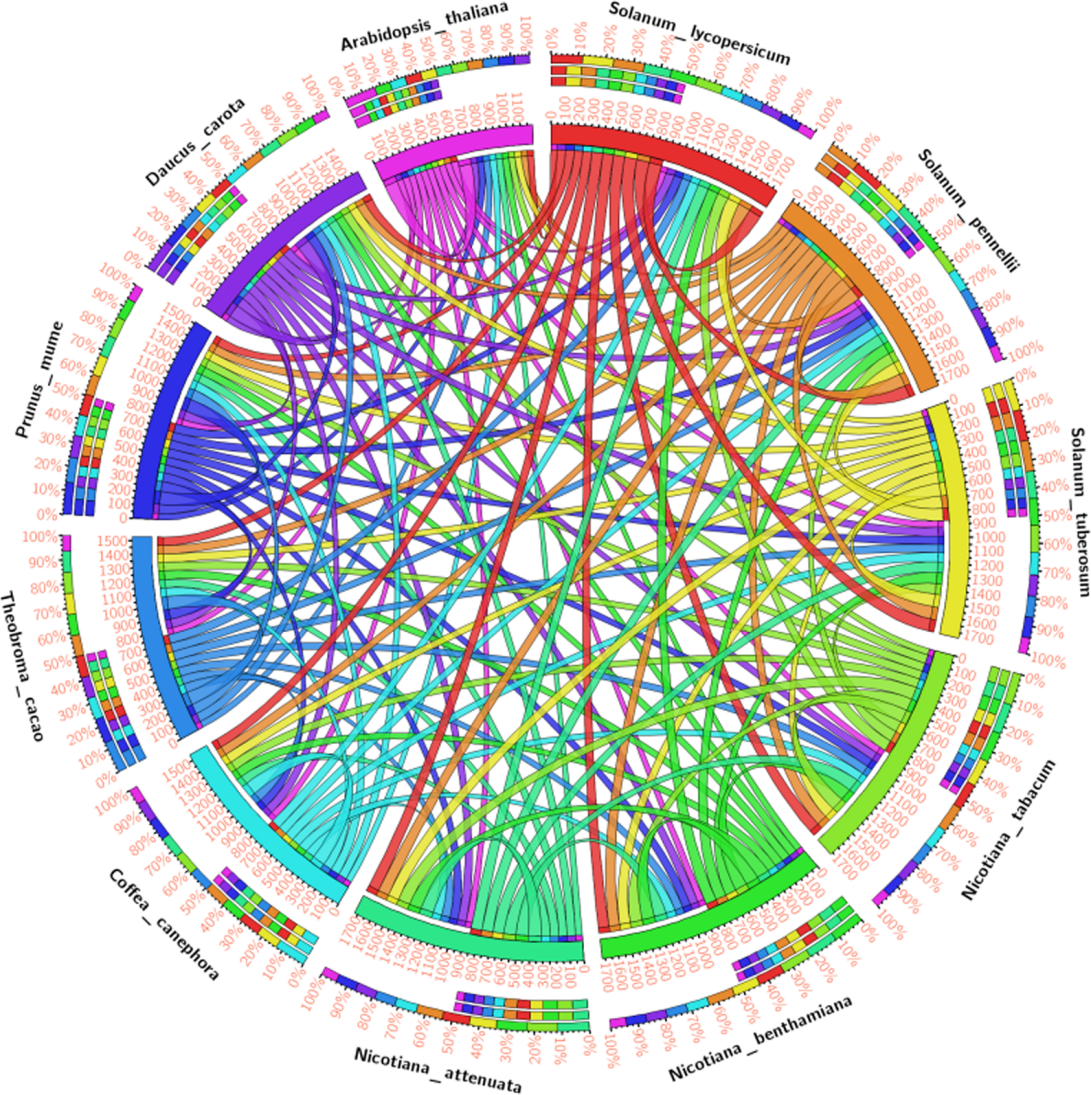
The circos genome visualization map for identifying the similarities and differences to identify the similarities and differences in SolyWRKY33 proteins as compared with other homologs and orthologs members and revealed through the comparative genomics. The map is based on the percentage identity matrices obtained during phylogenetic clustering of the sequences using Clustal W at 0% cut- off filter values. The thickness of the coloured band represents their respective relationship with other members.

**Fig 9:**
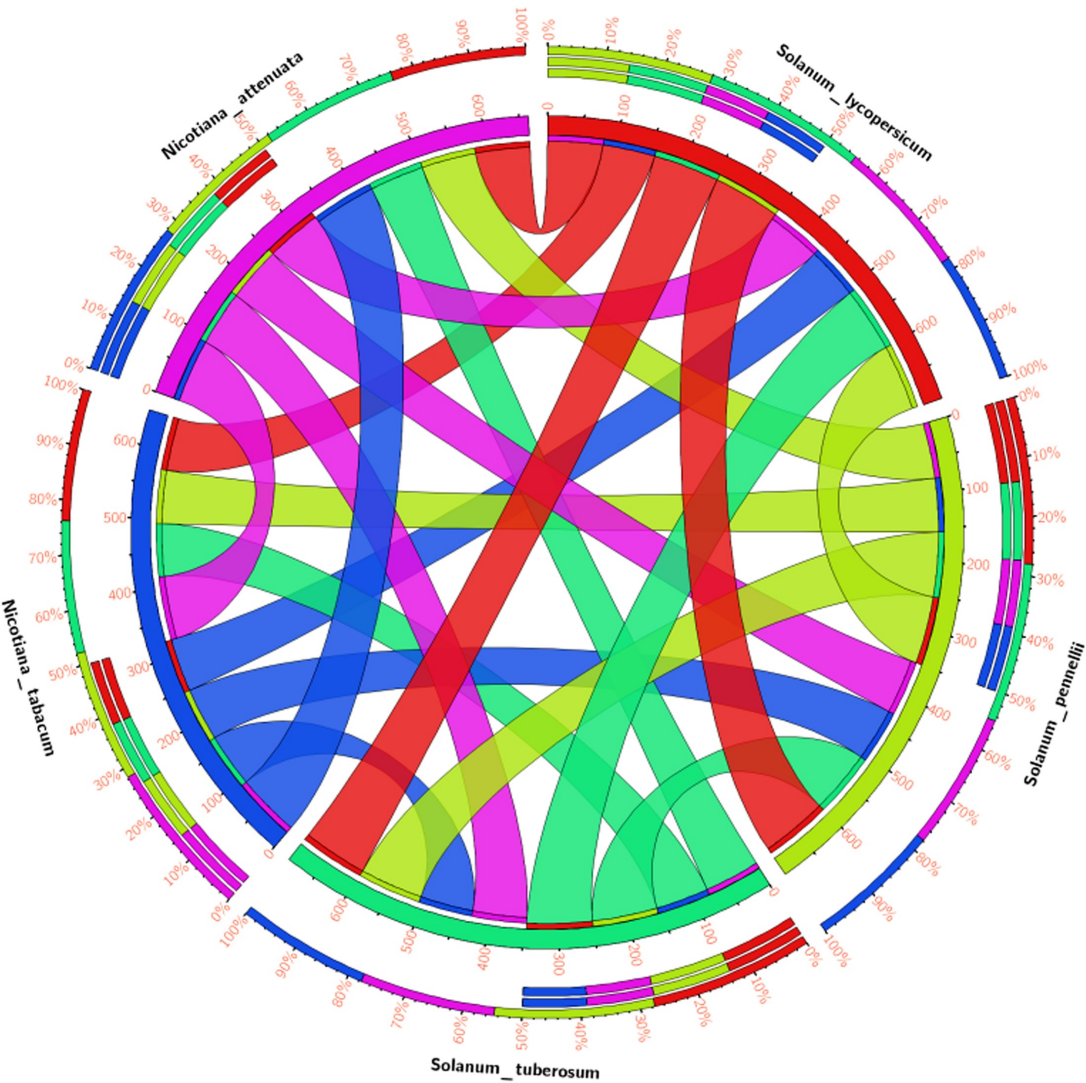
The circos visualization maps to identify the similarities and differences in SolyWRKY37 proteins as compared with other homologs members and revealed through the comparative genomics. The thick coloured bands for SolyWRKY37 with other members of tomato family predict their close phylogenetic relationship. The map is based on the percentage identity matrices obtained during phylogenetic clustering of the sequences using Clustal W at 0% cut- off filter values. The thickness of the coloured band represents their respective relationship with other members.

The MEME motif scan analysis revealed the presence of uniform motifs across all the members in SolyWRKY33. The phylogenetic studies revealed the monophyletic origin of SolyWRKY33 from its wild homolog SpWRKY33 whereas; StWRKY33, NaWRKY33, NbWRKY33 and NtWRKY33 were shown to have a paraphyletic origin with SolyWRKY33 (S2A Fig). The similar results were found with respect to motif distribution characteristics where SolyWRKY33, SpWRKY33 and StWRKY33 have been found to share the common motifs across the entire length of the protein. The motif scan through MEME suite analysis revealed the presence of significant motifs that constitute the functional WRKY domain and could fold to form a complete functional four stranded β sheet that is involved in WRKY W-box DNA(TTGACC/T) interaction to mediate the WRKY specific gene regulation. The significant motifs involved in this four stranded β sheets were WRKYGQKQVK (forming β1) strand), NPRSYYKCTY (forming β2 strand), CPTKKKVER (forming β3; dominated by lysine substitutions), and lastly VITTTYE motif (forming β4) (S2B and S2C Fig). Moreover, these residues were found to be conserved across along the SolyWRKY33 homologs which reflect their crucial role for effective and more feasible DNA-protein interaction. The phylogenetic tree revealed the clustering of SolyWRKY37 with other homologs of tomato family with its monophyletic origin from SpWRKY37 (S3A Fig) with more similar and uniform motif distribution (S3B Fig). However, SolyWRKY37 becomes separated from NaWRKY37 and NtWRKY37 due to the presence of two uncommon motifs VQQEENQFTD and LDPVTQDSAM. The motif logo diagramme for SolyWRKY37 have been shown (S3C Fig) contain the functional WRKY domain. The circos analysis revealed the similarities and differences observed in between the different WRKY members. The circos results identified the similarities and differences observed in between the SolyWRKY33 and other ortholog members, which revealed that the SolyWRKY33 is more conserved and have more divergent ancestral evolutionary origin (Fig 8). In contrast, circos results for SolyWRKY37 (Fig 9) have shown that this group (group II-e) of WRKYs have been recently evolved in tomato family.

#### Database search and comparative phylogeny

The protein sequences for the upregulated WRKY transcripts SolyWRKY33 (NCBI accession ID: NP_001306910.1) and SolyWRKY37 (NCBI ID: NP_001308885.1) were further used for the comparative phylogenetic study, sequence alignment, motif scan. The phylogenetic tree is constructed based on the best significant matches (percent identity), and was further confirmed through aligning the query with subject sequences. The sequential homolog, and orthologs for SolyWRKY33 (S4 Fig) and SolyWRKY37 (S5 Fig) have been shown. The multiple sequence alignment of the WRKY domain (60 amino acids) for the different homolog and orthologs show the strong conservation of the residues which indicate their crucial function in WRKY dependent signaling and gene regulation at the molecular level (S6 Fig).

#### Protein-protein functional interaction network

**Fig 10:**
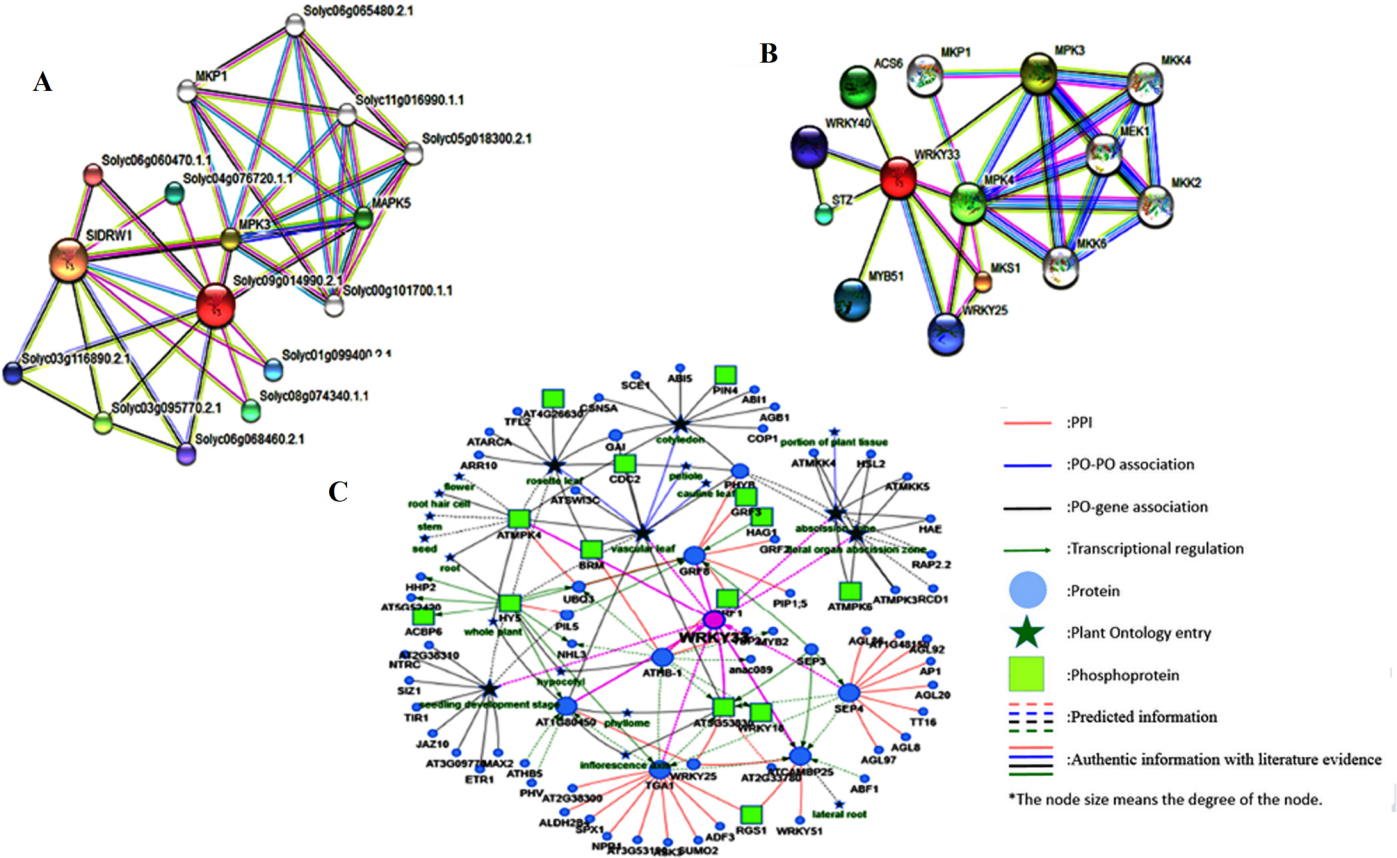
**A.** Interaction network of WRKY33 protein in *Arabidopsis* homologue of tomato. The color nodes represent the query protein and first shell of interactors whereas; white nodes are second shell of interactors. The large node sized interactors represent those proteins which have been well characterized whereas the small node sizes represent proteins uncharacterized **B.** Functional associative network of WRKY 33 protein in tomato **C.** The *Arabidopsis* WRKY33 protein interactive network obtained from Predicted Tomato Interactome Resource (PTIR) database.

Since the majority of proteins in tomato are still uncharacterized or unannotated, we have provided a comparative analysis of different protein interacting partners for the SolyWRKY33, *Arabidopsis* WRKY33 and the tomato WRKY33 homologs for *Arabidopsis* to unravel the actual functional interaction network. All the possible interacting partners at highest confidence level for the SolyWRKY33 was explored through the functional analysis of tomato homologs for *Arabidopsis* WRKY33 at their highest confidence level, and were further compared with the network available for SolyWRKY33 as retrieved from STRING database given at medium confidence level (since at high to highest confidence level we do not find all the interacting partners). Further, in our results, we showed the interactive network involved in SolyWRKY33 hormonal signaling at medium to high confidence level (Fig 10A) compared with tomato WRKY33 homologs for *Arabidopsis* (was shown to take at highest confidence interval) (Fig 10B). The results obtained through these multiple backgrounds on STRING server were further compared with the *Arabidopsis* WRKY33 visual rich club connectivity diagrame, and revealed through Predicted Tomato Interactome Resource (PTIR) database (Fig 10C). The protein-protein interaction data and interactome network analysis of AtWRKY33 (PTIR database) revealed the most probable and possible interaction network for tomato WRKY protein (SolyWRKY33). Overall the protein partners forming the functional interactive network for tomato WRKY33 could be deduced from these interlogs based protein-protein interactions [81]. The protein interacting partner for the SolyWRKY33 includes the sigma factor binding protein 1 (having identifier Solyc01g096510.2.1: NCBI-Protein ID: XP_004230035) involved in the plant defense and may regulate chloroplast metabolism upon infection with necrotrophic pathogens and homologous to the SIB1 protein of *Arabidopsis* (AT3G56710.1) MKS1 like protein (having identifier Solyc06g060470.1.1: XP_004242290) having a crucial role in the plant defense through MPK4 regulated defense activation that works through coupling with the kinases to specific WRKY transcription factors, and were found to be MKS1 homologs of *Arabidopsis* (AT3G18690.1). Mitogen activated protein kinase (MAPK5 having identifier Solyc01g094960.2.1: NP_001234266 and MPK3 having identifier Solyc06g005170.2.1: NP_001234360) involved in the plant defense response and were homologous to MPK4 (AT4G01370.1) negative regulator of the systemic acquired resistance (SAR) and positive regulator of JA mediated signaling and MPK3(AT3G45640.1) and involved in oxidative stress mediated signaling, C_2_H_2_-type zinc finger protein (CZFP1) (having identifier Solyc04g077980.1.1: NP_001234718), WRKY transcription factor family proteins WRKY70 (having the identifier Solyc03g095770.2. XP_004235231), WRKY1 (having identifier Solyc06g068460.2. NP_001304176), WRKY40 (having identifier Solyc03g116890.2.1: NP_001303844) and homologous to AtWRKY40 (AT1G80840.1) were found to be involved in the interactive associative network with the SolyWRKY33 (having identifier Solyc06g066370.2.1).

#### Computational modelling of functional WRKY domain

**Fig 11:**
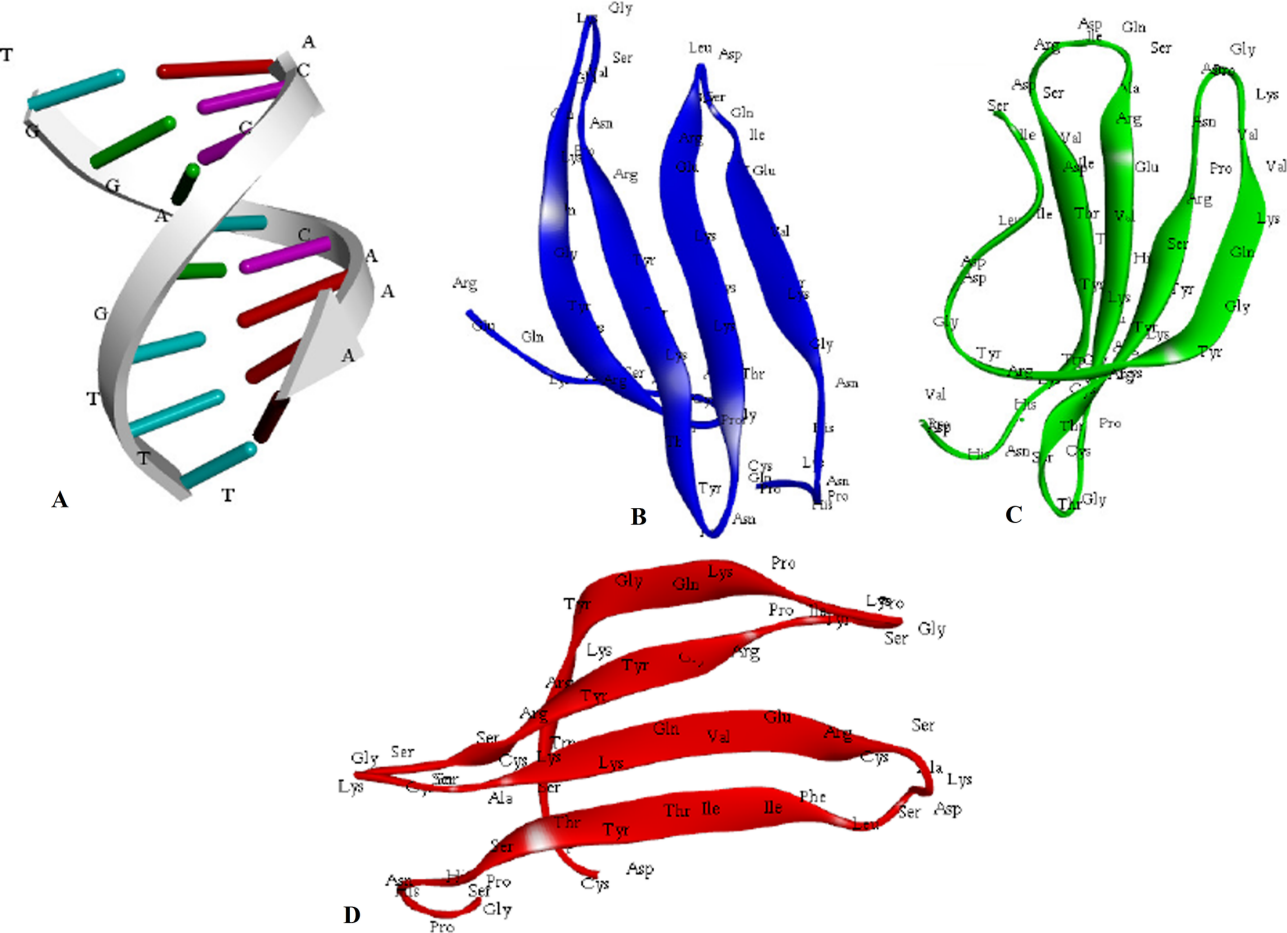
**A.** Structural modeling of the functional domain of SolyWRKY33 protein at both N-terminal and C-terminal end and W-box DNA using DS Modeller **A.** Structure of the modeled W-box DNA **B.** The N-terminal SolyWRKY33 domain (NTD) **C.** The C-terminal SolyWRKY33 domain (CTD) **D**. The SolyWRKY37 WRKY domain.

The electrostatic energies provide by far the best structure–function and were reported as the most effective tool for correlating the structures of proteins and other macromolecules [83] as revealed by the quantitative studies of X-Ray or NMR derived structures. The protein stability was considered to be good based on the total minimum electrostatic energy [84]. The specifically recognizedW-box sequence 5’(T)(T)TGAC(C/T)A3’ [28] was used for the computational modelling of the WRKY specific W-box DNA motif using the sequence to structure tool (http://pongor.itk.ppke.hu/dna/model_it.html#/modelit_intro; (Fig 11A) [85]. The domain structure for the SolyWRKY33 NTD (Fig 11B), the SolyWRKY33 CTD (Fig 11C), and the SolyWRKY37 CTD (Fig 11D) was modelled through the DS Modeller Client 3.0. A total five models (each for SolyWRKY33 N-Terminal Domain (NTD) (Table 2), SolyWRKY33 C terminal domain (CTD) (Table 3), and SolyWRKY37 (Table 4) were predicted. The loop modelling was used to remove the loop regions aroused from sequential insertions, or that deviates the target structure from template proteins. The protein models having least energy score value and RMSD deviations around the Cα atoms were further refined and optimized from Cα traces, and based on a two-step, atomic-level energy minimization, and to eliminate the residues from disallowed regions using ModRefiner server http://zhanglab.ccmb.med.umich.edu/ModRefiner/ [86]. Further, the predicted model was evaluated with NMR determined CTD protein structure of AtWRKY4 that comprised of the protein moiety of the complex consisted of a four-stranded β-sheet (β1, Trp-^414^–Val^422^; β2, Tyr^427^–Thr^436^; β3, Cys^439^–Arg^447^ and β4, Val^455^–Glu^460^ (motif logo). Interestingly, the similar topological conformation having four stranded β sheet including (β_1_, Trp^383^ –Val^391^; β_2_ Asn^396^- Ser^405^ (with Tyr ^427^ being substituted by residues Asn^396^ and Thr^436^ by the residue Ser^405^); β_3_ Cys^408^- Arg^416^ and β_4_ Val^424^- Glu^429^ was found in our modelled CTD. Furthermore, the result of motif composition analysis revealed the strong conservation of these four strands across all the members in the tomato family. The protein models predicted were further submitted to PMDB (https://bioinformatics.cineca.it/PMDB/) [87] and were provided with submission identities including PM0080926 (WRKY33_NTD) and PM0081236 (WRKY33 NTD), PM0080775 (WRKY33 CTD) and PM0080776 (WRKY37) (S7 Fig).

**Table 2:** Different energy parameters for the predicted and modelled N-terminal domain of SolyWRKY33 measuring their structural stability based on DOPE score, PDF physical energy and PDF total energy. DOPE score for five analogues available for modeled SolyWRKY33protein and were arranged based on their high DOPE score and low molpdf values.

MODEL SCORES			
Model Name	PDF Total Energy	PDF Physical Energy	DOPE Score
MODEL1(M0001)	405.3383	204.72282	**-**4091.337646
MODEL3(M0003)	419.9820	207.8125444	**-**4050.627686
MODEL5(M0005)	436.0580	205.69352	**-**3999.065918
MODEL4(M0004)	457.9554	213.63804	**-**4107.419922
MODEL(M0002)	518.0856	214.90768	**-**4006.610596

**Table 3:** Different energy parameters for the modelled C-terminal domain SolyWRKY33 to measure their structural stability and DOPE score, PDF physical energy and PDF total energy. DOPE score for five analogues available for modeled SolyWRKY33 protein and were arranged based on their DOPE score values. Loop refinement was used for increasing the structural stability and reliability of the modelled domain structure. The loop refined models were further arranged based on their minimum energy profile.

MODEL SCORES			
Model Name	PDF Total Energy	PDF Physical Energy	DOPE Score
MODEL3(M0004)	486.3257	196.41296	- 3909.786377
MODEL2(M0002)	503.0581	197.60207	- 3714.846191
MODEL1(M0005)	530.0815	209.74898	- 3951.424805
MODEL5(M0001)	551.2874	207.94684	- 3762.376953
MODEL4(M0003)	567.6835	242.70571	- 3898.345703
**LOOP MODEL SCORES**			
MODEL5(M0003L0001	-1355.5048	- 1233.331295	- 4008.651855
MODEL4(M0004L0001)	- 1325.4363	-1207.3277	- 3994.355957
MODEL3(M0005L0001	- 1283.6738	-1218.698538	- 3955.281738
MODEL1(M0002L0001)	- 1247.1262	- 1155.725	- 3823.847412
MODEL2(M0001L0001)	- 1171.3962	-1108.58191	- 3788.588135

**Table 4:** Different energy score values including DOPE score, PDF physical energy and PDF total energy for the modelled WRKY37 domain structure. Loop refinement was used for more refinement of the modelled protein and the refined models were further arranged based on their minimum energy score values.

MODEL SCORES			
Model Name	PDF Total Energy	PDF Physical Energy	DOPE Score
MODEL3(M0003)	296.7447	171.118258	- 4106.798340
MODEL2(M0002)	297.5872	167.988204	-4074.438232
MODEL1(M0001)	306.1449	169.599006	-4007.020996
MODEL5(M0005)	311.8783	165.885920	-4067.071289
MODEL4(M0004)	323.8305	169.284177	-4016.324707
**LOOP MODEL SCORES**			
MODEL3(M0003L0001	-1442.3384	-1294.864	-4161.322266
MODEL4(M0004L0001)	-1348.4739	-1221.818088	-4051.130859
MODEL5(M0005L0001	-1292.9580	-1202.99	-4044.156738
MODEL2(M0002L0001)	-520.6688	-820.6483	-3748.926758
MODEL1(M0001L0001)	-383.7734	-756.9299946	-3697.743164

#### Model analysis and validation

**Fig 12:**
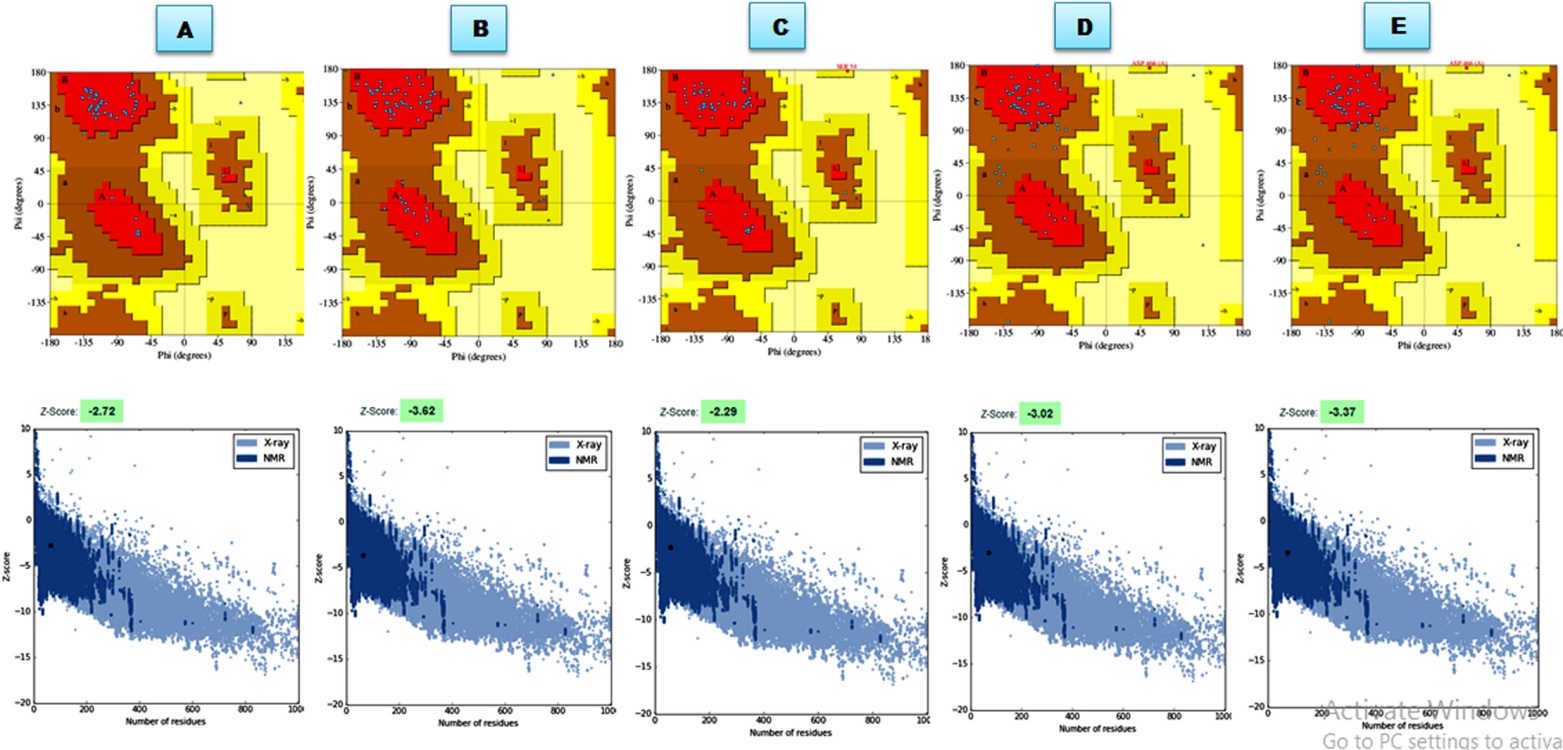
The stereo chemical spatial arrangement of amino acid residues in the modeled domain structure for SolyWRKY33 NTD **A.** The modeled SolyWRKY33 CTD **B.** modelled SolyWRKY37 **C.** The results were compared with Ramachandran plots available for experimentally deduced structures (template) Solution Structure of the C-terminal WRKY Domain of AtWRKY4 (1WJ2) **D.** Crystal Structure of the C-terminal WRKY domain of AtWRKY1 (2AYD) **E.** The plot calculations on the 3D model of WRKY proteins were computed with the PROCHECK server. Most favoured regions are coloured red, additional allowed, generously allowed, and disallowed regions are indicated as yellow, light yellow and white fields, respectively.

The five different protein models each for SolyWRKY33 and SolyWRKY37 were predicted. The 3D models generated by modeller is predicted based on satisfying the spatial restraints derived from sequential alignment and optimization of the molecular pdf obtained through DS Modeller and was expressed as probability density functions (PDFs). Further, the models generated were statistically measured from their Discrete Optimized Protein Energy (DOPE) score [88]. The DOPE score values for the generated models SolyWRKY33 were reported in Table 2 and Table 3. Further the models having high DOPE score and least values of mol pdf were considered to be structurally stable and reliable in terms of their energy values. In our results, the two different protein models both for NTD and CTD (SolyWRKY33) were generated. In case of SolyWRKY33 NTD, the model with having DOPE score **-**4091.337646 and mol pdf value 405.33839 (model 1) and the model with DOPE score -4008.651855 and mol pdf -1355.5048 (model 5) for SolyWRKY33 CTD was selected. For SolyWRKY37 the model having DOPE score -4161.322266 and mol pdf -1442.3384 (model 3) was selected as final model for further studies. The selected models were further evaluated based on their qualitative and quantitative energy parameters. The qualitative assessment of the selected protein models was determined based on the Ramachandran plot statistics using RAMPAGE and PDBsum web server for all the possible and allowed conformations of their phi (Φ) and psi (Ψ) angles. The qualitative assessment to check the reliability of predicted models Ramachandran plot analysis and statistics reported that NTD SolyWRKY33 occupied 100% residues lying in the favoured regions, with no (0.0%) residues in the additionally allowed and disallowed regions (S8A Fig). However, the predicted CTD SolyWRKY33 comprised of 98.4% residues occurring the most favoured regions (A, B, L) with 1.6% residues favouring for additionally allowed regions (a, b, l, p) (against the expected values 98% (favoured) and 2.0% (additionally allowed regions). In contrast, the selected protein model SolyWRKY37 had 98.3% (favoured) and 1.7% residues lying in the additionally allowed segments. However, the qualitative assessment through PDB sum server and based on PROCHECK analysis we found that 96.3% residues in most favoured, 3.7% additionally allowed and 0.0% in the generously allowed regions and 0.0% in the disallowed regions (XX) with the G factor values (-0.02) for the SolyWRKKY33 NTD (Fig 12A). Similarly, the SolyWRKY33 CTD we reported 94.6% residues lying in the most favoured, with 5.4% additionally allowed and 0.0% in generously allowed and disallowed regions with having G factor values (-0.16) (Fig 12B) (contrary to it for SolyWRKY37 the observed values were 93.9% residues in the most favoured, 4.1% additionally allowed and generously allowed regions with 2.0% in the disallowed regions with having G factor values (-0.12) (Fig 12C). All the selected models were further compared with the experimentally deduced (X-ray diffracted crystal structures or NMR derived solution structure) to evaluate their stability and reliability in terms of qualitative and quantitative parameters. The modelled domain structure had better qualitative and quantitative score values when compared to the NMR derived structures (AtWRKY4 PDBID: 2LEX) where we found that 77.8% residues occupied the favoured regions, 14.8% occurring inside the additionally allowed and 2.2% occupying the disallowed regions (Table 5). The CATH server predicted that both proteins SolyWRKY33 and SolyWRKY37 possess the secondary structure with having beta sheet type topology, and which was further validated qualitatively using VADAR (Volume Area Dihedral Angle Reporter) results [89]. The VADAR results provided the quantitative aspects of the modelled SolyWRKY33 domain. The topological orientation of the functional SolyWRKY33 domain structure was found to have observed values of 0(0%), helix, 33(46%) beta, coil 38 (53%) and turns 04 (05%) against expected values with mean H bond energy (-1.6 SD = 0.5) against the expected score values of -2.0 (SD = 0.8). The secondary structure assignment of the functional WRKY protein was analyzed using the DALI web server (http://ekhidna.biocenter.helsinki.fi/dali_server/start) [90]. The DALI results revealed that in the functional protein sequence 40.84% residues occupied or constituted the β strands. However, some residues in the functional protein such as “VQTTSDIDILDDGYR”, “KYG”, “VVKGNPNP”, “SQDIR”, and “GKHNHDVPA” that folded to occupy the loop regions. In contrast, other residues WR, QK, RSYYKCTS, CPVRKHVERA, and SVITTYE formed the strand regions. The minimum deviation was observed for SolyWRKY37 with 0 (0%), helix, 31 (51%) beta, coil 29 (48%) and turns 08 (13%) and having mean H bond energy (-2.2 SD = 0.9) against the expected values of -2.0 (SD = 0.8). The reliability and the stability of the predicted models were validated and verified using qualitative assessment tools such as PROCHECK [91], QMean [92], ProSA [93], Verify-3D [94] ERRAT [95]. The PROCHECK measured the stereo chemical quality of the modelled protein, on the behalf of main-chain bond lengths and bond angles. While ProSA evaluates the model quality based on probable residues lying at specific distance and interactions observed between the model and the solvent (i.e., solvation) (Fig 12B). The qualitative assessment of the predicted models using QMEAN server based on six parameters including local geometry (assessed by torsion angles), distance between atoms, the burial of residues, with the two terms describing the and solvent accessibility between our predicted and calculated secondary structures (S9 Fig). In contrast, the Verify-3D assessed the quality on the basis of probable secondary structures and the topological orientation of the buried section and polar contacts. The assessment of protein models in terms of their structural stability and reliability could be determined based on parameters such as percentage residues occupied in the most favoured and disallowed regions, and a good model was shown to have over 90% residues in the most favoured regions [A, B, L]. Moreover, ProSA score confirmed that the minimal structural differences in between template and predicted proteins as revealed through their scores values. The ERRAT score evaluated the qualitative efficacy of the predicted models based on non-bonded atomic interactions and corrected atom distribution with respect to each other in the predicted protein. The ERRAT score values obtained for the functional CTD (SolyWRKY33) were 73.684%, 90.909% (for NTD SolyWRKY33) and 64.706% (SolyWRKY37 CTD) (S10 Fig). The protein coordinates that determines the stereochemical qualities of predicted models were found to be stable and reliable.

**Table 5:** Qualitative and quantitative assessment of the experimentally derived protein structures of CTD of AtWRKY4, and resolved through NMR(1WJ2) and with X-Ray diffracted crystal structure of AtWRKY4 complexed(2LEX) with our predicted models SolyWRKY33 (both NTD and CTD and SolyWRKY37). The good quality protein models the values must ranges in between 0–1.5 (RESPROX). The most favoured region in the predicted structural models was found to occuied 100% (SolyWRKY33 NTD) (PROCHECK) compared to template where number of residues in outlier and disallowed region covered significant residues.

S.No	Protein Name	Q Mean Score	Z Score	RESPROX	Most Favored (%)	Additionally allowed (%)	Outlier residues (%)
1.	N-terminal WRKY33 (NP_001306910.1) DNA binding domain (Predicted Model)	0.687	-2.61	1.91	100.0	0.0	0.0
2.	C-terminal WRKY33 (NP_001306910.1) DNA binding domain (Predicted Model)	0.537	-3.05	2.342	98.4	1.6	0.0
3.	WRKY 37 (NP_001308885.1) DNA binding Domain (Predicted Model)	0.533	-2.03	1.154	98.3	1.7	0.0
4.	C-terminal Domain of AtWRKY4 (PDB:1WJ2) (NMR determined)	0.557	-3.02	2.274	89.9	5.8	4.3
5.	C-terminal Domain of AtWRKY1 (PDB:2AYD) (X-Ray Diffraction)	0.811	-3.37	1.204	100	0.0	0.0
6.	Complex of C-terminal Domain of AtWRKY4and W- box DNA (PDB:2LEX)	0.470	-2.94	2.793	77.0	14.8	8.2

#### DNA-protein interaction

Molecular docking studies through Hex docking programme provided a computational approach to suggest and predict the possible modes of ligand (protein)-receptor (W-box DNA) binding. Docking allows predicting the best possible complex based on the lowest free energy values [96]. In case of the SolyWRKY33 we have analyzed the DNA- protein docking at both ends (both NTD and CTD). A total ten analogues each for SolyWRKY33 and SolyWRKY37 were docked and the efficacies of docked complexes were determined based on their interactive binding energy. The protein-DNA complex having least (most negative) values for the interaction energies (most stable complex) was selected.

**Fig 13:**
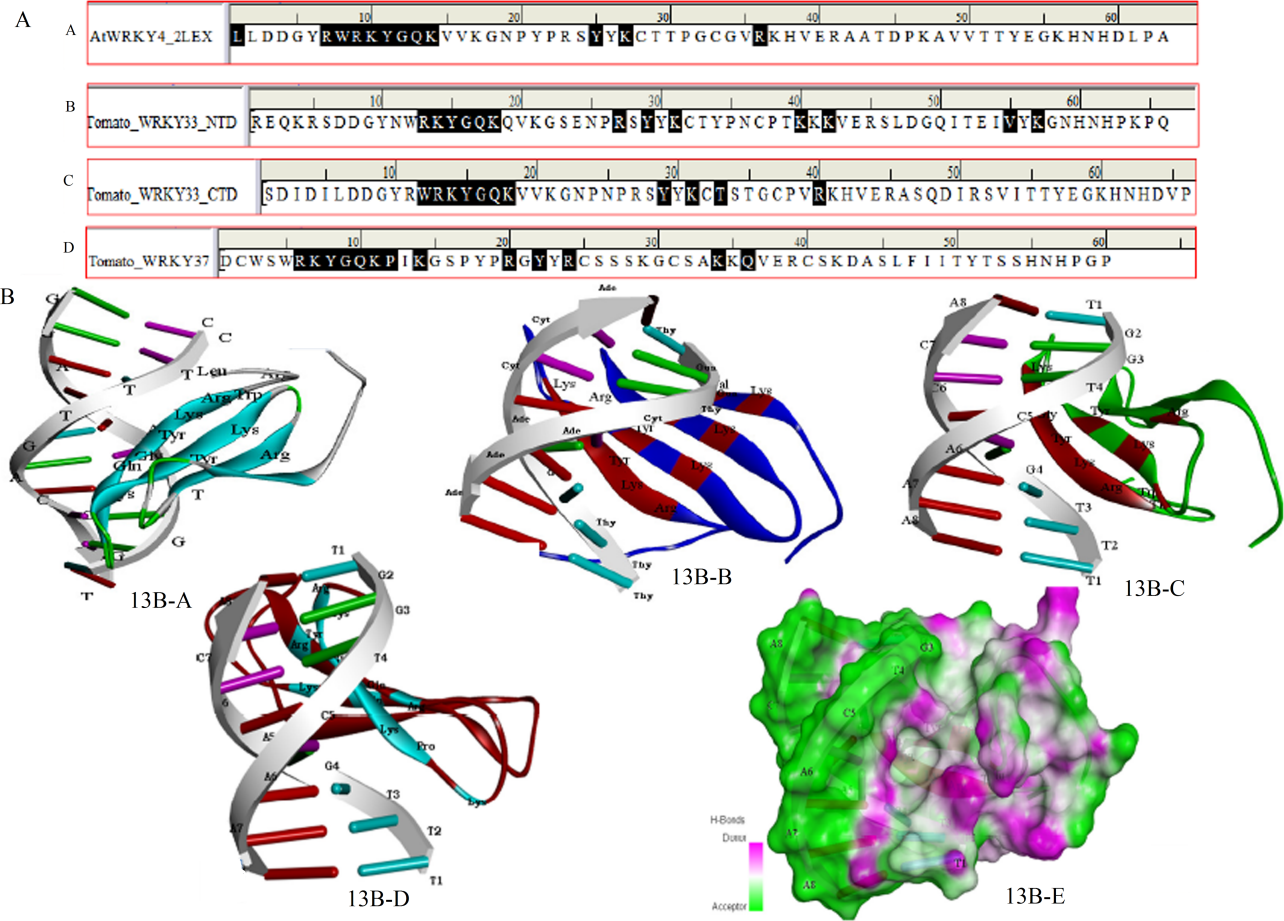
The sequential representation of the ligand interacting residues in the WRKY-DNA docked complexes. **13A-A.** Interaction of X-Ray determined crystal structure of docked complex of AtWRKY4 CTD with W-box DNA. **13A-B.** Docking of SolyWRKY33 NTD with W-box element **13A-C.** Docking of functional SolyWRKY33 CTD. **13A-D.** Docking of functional SolyWRKY37 domain with W-box DNA. **13 B.** Docking representations (pictorial) of molecular complexes of W-box DNA with functional WRKY domain. **13B-A.** Interaction of X-Ray determined crystal structure of docked complex of AtWRKY4 CTD with W-box DNA. **13B-B.** Docking of SolyWRKY33 NTD with W-box element. **13B-B.** Docking of functional SolyWRKY33 CTD. **13B-C.** Docking of functional SolyWRKY33 CTD with W-box DNA. **13B-D.** Docking of functional SolyWRKY37 domain with W-box DNA. **13B-E.** The three dimensional surface view for the WRKY- DNA interaction highlighting the docked regions in terms of H bond donar and acceptor groups in the docked complexes.

In our results, the docking energy scores for the SolyWRKY33 NTD-W-box DNA was found to be E_total =_ -1733.65KJ/M _._For the SolyWRKY33 CTD, the interaction energy of the most stable complex was found to be E_total_ = -1369.11KJ/M. Similarly, the interaction energy for the docked W-box DNA- SolyWRKY37 complex was reported to be E_total_ = **-**1539.32KJ/M. The most probable amino acid residues that were involved in this DNA protein interaction were highlighted (Fig 13A). The residues that made interaction with W-box DNA from CTD of AtWRKY4 (PDB ID: 2LEX) whose structure have been determined through NMR have been shown (Fig 13B-A). Docking studies revealed that the N terminal WRKY domain (NTD) binds through Arg^211^, Lys^212^, Tyr^213^, Gly^214^, Glu^215^, Lys^216^, Arg^225^, Tyr^227^, Lys^229^, Lys^238^, Lys^240^, Val ^253^ and Lys^255^ (Fig 13B-B) whereas the CTD of the SolyWRKY33 made interaction with the W- box core (TTTGACAA) sequences with the help of key residues including Trp^383^, Arg^384^, Lys^385^, Tyr^386^, Gly^387^, Lys^389^ (Fig 13B-C). The key residues involved with the docked SolyWRKY37 were Arg^59^, Lys ^60^, Tyr^61^, Gly^62^, Gln^63^, Lys^64^, Pro^65^, Arg^73^, Tyr^75^, Arg^77^, Lys^87^ and Gln^89^ (Fig 13B-D).

#### Functional annotation and protein subcellular localization

**Fig 14:**
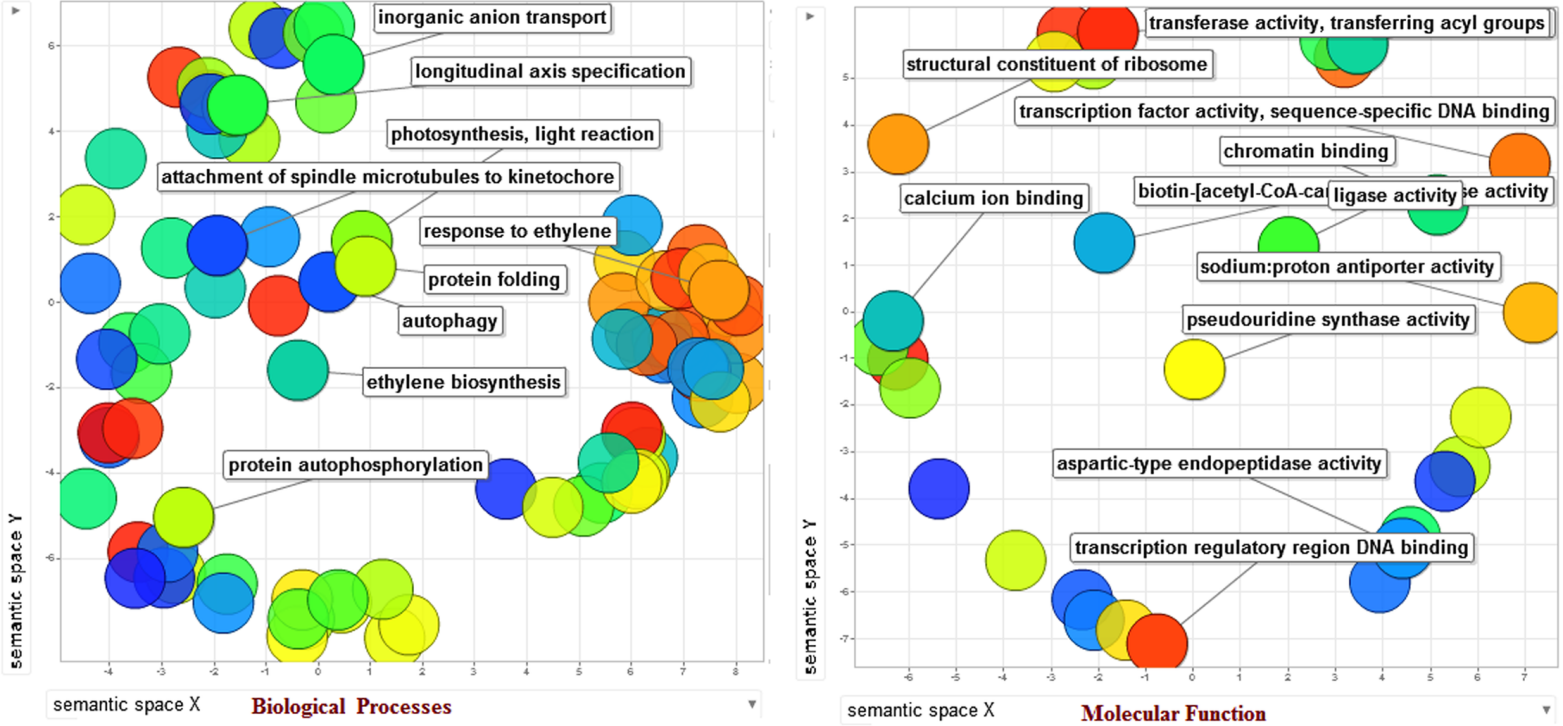
Scattered plot analysis for the redundant gene ontology (GO) terms based on the controlled functional vocabularies concentrated around the two ontological terms including biological process and molecular function. The first five significant terms were shown on scattered plots.

**Fig 15:**
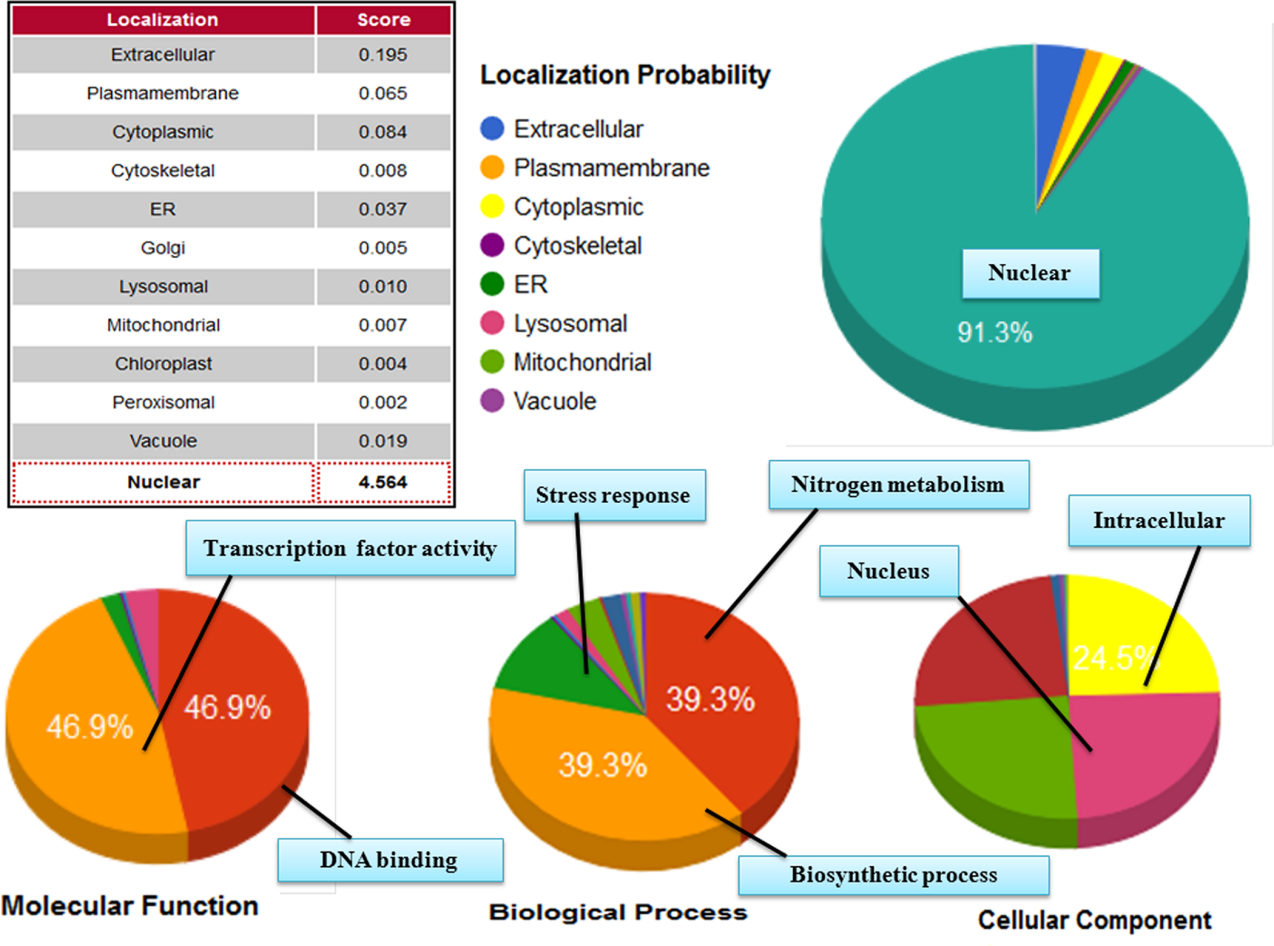
The subcellular localization of the predicted protein with functional gene annotation using CELLO2GO web server. The functional vocabularies’ are represented in pie chart diagrame evaluating the significant terms in form of their percentage contribution.

The protein sequences (both SolyWRKY33 and SolyWRKY37 were further characterized based on their structural and functional annotation using gene ontology (GO) enrichment analysis (S4 Table). Further, these enriched gene functional categories were interpreted using ReviGO analysis and the non-redundant GO terms were further analyzed through scattered plot diagramme (Fig 14). The GO IDs was followed using values where higher value is considered to be better for meaningful representation of GO terms. GO terms denote the molecular descriptors and represent the function products of gene and were found to be clustered around three ontologies including molecular function, sub-cellular component distribution and biological processes [97]. The functional annotation of the WRKY proteins was further explored through CELLO2GO in terms of GO enrichment details and protein subcellular localization (Fig 15).

The first five significant terms in biological processes revealed through gene enrichment ontology include transcription regulation (GO: 0006351), defense response (GO: 0006952), response to chitin (GO: 0010200), signal transduction (GO: 0007165), and respiratory burst involved in defense response (S4 Table). In addition to these other biological processes include cellular heat accumulation (GO: 0030370), longitudinal axis specification (GO: 0009942), cellular protein modification process (GO: 0006464) and toxin catabolic process (GO: 0009407). The molecular functions in terms of GO analysis were found to be the sequence-specific DNA binding transcription factor (GO: 0003700), sequence-specific DNA binding (GO: 0043565), ATP binding (GO: 004353), ADP binding (GO: 004353), which suggests their putative function in metabolic processes.

In cellular components, the most significant GO terms were found to be located inside the nucleus (GO: 0005634) which explain their sequence-specific DNA binding and regulation of transcription, cytoplasm (GO: 0005737) and membrane integral component (GO: 0016021). These functions indicate their crucial role in the plant developmental aspects, defense responses particularly against pathogen-induced challenges and other stressful conditions.

## Discussion

In the current study, we have characterized the WRKY transcripts that play the crucial role in tomato plant defense response against *Fol* challenged conditions using microarray analysis. The array results were further validated and confirmed through qRT-PCR analysis. The real-time results validated and matched the expression pattern of the microarray experiment for *SolyWRKY33* and *SolyWRKY37* with slight differences observed for *SolyWRKY4*. The qRT-PCR analysis showed that *SolyWRKY4* gene was downregulated compared to control samples against the microarray expression data, where it was slightly upregulated in array studies under *Fol* challenged conditions. Quantitative real-time PCR (qRT-PCR) is a commonly used validation tool for confirming gene expression results obtained from microarray analysis; however, microarray and qPCR data often result in disagreement [98]. It is well documented that both qPCR and microarray analysis have inherent pitfalls [99–103]. The correlation of gene expression results between the two methods is influenced by data quality parameters, that may significantly influence the data obtained from each method. The correlation between the two methods is affected by the direction of regulation and qRT-PCR C_t_ values and different normalization procedures [98]. Recently, Gujjar et al.[104] evaluated the expression pattern of eight drought -responsive genes (*SolyWRKY4*, *SlEFH-12*, *SlUSPA9*, *SlSNF4-15*, *SlPRP16*, *SlCYP51-17*, *SlMCP-119*, *SlGDSL-20*) in both drought tolerant and sensitive lines of wild tomato (*Solanum habrochaites*) under the artificially imposed drought conditions using real time PCR to validate the gene expression as performed through microarray analysis, and found that qRT-PCR results were almost similar to those of microarray experiment, with slight differences as found for *SolyWRKY4*. Real-time PCR analysis showed that *SolyWRKY4* gene was downregulated in both the lines, as against the microarray expression data, where it was reported to be slightly upregulated in sensitive line. The tissue specific gene expression using qRT-PCR analysis revealed the continuous and increased expression of *SolyWRKY33* and *SolyWRKY37* at an increased time interval (24–48 hr) in infected root tissues whereas the *wrky33* expression in leaf samples was first increased (7.36 fold) at 24 hrs then declined (3.48 fold). The expression of *SolyWRKY37* was found to be more or less similar (2.31 fold at 24 hrs) and 2.84 fold (48 hrs). In contrast, the expression of *SolyWRKY4* was found to be decreased in infected root tissues at 24 hrs with respect to un-inoculated control tissues (0 hrs). The increased expression of *SolyWRKY4* from 24–48 hrs (0.45–0.72 fold) in root tissues and (relative fold changes with respect to control) well explained the *SolyWRKY* gene mediated defense response. However, we did not get the similar response with *SolyWRKY4* in leaf tissues where a continuum decrease in *SolyWRKY4* expression was recorded. The inconsistency observed in *SolyWRKY4* gene expression with array result and qRT-PCR deduced experimental data is not clear. However, this might be attributed due to the hemibiotrophic mode of *Fol* pathogenesis because the *Fol* begins its infection cycle as a biotroph but later shift to necrotrophic mode of life cycle [105]. In the biotrophic phase, *F*. *oxysporum* establishes infection via the roots and travels towards the vasculature [105]. In *Arabidopsis* genetic overexpression and mutant analysis have suggested that *WRKY4* play a positive role in plant resistance to necrotrophic pathogens but a negative role in resistance to biotrophic pathogens [43]. The positive role of rice WRKY4 against *Rhizoctonia solani*, the rice sheath blight pathogen has been well demonstrated [93, 94]. Moreover, the positive role of WRKY33 in plant defense response against two necrotrophs *Alternaria brassicola* and *Botrytis cinerea* is well studied as *wrky33* mutants have been found to have enhanced susceptibility to these pathogens. Recently, the relative expression of the defense related *WRKY* genes in *Brassica rapa* against the two pathogens *F*. *oxysporum* f. sp. *conglutinans* and *Pectobacterium carotovorum* sub. sp. *carotovorum* have been demonstrated [97]. The highest expression of the *BrWRKY4* was recorded on the 6^th^ day post inoculation of the fungus *F*. *oxysporum* f. sp. *conglutinans*. In contrast, inoculation of *Pectobacterium carotovorum* sub. sp. *carotovorum* resulted into the fluctuations in the *WRKY* gene expression pattern with increased expression at 6^th^ hrs, found to be constant upto 3 days followed by a drastic increase in expression on the 7^th^ day of post inoculation [97]. We have first time reported the expression of *WRKY* genes following the attack of vascular wilt pathogen (*Fol*). Additionally, our results reported for the first one novel WRKY member WRKY37 in tomato whose role has been confirmed through both *insilico* and quantitative expression data in plant defense response against *Fol* challenged conditions.

### H_2_O_2_ generation, lignification and assessment of cell death

The early stage of the pathogenic inoculation is well regulated by the induction of systemic acquired resistance (SAR) at the infection sites, preventing the growth and dissemination of pathogens [19] and is mediated by SA signaling pathways. Moreover, the upregulation of SOD gene, along with a diminished scavenging system activity, resulted in a higher H_2_O_2_ burst. The SA related gene expression, when compared with the expression of the PR proteins followed by oxidative burst and the accumulation of H_2_O_2,_ revealed the higher H_2_O_2_ production along with the enhanced activities of PR proteins, required for imparting the resistance [19]. It has been reported that WRKY TFs play a crucial role in molecular crosstalk that occurred in between SA and JA signaling pathways. Furthermore, SA mediated suppression of JA signaling incorporates WRKY proteins along with other TFs [106, 107]. In this context, the WRKY TFs act as a node of convergence between SA and JA signaling [106]. In case of *Fol* infection, it has been reported that the induced resistance developed in tomato is mediated by SA dependent systemic acquired resistance [108]. However, Gimenez-Ibanez and Solano [107] described the negative crosstalk occurs in between SA and JA signaling pathways. Since *wrky33* mutant studies revealed the increased expression of several SA regulated genes (*EDS5/SID1*, *SID2/ICS1*, *NIMIN1*, *PAD4*, *EDS1 PR1*, *PR2*, and *PR3*) and higher accumulation of SA. Consecutively, induction of SA contributes to downregulate JA signaling cascades and to decrease the resistance of *wrky33* plants to necrotrophic fungi [109]. Moreover, the rapid pathogen-induced WRKY33 expression does not require SA signaling but is dependent on *PAD4*, a key regulator upstream of SA [110]. Loss of WRKY33 function results in inappropriate activation of the SA-related host response and elevated SA levels post infection and in the downregulation of JA-associated responses at later stages [111]. It was therefore, concluded that WRKY33, a positive regulator of JA-related genes, is a repressor of the SA pathway. The defense response of pea against the biotrophic pathogen *Erysiphe pisi* have been investigated [112] and it was reported that the higher accumulation of H_2_O_2_ in the pathogen challenged plant samples. In another study, the effect of pure metabolites from necrotrophic pathogen, *Alternaria alternata* on tomato plants was demonstrated [53] and the higher accumulation of H_2_O_2_ in samples, treated with pure metabolites have been reported [53]. The histochemical staining through the Evans blue dye revealed the assessment of the cell death in leaf tissues as indicated by the blue coloration occupied in between the unstained healthy control tissues. Evans blue is a non-permeating dye. In presence of plasma membrane damage, the dye enters in the cytoplasm and nucleus, thereby staining them blue [45]. Evans blue is used for checking cell viability. The cell death due to the exposed pure metabolites over leaf and stem tissues from the necrotrophic fungus *Alternaria alternata* has been well reported [53]. The higher amount of H_2_O_2_ generation also reflects the activation of the phenyl propanoid pathways that leads to the biosynthesis of lignin and other phenolic compounds [112]. In our results, the enhanced lignification was found in *Fol* challenged samples when compared to the control. During the *Fol* challenged conditions the wall modifications could lead into the deposition of additional layers, formation of various apopositions, and infusion with phenolic compounds or derived products. Moreover, the *Fol* challenged condition could results into occlusion of xylem vessels with brown gums, and browning and death of some xylem parenchyma cells [113]. The importance of various cell wall modifications and formation of intravascular wound periderm against vascular wilt causing fungus in carnations is reported [113]. The activation of the phenylpropanoid pathway, following the inoculation of a soil borne vascular wilt fungus, *Verticillium dahliae* that results into increased lignification in the stem in the resistant cultivars of cotton has been experimentally demonstrated through the histochemical analysis [114].

### WRKY domain analysis

It has been reported that the single domain containing WRKY proteins (group II and III) showed more sequence similarity to C-terminal domain (CTD) of the group I WRKY TFs rather than their N-terminal domain [115], which predicts the commensurate function of the group I WRKY proteins with the group II and III (shared the major DNA binding domain). The WRKY members having single domain are characterized by the presence of conserved W-R-K-Y-G-Q-K, P-R-x-Y-Y-x-C-x5-C, K-x-V, and H-x-H domains and conserved amino acid residues. In contrast, the members having two WRKY domains have conserved D-G-Y-N-W-R-K-Y-G-Q-K and R-S-Y-Y-x-C-x4-C-x22-H-x-H residues at their N-terminal end and conserved D-G-Y-R-W-R-K-Y-G-Q-K, R-S-Y-Y-x-C-x4-C, V-R-K-H-V-E, and H-x-H residues occupying at the C-terminal end [115]. Besides the conserved WRKYGQK region the spacing between the C and H residues in the zinc finger motif is unique, and the distinguishable features for other WRKY members.

### Motif composition analysis

The motif composition results revealed the distribution of uniform and similar motifs across the members when compared with the tomato WRKY33 and WRKY37 protein motifs. The statistically significant alignment score values are frequently assessed by their p-values. The frequency of the particular residue in motifs could be determined from their relative size whereas the positional information could be deduced from the height of the alphabetical letters. The phylogenetic analysis and the sequence alignment of WRKY domain (both N and C-terminal region) from the group I WRKY members in tomato, potato, and pepper disclosed the expansion of certain WRKY genes that might have occurred in the ancestor of above-mentioned members [116]. In contrast, NtWRKY33 were found to be more closely related with NaWRKY33 and might have been evolved from NbWRKY33 during the course of evolution. It has been suggested that the evolution of multigene families occurred due to the structural diversification of gene structure (exon/intron structure) [117] and the differences that exist in between the exon-intron structures and motif characteristics unravel the functional diversity of WRKY TFs. The microsyntenic relationship in between the tomato, *Arabidopsis*, and rice revealed the differences in between the orthologous gene pairs and established the close relationship of tomato WRKY group III proteins with *Arabidopsis*. Furthermore, the independent gene duplication and losses might have occurred during the evolution of species [26]. Moreover, the distribution of the motifs in NtWRKY33, NaWRKY33, and NbWRKY33 was found be to more interspersed, due to gene expansion and amplification that might have occurred due to the accumulation of synonymous and non-synonymous substitutions[118]. Interestingly, inspite of this gene expansion and amplification, *Nicotiana* WRKYs did not lead into the increase in a number of other WRKY members but terminated into the evolution of monocots [118]. However, we did not find any sequential homologs for WRKY37 protein sequences except with the members belonging to tomato family, which explain that the WRKY37 protein has been recently evolved in all the members and this could also be interpreted based on the motif distribution analysis. It was proposed that SolyWRKY37 could be classified with group II-e WRKY members (Solyc01g079360) and suggested the unique gene expansion event might have occurred for this group across the solanaceous crops [30]. However, the phylogenetic relationship and tree topology cluster SolyWRKY37 with SpWRKY37 and separate StWRKY37, NtWRKY37 and NaWRKY37.

In our results, we have established the phylogenetic relationship of the tomato WRKY33 and WRKY37 with their distantly related orthologs, to identify the similarities and differences occupied bythe members using circos visualization tools. The Blast-p results showed the sequential homologs available at database based on their percentage identity and query coverage values. Based on the results of phylogenetic relationship found that the SolyWRKY33 showed a homologous relationship with other members of Solanaceae and have an orthologous relationship with *Coffea canephora*, *Daucus carota*, *Prunus mume*, *Theobroma cacao*, based on total score, percentage query coverages, and identity. The phylogenetic studies were done on *A*. *thaliana*, *Solanum tuberosum*, *Oryza sativa* and *Nicotiana tabacum* have revealed that their *WRKY* genes are evolutionary more closely related to some *WRKY* genes in tomato. [119]. The close relationship between the *Arabidopsis* and tomato is well supported from the data of the comparative genomics as it was determined that these two species share the high number of orthologous genes (44.5%) and the other reorganization events [120]. Moreover, the similar motif composition shared by tomato WRKY with rice and *Arabidopsis* represent their close evolutionary relationship [39]. The orthologous genes present between tomato and *Arabidopsis* could be determined based on the extent of microcolinearity observed between tomato and *Arabidopsis* [121]. The data of comparative genomics have shown that most of the shared regions between tomato and *Arabidopsis* genome were considered to be Multiple Unique Matches(MUMs), and were found to be located the towards chromosomal ends. Moreover, the number of MUMs along the genome of two species closely mirrors the gene density in each species [122].

### Protein-protein functional interaction network

The STRING database results revealed the protein interacting partners for SolyWRKY33. It was reported that WRKY TFs could be regulated by other WRKY proteins with special binding to different *cis* elements [118]. The pathogen-associated defense-related genes are regulated by WRKY proteins binding with W or W-box (C/ T) TGAC (T/C) elements [123]. In plant immunity, WRKY TFs could form a complex interconnected regulatory network at several different levels [124,125]. The functional interactive protein network of tomato WRKY33 homologs for *Arabidopsis* showed the presence of common protein interacting partners in both tomato and *Arabidopsis*. Since, tomatoes share the highest evolutionarily conservation with *Arabidopsis* [126] this comparative and interlogs based functional network predicts the signaling network and most probable partners that are involved in tomato WRKY33 signaling cascades. Furthermore, the close phylogenetic relationship between tomato and *Arabidopsis* genes is well supported, and exemplified by the fact that both species partially employ similar proteins, for their developmental programming such as epidermal cell differentiation, development of root hairs, initiation of trichomes and accumulation of anthocyanins [127–129]. Recently, the interaction network for SolyWRKY group III TFs following the infection of tomato-leaf curl virus (TYLCV) infection was explored [117]. It was found that SolyWRKY group III proteins (SolyWRKY17, SolyWRKY41, SolyWRKY53, SolyWRKY54, and SolyWRKY80) showed interactions with other proteins in the tomato genome. The sequence alignment made between the tomato homologs for *Arabidopsis* WRKY33 and AtWRKY33 was shown to have extensive sequence similarity across the entire protein region including the extended CTDs [130] which revealed their similar function. The two identified genes reported in tomato were found to be highly homologous to *Arabidopsis* WRKY33 transcription factor [131]. Moreover, the gene silencing and molecular complementation studies revealed that tomato WRKY33 play a critical role in mitigating the stress response similar to AtWRKY33 [130]. The functional interactome studies established developed through PTIR could be employed as repositories for predicting the possible interactome map of tomato or other species on a genome wide scale [126] as it was suggested that the proteins having conserved functions could be used for interactome studies because the conserved proteins have same functions across the evolutionary related species and therefore, this functional network could be employed or transferred to other relevant species having orthologous relationship with previous partners [132]. Interestingly, the functional analysis of two autophagy-related (annotated) proteins (ATG5 and ATG7) from tomato genomes were searched for their homologs in model *Arabidopsis* and analyzed for their functional role in *Arabidopsis* [133–135].The protein-protein interaction data available from the model plant (*Arabidopsis thaliana)* was used to predict all the possible interactive partners (interactome analysis) in *Brassica rapa* [136]. Apart from other interactive partners, the SolyWRKY33 formed the interactive network with WRKY1, WRKY40, and WRKY70 which confirms their cooperative function in plant defense mediated signaling (S5 Table). One of the most important conclusions that was made through such *insilico* interaction studies that one can functionally analyze the role of the SolyWRKY33 in plant stress responses through the genetic complementation of the *Arabidopsis* WRKY33 mutants (*atwrky33*). These studies may have some positive outlook towards tomato breeding programme against various fungal diseases.

### DNA-protein interaction and gene prediction

The stability of the docked complexes was analyzed from several backgrounds and the parameter that provided the complexes with having minimum electro potential energy was further optimized for docking calculations. For example the DNA- protein docking using correlation shape only gave all the docked complexes with higher calculated energy score values (SolyWRKY33 NTD E_**total**_ = -522.37 KJ/mol and E_**shape**_ = -544.59 KJ/mol; SolyWRKY33 CTD E_**shape**_ = **-**544.59 KJ/mol and E_**shape**_ = -544.59 KJ/mol and lastly for the SolyWRKY37 E_**total**_ = -585.83 KJ/mol and E_**shape**_ = **-**585.83 KJ/mol. Similarly, the other parameters selected Shape + Electrostat gave the high energy score values, for all the docked complexes forming unstable complexes, and therefore, get avoided. In contrast, when DARS (Decoys As the Reference State) potential were chosen with other energy terms (e.g., van der Waals and electrostatics) (Shape + Electro + DARS) we got all the docked complexes with least energy values, and therefore, the stable complexes with strong docking ability have been shown in our results. Our results were found to be in compliance with the experiments where DARS mode has been predicted as “best mode” for construction of structure-based intermolecular potentials [76].

We have compared our docking results with the NMR generated structure of AtWRKY4 CTD complexed with W-box DNA(PDB ID: 2 LEX) **(**Fig 13A) and found that the residues Lys, Arg, Trp, Tyr, Gly, and Gln are the key players that took part in interacting the *cis* sequences of the W-box. It was proposed that in AtWRKY4-DNA complex about eight bases in 7 consecutive base pairs were found to be interacted by Arg^415^, Lys^416^, Tyr^417^, Gly^418^, Gln^419^, and Lys^420^ of the β_1_-strand or all of the residues in the invariant WRKYGQK sequence, except Trp^414^, through apolar and hydrogen-bonding interactions [137]. The major molecular interface was composed by the β1 strand containing the invariant WRKYGQK sequence. We have shown the 3D molecular interface for DNA-protein complex showing the major H bond donar and acceptor (Fig 13B–13E). It was suggested that the C-terminal WRKY domain play the crucial role in DNA binding whereas, the N- terminal region favours protein-protein interaction [138]. Interstingly, the DNA-protein interaction studies conducted in this way have shown the importance of polar (positive and negatively charged) amino acids that makes H-bonds with negatively charged backbone DNA therefore, play a very critical role in making the interaction more feasible for effective gene regulation. Moreover, the preferential binding shown by our results for group I (SolyWRKY33) and group II-e WRKY TFs (SolyWRKY37) is well supported by the computationally predicted as well as experimentally determined solution structure of DNA-protein complex of three different WRKY group transcription factors, in *Arabidopsis* including AtWRKY33 (group I), AtWRKY11 (group IId) and AtWRKY50 (group IIc) that predict the differences and specificities for their DNA binding, and suggested the mechanism through which the differential variability is influenced and achieved [139]. Furthermore, through computational assessment of this DNA-protein interaction we have highlighted the relevance of positively and negatively charged amino acid in forming the stable complexes [in 3D surface diagrame] in highly specific and multispecific DNA protein interactions [140]. The β_1_ strand having WRKYGQK sequence bind specifically to the W- Box DNA due to the highly specific and multispecific binding preferably shown by aromatic amino acid residues particularly, Tryptophan (W), having special affinities for the cytosine and guanine bases of DNA [141]. TFs have a high preference for specific propeller twist angles surrounding their binding sites [142]. The inconsistencies observed in the experimental results well suggested the need for a rather stringent conformational structure for high affinity binding and further in depth studies for the structural determination of the protein complexed with DNA and sequence specific DNA recognition by WRKY domains. However, it has been reported that WRKY TFs can bind to other functional *cis*-acting elements [143]. The PLACE server revealed the core elements that could be involved in binding with WRKY TFs including the TGACT(WBOXHVISO1; SURE (sugar responsive) is a *cis* element in plant sugar signaling, TTGAC (WBOXATNPR1; salicylic acid induced WRKY DNA binding proteins [144] TGAC (WRKY71OS; that constitutes the defense signaling of WRKY proteins and found their role in pathogenesis. Moreover, the results indicate that the selected candidate *WRKY* gene involved in transcription repression of gibberellic acid signaling pathway [145], TGACY (WBOXNTERF3) "W box" located inside the gene *ERF3* that constitutes the promoter region and activates the *ERF3* genes following the wound response [146]. The other *cis* acting elements ACGT (ABRE like motif), TAACTG (MYB core element) and MYB related motifs (CNGTTR, YAACKG, GGATG) [147] that mediates the regulation of genes involved in water stress in *Arabidopsis* [144]. Similarly, other motifs such as CANNTG (Myc motif also called as R response element) and CATGTG (Myc related motifs) have been also reported for light-responsive and tissue-specific activation of phenylpropanoid biosynthesis genes [148]. The list of all identified *cis*-acting elements found within the promoter region of WRKY protein was reported in (S6 Table). The presence of these *cis*-acting elements in upstream promoter region provide sufficient evidences for the role of candidate WRKY in plant defense response against both biotic and abiotic stress responses.

## Conclusion

The present study discussed the structural attributes and the functional role and regulation of defense -related and differentially expressed (upregulated) WRKY transcripts under the biotic stress conditions. The expression profile from the tomato microarray datasets uncovered the importance of identified *WRKY* genes in the plant defense response, under *Fol* challenged conditions. The structural and functional characterization of individual WRKY member through computational approaches, revealed the sequence-specific gene regulation, functional dimension, subcellular localization and the other possible biological processes in which their role have been reported. Due to relatively large size and sequential complexity observed in these transcription factors, it is desirable to compile the gene expression studies for different WRKYs, following the stress conditions, and to correlate with the datasets generated with members, for the better understanding of their evolutionary origin, molecular aspects, genetic analysis and the functional differentiation the gene family in orthologs. Genetic transformation strategies have been optimized and were exploited for raising stable transgenic plants for different abiotic and biotic factors. The *WRKY* gene mutants, defective for individual *WRKY* genes, isolated from-DNA-transformed or transposon-tagged populations, could be analyzed for investigating the most probable and possible alterations in plant growth, development and disease resistance. Moreover, if these mutants were analyzed with different WRKY proteins, could reveal the in-depth investigation ofthe defense signaling network orchestrated by plants in order to escape from stress -induced damages.

## Supporting information

S1 Fig Heat map showing the differential expression of WRKY genes under *Fol* challenged conditions. The three WRKY genes *SolyWRKY4*, *SolyWRKY33* and *SolyWRKY37* have been found to be upregulated among all the *Fol* challenged samples (red colour). The control or un-inoculated samples show the downregulation of genes (green colour). The data were retrieved from expression average values and analyzed through BiGGESTS software.
(TIF)

S2 Fig **A.** The phylogenetic tree showing the evolutionary origin and ancestral relationship with other sequential homologs and orthologs based on percent identity and query coverages with SolyWRKY33. The tree is constructed using maximum parsimonious method and the topological stability of the tree was evaluated with 1000 bootstrapping replications **S2 B**. Motif distribution analysis using MEME suite programme for finding the statistically significant motifs. The blue square indicate the significant motifs that constitute the full length WRKY domain and were found to uniform and conserved in different homologs of SolyWRKY33 and composed of WRKYGQKQVK (forming β1) strand), NPRSYYKCTY (forming β2 strand), CPTKKKVER (forming β3; dominated by lysine substitutions) and lastly VITTTYE motif (forming β4). **S2 C**. The sequential logo for motif showing the highly conserved WRKYGQK sequences from C-terminal WRKY domain. The relative sizes of the letters indicates their frequency in the sequences whereas the total height of the letters depicts the information content of the position, in bits of information.
(TIF)

S3 Fig **A.** The phylogenetic tree showing the evolutionary origin and ancestral relationship with other sequential homologs based on percent identity and query coverages with SolyWRKY37. The tree is constructed using maximum parsimonious method and the topological stability of the tree was evaluated with 1000 bootstrapping replications. **S3 B**.The motif distribution diagrame for SolyWRKY37 showed the presence of uniform motifs across the entire protein sequence and present among all the members with statistically significant p-values **S3 C.** Sequential logo diagrame showing the motif containing WRKYGQK sequences.
(TIF)

S4 Fig Multiple sequence alignment of the highly conserved WRKY domain (60 amino acids) from all the sequential homolog and orthologs for SolyWRKY33 and SolyWRKY37. **S4 A.** N-terminal end WRKY33 domain **S4 B.** C-terminal end WRKY33showing all the conserved four beta strands including WRKY domain and **S4 C.** WRKY domain region for SolyWRKY37. The red highlighted square indicates the strong conservation of the residues that constitutes the WRKY domain.
(TIF)

S5 Fig The presence of two WRKY domains in SolyWRKY33 as revealed by ExPASy-Prosite tool. The functional signature sequences at both the N-terminal and C-terminal end have been highlighted.
(TIF)

S6 Fig The presence of only one WRKY domains in SolyWRKY37 retrieved through \ ExPASy-Prosite tool. (TIF)

S7 Fig **A.** Acknowledgement details of the submitted protein models of SolylWRKY33 at PMDB database with their PMDB IDs author details, methods employed and reliability score values. **S7 B.** Details of the submitted protein models of SolyWRKY37.
(JPG)

S8 Fig Ramachandran plot statistics as revealed through RAMPAGE server revealing the displays the psi (*ψ*) and phi (*φ*) backbone conformational angles for each residue in a protein. (TIF)

S9 Fig Qualitative assessment of the modelled protein through Q mean server based on geometrical analysis of single models and the clustering based scoring function. The raw scores, Z-scores of the QMEAN composite score as well as all terms are provided relating the quality estimates to scores obtained for high-resolution reference structures solved experimentally by X-ray crystallography.
(TIF)

S10 Fig **A.** Qualitative assessment of the modelled protein based on the pattern of non-bonded atomic interactions. The ERRAT score values for predicted A. SolyWRKY33 NTD **S10 B.** SolyWRKY33 CTD **S10 C.** and SolyWRKY37 CTD. Error values are plotted as a function of the position of a sliding 9-residue window.
(TIF)

S1 Table GEO datasets representing the list of genome wide transcriptionally active genes having differential expression in control and pathogen treated samples with their transcript identities. (XLS)

S2 Table List of transcriptionally active genes showing upregulated expression in control samples and downregulated expression in pathogen treated samples. (XLSX)

S3 Table List of transcriptionally active genes showing downregulated expression in control samples and upregulated expression in pathogen treated samples. (XLSX)

S4 Table Gene ontology enrichment analysis summarizing the functional annotation from all the controlled vocabularies including biological process, molecular function, and cellular processes. The redundant GO terms have been displayed in the form of scattered plot values and all the functional annotations with the Gene ontology IDs have been shown in table along with their frequency and other values.
(DOCX)

S5 Table Protein-Protein functional interactive association: Functional annotation, accession ID and interacting score values for both first and second shell of interactors that form mutual interactive associative network along with SolyWRKY3. The higher value of score indicate the more frequent interaction exist between two associated proteins.
(XLS)

S6 Table *In silico* cis acting DNA regulatory element analysis for searching the promoters located upstream regions from transcriptional start site and may be employed by WRKY TFs in case of specialized signaling cascades. (XLS)
