# Supplementary figures and images for: Structural and functional dissection of differentially expressed tomato WRKY transcripts in host defense response against the vascular wilt pathogen (Fusarium oxysporum f. sp. lycopersici)
Source: PLoS One. 2018 Apr 30;13(4):e0193922. doi: 10.1371/journal.pone.0193922 (PMC5927432; doi:10.1371/journal.pone.0193922)

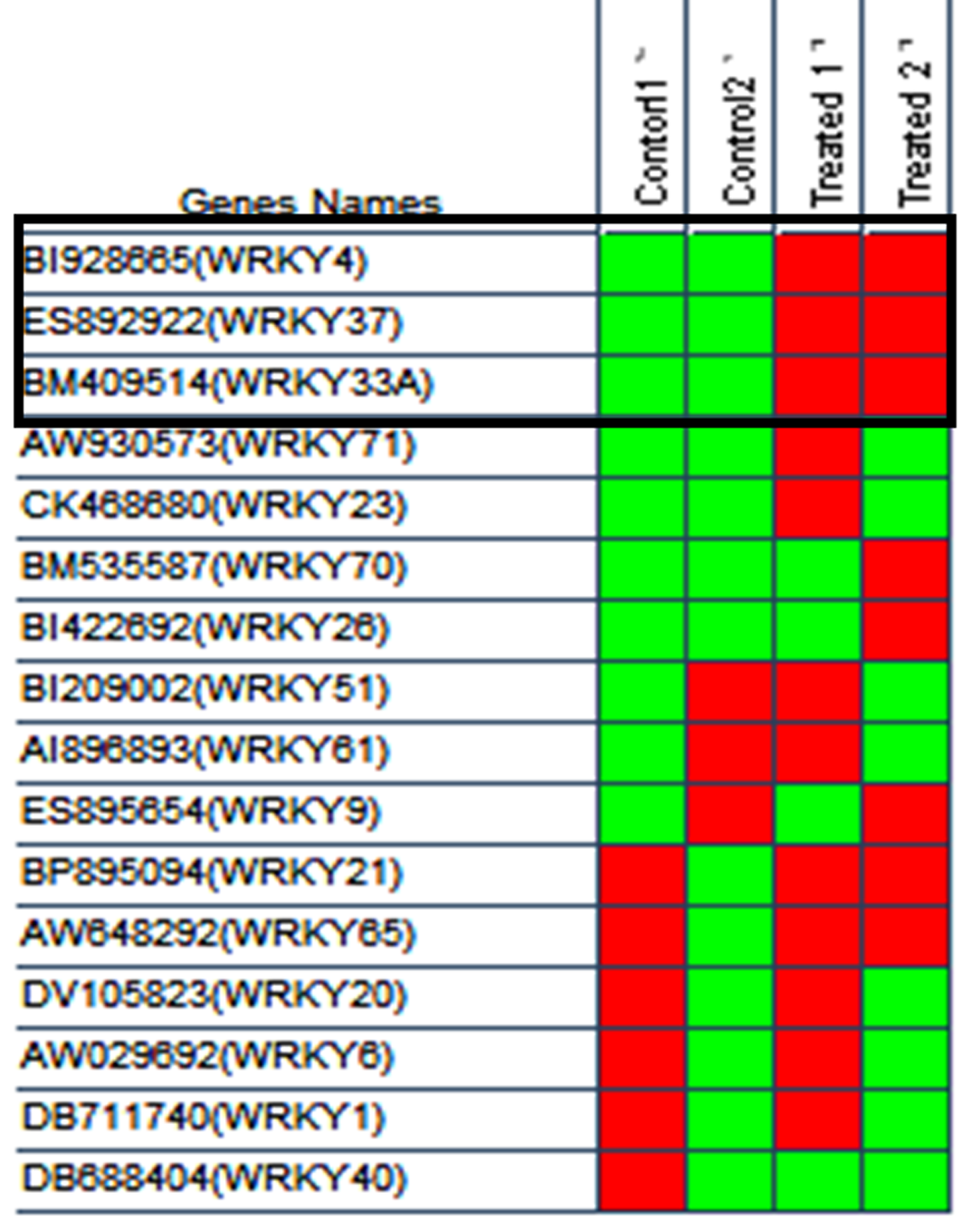

Supplement: S1 Fig — The three WRKY genes SolyWRKY4, SolyWRKY33 and SolyWRKY37 have been found to be upregulated among all the Fol challenged samples (red colour). The control or un-inoculated samples show the downregulation of genes (green colour). The data were retrieved from expression average values and analyzed through BiGGESTS software. (TIF) [file pone.0193922.s001.tif]

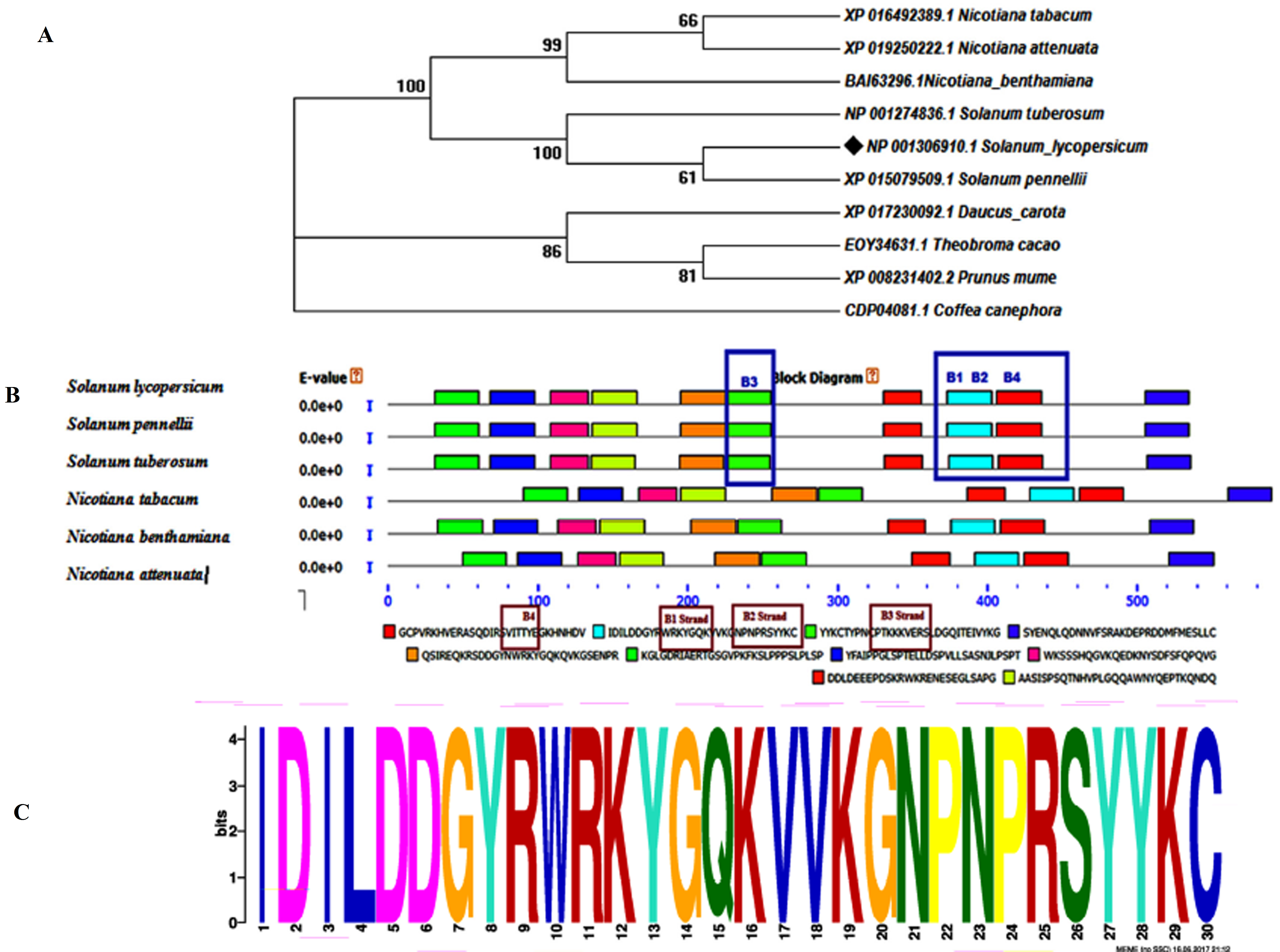

Supplement: S2 Fig — A. The phylogenetic tree showing the evolutionary origin and ancestral relationship with other sequential homologs and orthologs based on percent identity and query coverages with SolyWRKY33. The tree is constructed using maximum parsimonious method and the topological stability of the tree was evaluated with 1000 bootstrapping replications S2 B. Motif distribution analysis using MEME suite programme for finding the statistically significant motifs. The blue square indicate the significant motifs that constitute the full length WRKY domain and were found to uniform and conserved in different homologs of SolyWRKY33 and composed of WRKYGQKQVK (forming β1) strand), NPRSYYKCTY (forming β2 strand), CPTKKKVER (forming β3; dominated by lysine substitutions) and lastly VITTTYE motif (forming β4). S2 C. The sequential logo for motif showing the highly conserved WRKYGQK sequences from C-terminal WRKY domain. The relative sizes of the letters indicates their frequency in the sequences whereas the total height of the letters depicts the information content of the position, in bits of information. (TIF) [file pone.0193922.s002.tif]

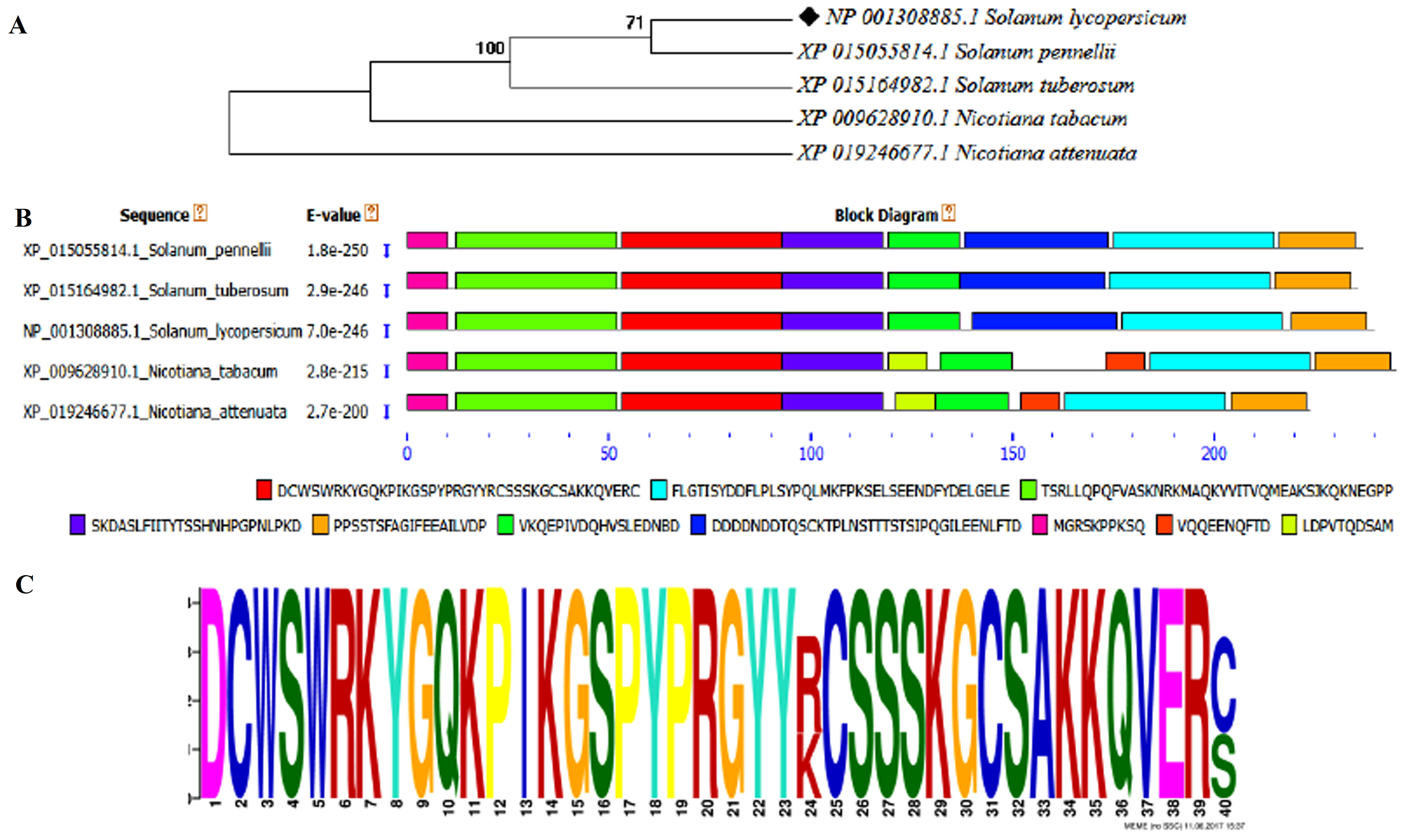

Supplement: S3 Fig — A. The phylogenetic tree showing the evolutionary origin and ancestral relationship with other sequential homologs based on percent identity and query coverages with SolyWRKY37. The tree is constructed using maximum parsimonious method and the topological stability of the tree was evaluated with 1000 bootstrapping replications. S3 B.The motif distribution diagrame for SolyWRKY37 showed the presence of uniform motifs across the entire protein sequence and present among all the members with statistically significant p-values S3 C. Sequential logo diagrame showing the motif containing WRKYGQK sequences. (TIF) [file pone.0193922.s003.tif]

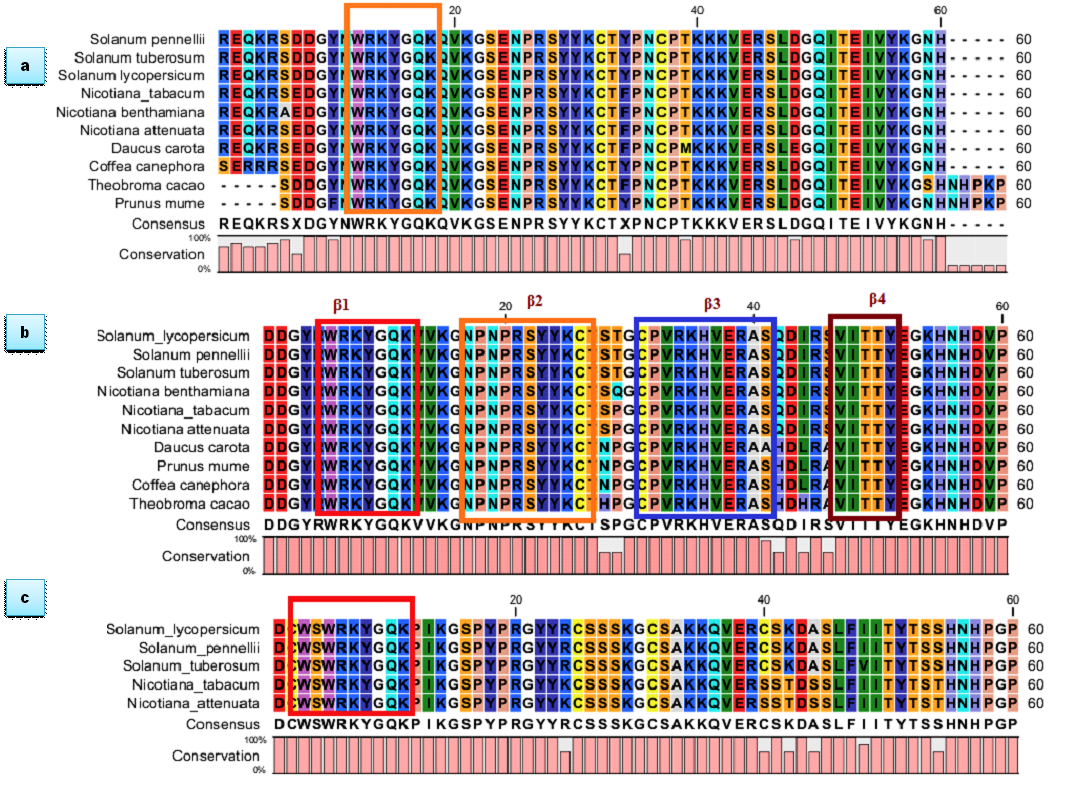

Supplement: S4 Fig — S4 A. N-terminal end WRKY33 domain S4 B. C-terminal end WRKY33showing all the conserved four beta strands including WRKY domain and S4 C. WRKY domain region for SolyWRKY37. The red highlighted square indicates the strong conservation of the residues that constitutes the WRKY domain. (TIF) [file pone.0193922.s004.tif]

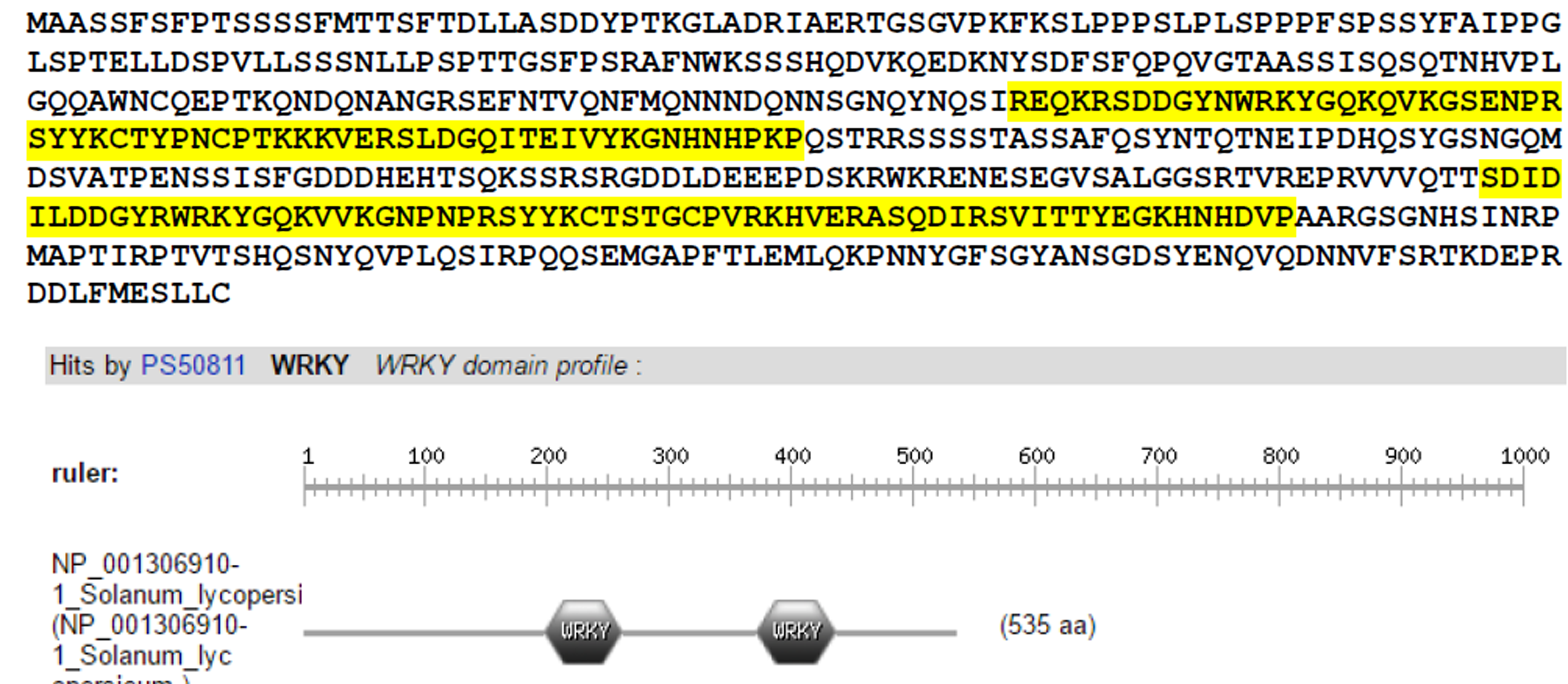

Supplement: S5 Fig — The functional signature sequences at both the N-terminal and C-terminal end have been highlighted. (TIF) [file pone.0193922.s005.tif]

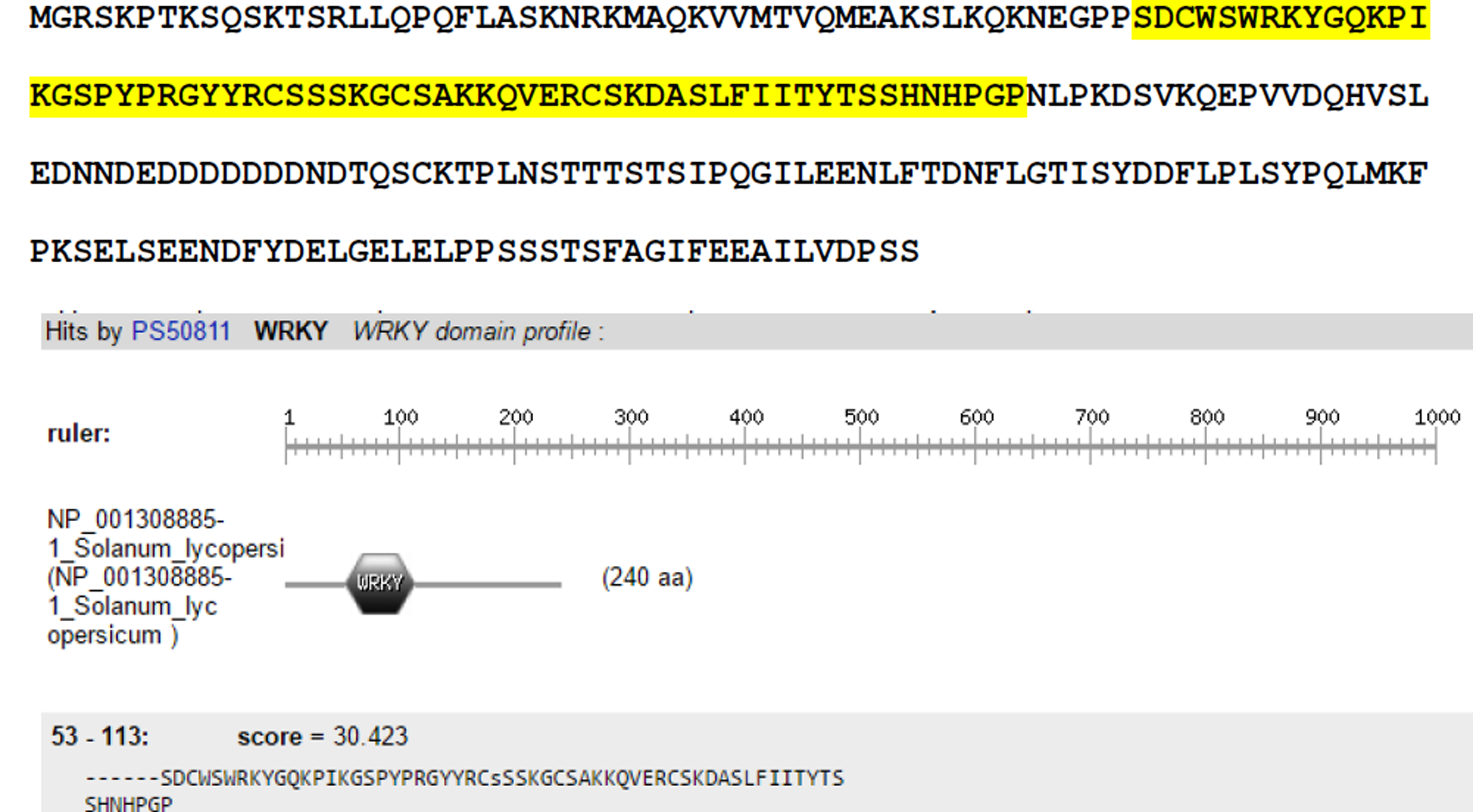

Supplement: S6 Fig — (TIF) [file pone.0193922.s006.tif]

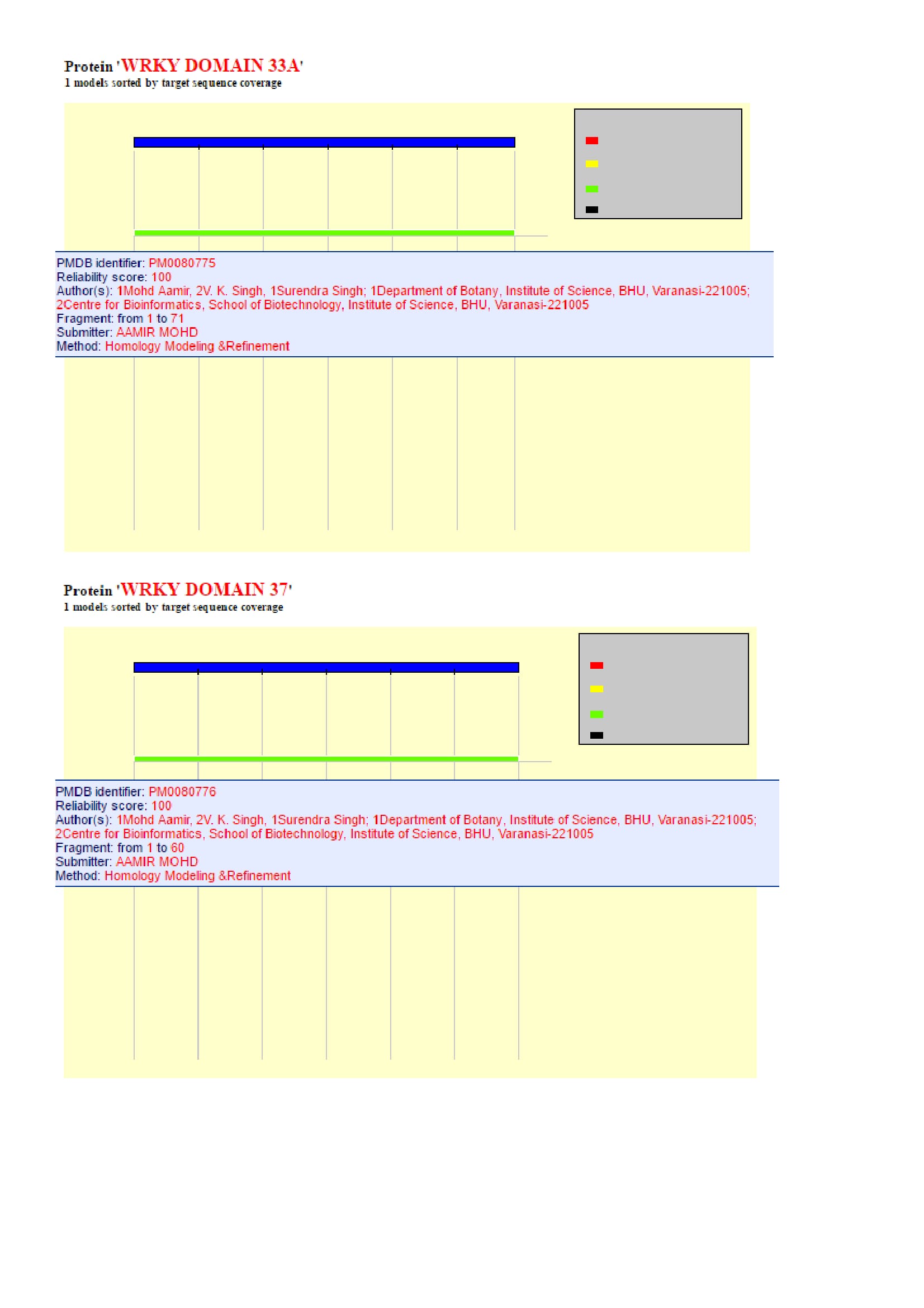

Supplement: S7 Fig — A. Acknowledgement details of the submitted protein models of SolylWRKY33 at PMDB database with their PMDB IDs author details, methods employed and reliability score values. S7 B. Details of the submitted protein models of SolyWRKY37. (JPG) [file pone.0193922.s007.jpg]

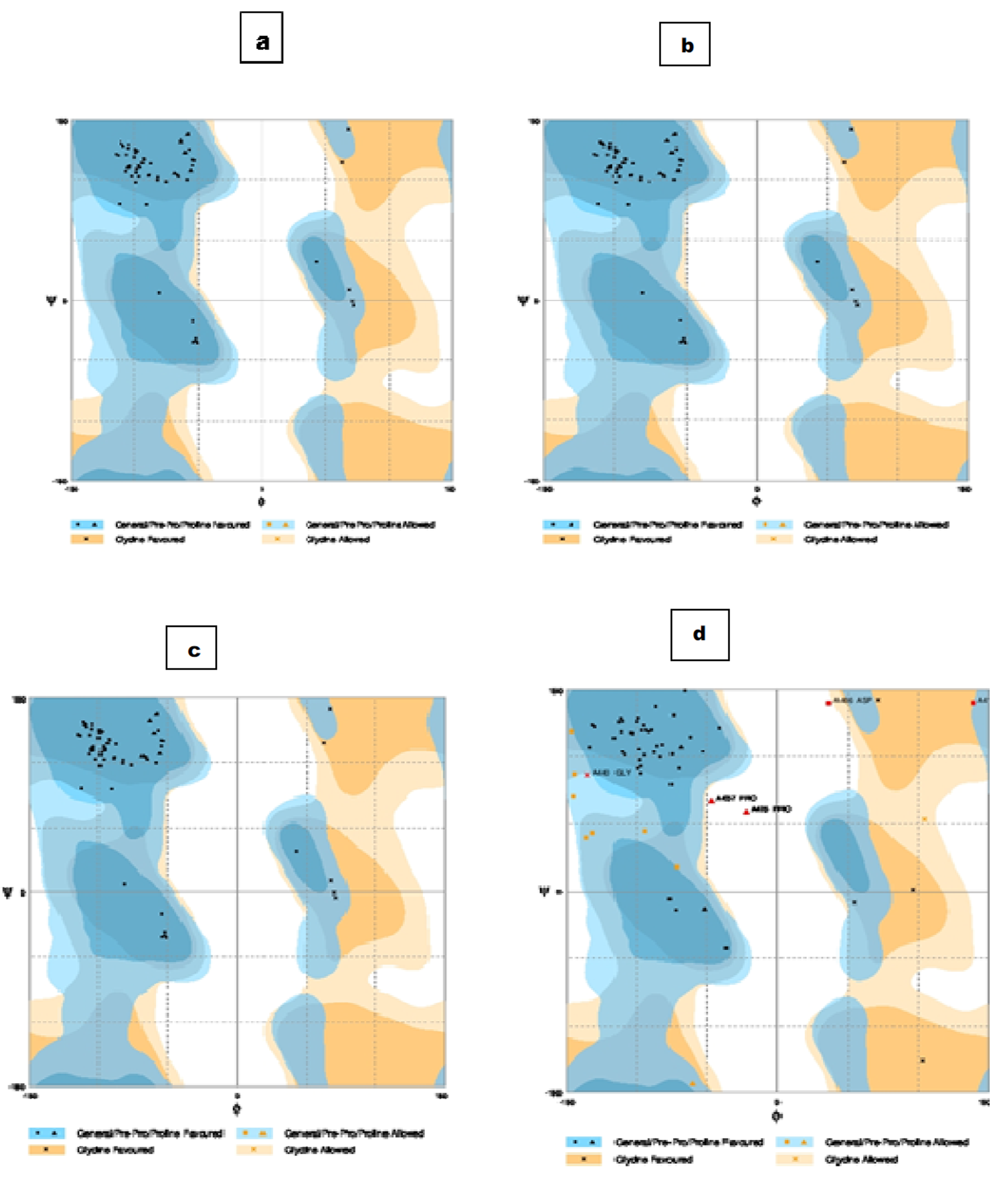

Supplement: S8 Fig — (TIF) [file pone.0193922.s008.tif]

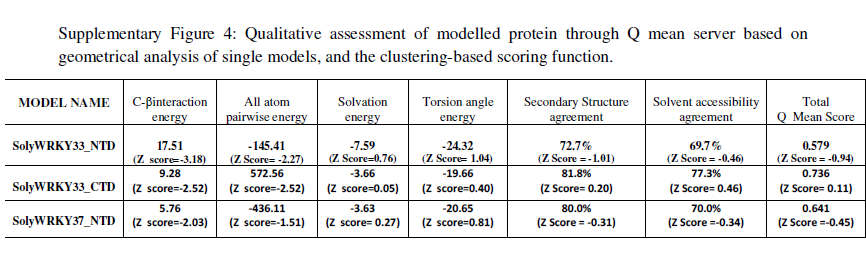

Supplement: S9 Fig — The raw scores, Z-scores of the QMEAN composite score as well as all terms are provided relating the quality estimates to scores obtained for high-resolution reference structures solved experimentally by X-ray crystallography. (TIF) [file pone.0193922.s009.tif]

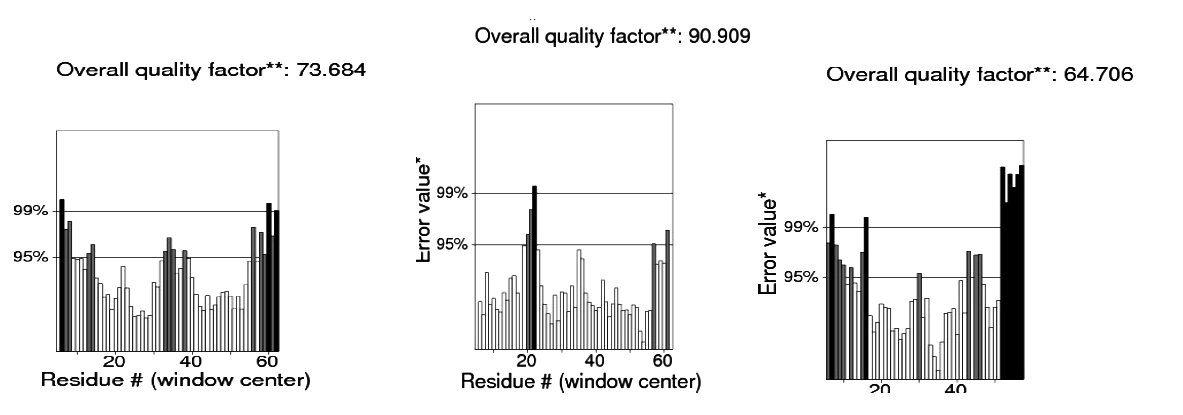

Supplement: S10 Fig — A. Qualitative assessment of the modelled protein based on the pattern of non-bonded atomic interactions. The ERRAT score values for predicted A. SolyWRKY33 NTD S10 B. SolyWRKY33 CTD S10 C. and SolyWRKY37 CTD. Error values are plotted as a function of the position of a sliding 9-residue window. (TIF) [file pone.0193922.s010.tif]
