# Supplementary material for: Structural and functional dissection of differentially expressed tomato WRKY transcripts in host defense response against the vascular wilt pathogen (Fusarium oxysporum f. sp. lycopersici)
Source: PLoS One. 2018 Apr 30;13(4):e0193922. doi: 10.1371/journal.pone.0193922 (PMC5927432; doi:10.1371/journal.pone.0193922)
Supplement: S4 Table — The redundant GO terms have been displayed in the form of scattered plot values and all the functional annotations with the Gene ontology IDs have been shown in table along with their frequency and other values. (DOCX) [file pone.0193922.s014.docx]

| **Node1**  Table: Protein-Protein functional interactive association: List of all the possible interactors from Node 1 and Node2 with their functional annotation, interacting score values and accession number in the tomato homologue for Arabidopsis (*Solanum lycopersicum*) | **Node2** | **Node1 accession** | **Node2 accession** | **Node1 annotation** | **Node2 annotation** | | **Score** |  |
| --- | --- | --- | --- | --- | --- | --- | --- | --- |
| CNI1 | CZF1 | AT5G27420.1 | AT2G40140.1 | Carbon/nitrogen insensitive 1; E3 ubiquitin-protein ligase that is required for the plant C/N response during seedling growth transition. May be involved in the early steps of the plant defense signaling pathway | Zinc finger CCCH domain-containing protein 29; Involved in salt stress response. May positively modulate plant tolerance to salt stress | | 0.937 |  |
| CNI1 | ERF104 | AT5G27420.1 | AT5G61600.1 | Carbon/nitrogen insensitive 1; E3 ubiquitin-protein ligase that is required for the plant C/N response during seedling growth transition. May be involved in the early steps of the plant defense signaling pathway | Ethylene response factor 104; Probably acts as a transcriptional activator. Binds to the GCC-box pathogenesis-related promoter element. May be involved in the regulation of gene expression by stress factors and by components of stress signal transduction pathways (By similarity) | | 0.614 |  |
| CNI1 | MKS1 | AT5G27420.1 | AT3G18690.1 | Carbon/nitrogen insensitive 1; E3 ubiquitin-protein ligase that is required for the plant C/N response during seedling growth transition. May be involved in the early steps of the plant defense signaling pathway | Protein MKS1; Regulator of plant defense response. May contribute to MPK4-regulated defense activation by coupling the kinase to specific WRKY transcription factors | | 0.560 |  |
| CNI1 | MPK3 | AT5G27420.1 | AT3G45640.1 | carbon/nitrogen insensitive 1; E3 ubiquitin-protein ligase that is required for the plant C/N response during seedling growth transition. May be involved in the early steps of the plant defense signaling pathway | Mitogen-activated protein kinase 3; Involved in oxidative stress-mediated signaling cascade to oxidative (such as ozone). Involved in the innate immune MAP kinase signaling cascade (MEKK1, MKK4/MKK5 and MPK3/MPK6) downstream of bacterial flagellin receptor FLS2. May be involved in hypersensitive response (HR)-mediated signaling cascade. May phosphorylate regulators of WRKY transcription factors. Mediates the phosphorylation of VIP1 and subsequent stress genes transcription in response to Agrobacterium | | 0.698 |  |
| CNI1 | STZ | AT5G27420.1 | AT1G27730.1 | Carbon/nitrogen insensitive 1; E3 ubiquitin-protein ligase that is required for the plant C/N response during seedling growth transition. May be involved in the early steps of the plant defense signaling pathway | salt tolerance zinc finger; Transcriptional repressor involved in abiotic stress responses. Can repress the stress responsive genes DREB1A and LTI78. Probably involved in jasmonate (JA) early signaling response. May regulate the expression of the JA biosynthesis gene LOX3 and control the expression of TIFY10A/JAZ1, a key repressor in the JA signaling cascade | | 0.896 |  |
| CNI1 | WRKY33 | AT5G27420.1 | AT2G38470.1 | Carbon/nitrogen insensitive 1; E3 ubiquitin-protein ligase that is required for the plant C/N response during seedling growth transition. May be involved in the early steps of the plant defense signaling pathway | WRKY DNA-binding protein 33; Transcription factor. Interacts specifically with the W box (5’-(T)TGAC[CT]-3’), a frequently occurring elicitor- responsive cis-acting element (By similarity) | | 0.954 |  |
| CNI1 | WRKY40 | AT5G27420.1 | AT1G80840.1 | Carbon/nitrogen insensitive 1; E3 ubiquitin-protein ligase that is required for the plant C/N response during seedling growth transition. May be involved in the early steps of the plant defense signaling pathway | WRKY DNA-binding protein 40; Transcription factor. Interacts specifically with the W box (5’-(T)TGAC[CT]-3’), a frequently occurring elicitor- responsive cis-acting element (By similarity) | | 0.839 |  |
| CZF1 | CNI1 | AT2G40140.1 | AT5G27420.1 | Zinc finger CCCH domain-containing protein 29; Involved in salt stress response. May positively modulate plant tolerance to salt stress | Carbon/nitrogen insensitive 1; E3 ubiquitin-protein ligase that is required for the plant C/N response during seedling growth transition. May be involved in the early steps of the plant defense signaling pathway | | 0.937 |  |
| CZF1 | ERF104 | AT2G40140.1 | AT5G61600.1 | Zinc finger CCCH domain-containing protein 29; Involved in salt stress response. May positively modulate plant tolerance to salt stress | Ethylene response factor 104; Probably acts as a transcriptional activator. Binds to the GCC-box pathogenesis-related promoter element. May be involved in the regulation of gene expression by stress factors and by components of stress signal transduction pathways (By similarity) | | 0.892 |  |
| CZF1 | MKS1 | AT2G40140.1 | AT3G18690.1 | Zinc finger CCCH domain-containing protein 29; Involved in salt stress response. May positively modulate plant tolerance to salt stress | protein MKS1; Regulator of plant defense response. May contribute to MPK4-regulated defense activation by coupling the kinase to specific WRKY transcription factors | | 0.416 |  |
| CZF1 | MPK3 | AT2G40140.1 | AT3G45640.1 | Zinc finger CCCH domain-containing protein 29; Involved in salt stress response. May positively modulate plant tolerance to salt stress | Mitogen-activated protein kinase 3; Involved in oxidative stress-mediated signaling cascade to oxidative (such as ozone). Involved in the innate immune MAP kinase signaling cascade (MEKK1, MKK4/MKK5 and MPK3/MPK6) downstream of bacterial flagellin receptor FLS2. May be involved in hypersensitive response (HR)-mediated signaling cascade. May phosphorylate regulators of WRKY transcription factors. Mediates the phosphorylation of VIP1 and subsequent stress genes transcription in response to Agrobacterium | | 0.837 |  |
| CZF1 | STZ | AT2G40140.1 | AT1G27730.1 | Zinc finger CCCH domain-containing protein 29; Involved in salt stress response. May positively modulate plant tolerance to salt stress | Salt tolerance zinc finger; Transcriptional repressor involved in abiotic stress responses. Can repress the stress responsive genes DREB1A and LTI78. Probably involved in jasmonate (JA) early signaling response. May regulate the expression of the JA biosynthesis gene LOX3 and control the expression of TIFY10A/JAZ1, a key repressor in the JA signaling cascade | | 0.958 |  |
| CZF1 | WRKY33 | AT2G40140.1 | AT2G38470.1 | zinc finger CCCH domain-containing protein 29; Involved in salt stress response. May positively modulate plant tolerance to salt stress | WRKY DNA-binding protein 33; Transcription factor. Interacts specifically with the W box (5’-(T)TGAC[CT]-3’), a frequently occurring elicitor- responsive cis-acting element (By similarity) | | 0.967 |  |
| CZF1 | WRKY40 | AT2G40140.1 | AT1G80840.1 | Zinc finger CCCH domain-containing protein 29; Involved in salt stress response. May positively modulate plant tolerance to salt stress | WRKY DNA-binding protein 40; Transcription factor. Interacts specifically with the W box (5’-(T)TGAC[CT]-3’), a frequently occurring elicitor- responsive cis-acting element (By similarity) | | 0.957 |  |
| ERF104 | CNI1 | AT5G61600.1 | AT5G27420.1 | Ethylene response factor 104; Probably acts as a transcriptional activator. Binds to the GCC-box pathogenesis-related promoter element. May be involved in the regulation of gene expression by stress factors and by components of stress signal transduction pathways (By similarity) | Carbon/nitrogen insensitive 1; E3 ubiquitin-protein ligase that is required for the plant C/N response during seedling growth transition. May be involved in the early steps of the plant defense signaling pathway | | 0.614 |  |
| ERF104 | CZF1 | AT5G61600.1 | AT2G40140.1 | ethylene response factor 104; Probably acts as a transcriptional activator. Binds to the GCC-box pathogenesis-related promoter element. May be involved in the regulation of gene expression by stress factors and by components of stress signal transduction pathways (By similarity) | Zinc finger CCCH domain-containing protein 29; Involved in salt stress response. May positively modulate plant tolerance to salt stress | | 0.892 |  |
| ERF104 | MPK3 | AT5G61600.1 | AT3G45640.1 | Ethylene response factor 104; Probably acts as a transcriptional activator. Binds to the GCC-box pathogenesis-related promoter element. May be involved in the regulation of gene expression by stress factors and by components of stress signal transduction pathways (By similarity) | Mitogen-activated protein kinase 3; Involved in oxidative stress-mediated signaling cascade to oxidative (such as ozone). Involved in the innate immune MAP kinase signaling cascade (MEKK1, MKK4/MKK5 and MPK3/MPK6) downstream of bacterial flagellin receptor FLS2. May be involved in hypersensitive response (HR)-mediated signaling cascade. May phosphorylate regulators of WRKY transcription factors. Mediates the phosphorylation of VIP1 and subsequent stress genes transcription in response to Agrobacterium | | 0.683 |  |
| ERF104 | MPK4 | AT5G61600.1 | AT4G01370.1 | Ethylene response factor 104; Probably acts as a transcriptional activator. Binds to the GCC-box pathogenesis-related promoter element. May be involved in the regulation of gene expression by stress factors and by components of stress signal transduction pathways (By similarity) | MAP kinase 4; Involved in cortical microtubules organization and stabilization by regulating the phosphorylation state of microtubule-associated proteins such as MAP65-1. Involved in root hair development process. Negative regulator of systemic acquired resistance (SAR) and salicylic acid- (SA) mediated defense response. Required for Jasmonic acid- (JA) mediated defense gene expression. May regulate activity of transcription factor controlling pathogenesis-related (PR) gene expression. Seems to act independently of the SAR regulatory protein NPR1 (Nonexpresser of PR1). Phosphorylates M [...] | | 0.414 |  |
| ERF104 | STZ | AT5G61600.1 | AT1G27730.1 | Ethylene response factor 104; Probably acts as a transcriptional activator. Binds to the GCC-box pathogenesis-related promoter element. May be involved in the regulation of gene expression by stress factors and by components of stress signal transduction pathways (By similarity) | salt tolerance zinc finger; Transcriptional repressor involved in abiotic stress responses. Can repress the stress responsive genes DREB1A and LTI78. Probably involved in jasmonate (JA) early signaling response. May regulate the expression of the JA biosynthesis gene LOX3 and control the expression of TIFY10A/JAZ1, a key repressor in the JA signaling cascade | | 0.946 |  |
| ERF104 | WRKY33 | AT5G61600.1 | AT2G38470.1 | ethylene response factor 104; Probably acts as a transcriptional activator. Binds to the GCC-box pathogenesis-related promoter element. May be involved in the regulation of gene expression by stress factors and by components of stress signal transduction pathways (By similarity) | WRKY DNA-binding protein 33; Transcription factor. Interacts specifically with the W box (5’-(T)TGAC[CT]-3’), a frequently occurring elicitor- responsive cis-acting element (By similarity) | | 0.956 |  |
| ERF104 | | WRKY40 | AT5G61600.1 | AT1G80840.1 | ethylene response factor 104; Probably acts as a transcriptional activator. Binds to the GCC-box pathogenesis-related promoter element. May be involved in the regulation of gene expression by stress factors and by components of stress signal transduction pathways (By similarity) | WRKY DNA-binding protein 40; Transcription factor. Interacts specifically with the W box (5’-(T)TGAC[CT]-3’), a frequently occurring elicitor- responsive cis-acting element (By similarity) | 0.762 | |
| MKS1 | | CNI1 | AT3G18690.1 | AT5G27420.1 | protein MKS1; Regulator of plant defense response. May contribute to MPK4-regulated defense activation by coupling the kinase to specific WRKY transcription factors | Carbon/nitrogen insensitive 1; E3 ubiquitin-protein ligase that is required for the plant C/N response during seedling growth transition. May be involved in the early steps of the plant defense signaling pathway | 0.560 | |
| MKS1 | | CZF1 | AT3G18690.1 | AT2G40140.1 | protein MKS1; Regulator of plant defense response. May contribute to MPK4-regulated defense activation by coupling the kinase to specific WRKY transcription factors | Zinc finger CCCH domain-containing protein 29; Involved in salt stress response. May positively modulate plant tolerance to salt stress | 0.416 | |
| MKS1 | | MPK3 | AT3G18690.1 | AT3G45640.1 | protein MKS1; Regulator of plant defense response. May contribute to MPK4-regulated defense activation by coupling the kinase to specific WRKY transcription factors | Mitogen-activated protein kinase 3; Involved in oxidative stress-mediated signaling cascade to oxidative (such as ozone). Involved in the innate immune MAP kinase signaling cascade (MEKK1, MKK4/MKK5 and MPK3/MPK6) downstream of bacterial flagellin receptor FLS2. May be involved in hypersensitive response (HR)-mediated signaling cascade. May phosphorylate regulators of WRKY transcription factors. Mediates the phosphorylation of VIP1 and subsequent stress genes transcription in response to Agrobacterium | 0.614 | |
| MKS1 | | MPK4 | AT3G18690.1 | AT4G01370.1 | protein MKS1; Regulator of plant defense response. May contribute to MPK4-regulated defense activation by coupling the kinase to specific WRKY transcription factors | MAP kinase 4; Involved in cortical microtubules organization and stabilization by regulating the phosphorylation state of microtubule-associated proteins such as MAP65-1. Involved in root hair development process. Negative regulator of systemic acquired resistance (SAR) and salicylic acid- (SA) mediated defense response. Required for Jasmonic acid- (JA) mediated defense gene expression. May regulate activity of transcription factor controlling pathogenesis-related (PR) gene expression. Seems to act independently of the SAR regulatory protein NPR1 (Nonexpresser of PR1). Phosphorylates M [...] | 0.999 | |
| MKS1 | | SIB1 | AT3G18690.1 | AT3G56710.1 | Protein MKS1; Regulator of plant defense response. May contribute to MPK4-regulated defense activation by coupling the kinase to specific WRKY transcription factors | sigma factor binding protein 1; Contributes to plant defense. May regulate chloroplast metabolism upon infection with necrotrophic pathogens such as Pseudomonas syringae | 0.765 | |
| MKS1 | | SIB2 | AT3G18690.1 | AT2G41180.1 | Protein MKS1; Regulator of plant defense response. May contribute to MPK4-regulated defense activation by coupling the kinase to specific WRKY transcription factors | VQ motif-containing protein | 0.457 | |
| MKS1 | | WRKY33 | AT3G18690.1 | AT2G38470.1 | Protein MKS1; Regulator of plant defense response. May contribute to MPK4-regulated defense activation by coupling the kinase to specific WRKY transcription factors | WRKY DNA-binding protein 33; Transcription factor. Interacts specifically with the W box (5’-(T)TGAC[CT]-3’), a frequently occurring elicitor- responsive cis-acting element (By similarity) | 0.997 | |
| MPK3 | | CNI1 | AT3G45640.1 | AT5G27420.1 | Mitogen-activated protein kinase 3; Involved in oxidative stress-mediated signaling cascade to oxidative (such as ozone). Involved in the innate immune MAP kinase signaling cascade (MEKK1, MKK4/MKK5 and MPK3/MPK6) downstream of bacterial flagellin receptor FLS2. May be involved in hypersensitive response (HR)-mediated signaling cascade. May phosphorylate regulators of WRKY transcription factors. Mediates the phosphorylation of VIP1 and subsequent stress genes transcription in response to Agrobacterium | Carbon/nitrogen insensitive 1; E3 ubiquitin-protein ligase that is required for the plant C/N response during seedling growth transition. May be involved in the early steps of the plant defense signaling pathway | 0.698 | |
| MPK3 | | CZF1 | AT3G45640.1 | AT2G40140.1 | Mitogen-activated protein kinase 3; Involved in oxidative stress-mediated signaling cascade to oxidative (such as ozone). Involved in the innate immune MAP kinase signaling cascade (MEKK1, MKK4/MKK5 and MPK3/MPK6) downstream of bacterial flagellin receptor FLS2. May be involved in hypersensitive response (HR)-mediated signaling cascade. May phosphorylate regulators of WRKY transcription factors. Mediates the phosphorylation of VIP1 and subsequent stress genes transcription in response to Agrobacterium | zinc finger CCCH domain-containing protein 29; Involved in salt stress response. May positively modulate plant tolerance to salt stress | 0.837 | |
| MPK3 | | ERF104 | AT3G45640.1 | AT5G61600.1 | Mitogen-activated protein kinase 3; Involved in oxidative stress-mediated signaling cascade to oxidative (such as ozone). Involved in the innate immune MAP kinase signaling cascade (MEKK1, MKK4/MKK5 and MPK3/MPK6) downstream of bacterial flagellin receptor FLS2. May be involved in hypersensitive response (HR)-mediated signaling cascade. May phosphorylate regulators of WRKY transcription factors. Mediates the phosphorylation of VIP1 and subsequent stress genes transcription in response to Agrobacterium | Ethylene response factor 104; Probably acts as a transcriptional activator. Binds to the GCC-box pathogenesis-related promoter element. May be involved in the regulation of gene expression by stress factors and by components of stress signal transduction pathways (By similarity) | 0.683 | |
| MPK3 | | MKS1 | AT3G45640.1 | AT3G18690.1 | Mitogen-activated protein kinase 3; Involved in oxidative stress-mediated signaling cascade to oxidative (such as ozone). Involved in the innate immune MAP kinase signaling cascade (MEKK1, MKK4/MKK5 and MPK3/MPK6) downstream of bacterial flagellin receptor FLS2. May be involved in hypersensitive response (HR)-mediated signaling cascade. May phosphorylate regulators of WRKY transcription factors. Mediates the phosphorylation of VIP1 and subsequent stress genes transcription in response to Agrobacterium | protein MKS1; Regulator of plant defense response. May contribute to MPK4-regulated defense activation by coupling the kinase to specific WRKY transcription factors | 0.614 | |
| MPK3 | | MPK4 | AT3G45640.1 | AT4G01370.1 | Mitogen-activated protein kinase 3; Involved in oxidative stress-mediated signaling cascade to oxidative (such as ozone). Involved in the innate immune MAP kinase signaling cascade (MEKK1, MKK4/MKK5 and MPK3/MPK6) downstream of bacterial flagellin receptor FLS2. May be involved in hypersensitive response (HR)-mediated signaling cascade. May phosphorylate regulators of WRKY transcription factors. Mediates the phosphorylation of VIP1 and subsequent stress genes transcription in response to Agrobacterium | MAP kinase 4; Involved in cortical microtubules organization and stabilization by regulating the phosphorylation state of microtubule-associated proteins such as MAP65-1. Involved in root hair development process. Negative regulator of systemic acquired resistance (SAR) and salicylic acid- (SA) mediated defense response. Required for jasmonic acid- (JA) mediated defense gene expression. May regulate activity of transcription factor controlling pathogenesis-related (PR) gene expression. Seems to act independently of the SAR regulatory protein NPR1 (Nonexpresser of PR1). Phosphorylates M [...] | 0.903 | |
| MPK3 | | SIB1 | AT3G45640.1 | AT3G56710.1 | Mitogen-activated protein kinase 3; Involved in oxidative stress-mediated signaling cascade to oxidative (such as ozone). Involved in the innate immune MAP kinase signaling cascade (MEKK1, MKK4/MKK5 and MPK3/MPK6) downstream of bacterial flagellin receptor FLS2. May be involved in hypersensitive response (HR)-mediated signaling cascade. May phosphorylate regulators of WRKY transcription factors. Mediates the phosphorylation of VIP1 and subsequent stress genes transcription in response to Agrobacterium | sigma factor binding protein 1; Contributes to plant defense. May regulate chloroplast metabolism upon infection with necrotrophic pathogens such as Pseudomonas syringae | 0.402 | |
| MPK3 | | STZ | AT3G45640.1 | AT1G27730.1 | Mitogen-activated protein kinase 3; Involved in oxidative stress-mediated signaling cascade to oxidative (such as ozone). Involved in the innate immune MAP kinase signaling cascade (MEKK1, MKK4/MKK5 and MPK3/MPK6) downstream of bacterial flagellin receptor FLS2. May be involved in hypersensitive response (HR)-mediated signaling cascade. May phosphorylate regulators of WRKY transcription factors. Mediates the phosphorylation of VIP1 and subsequent stress genes transcription in response to Agrobacterium | salt tolerance zinc finger; Transcriptional repressor involved in abiotic stress responses. Can repress the stress responsive genes DREB1A and LTI78. Probably involved in jasmonate (JA) early signaling response. May regulate the expression of the JA biosynthesis gene LOX3 and control the expression of TIFY10A/JAZ1, a key repressor in the JA signaling cascade | 0.958 | |
| MPK3 | | WRKY33 | AT3G45640.1 | AT2G38470.1 | Mitogen-activated protein kinase 3; Involved in oxidative stress-mediated signaling cascade to oxidative (such as ozone). Involved in the innate immune MAP kinase signaling cascade (MEKK1, MKK4/MKK5 and MPK3/MPK6) downstream of bacterial flagellin receptor FLS2. May be involved in hypersensitive response (HR)-mediated signaling cascade. May phosphorylate regulators of WRKY transcription factors. Mediates the phosphorylation of VIP1 and subsequent stress genes transcription in response to Agrobacterium | WRKY DNA-binding protein 33; Transcription factor. Interacts specifically with the W box (5’-(T)TGAC[CT]-3’), a frequently occurring elicitor- responsive cis-acting element (By similarity) | 0.996 | |
| MPK3 | | WRKY40 | AT3G45640.1 | AT1G80840.1 | Mitogen-activated protein kinase 3; Involved in oxidative stress-mediated signaling cascade to oxidative (such as ozone). Involved in the innate immune MAP kinase signaling cascade (MEKK1, MKK4/MKK5 and MPK3/MPK6) downstream of bacterial flagellin receptor FLS2. May be involved in hypersensitive response (HR)-mediated signaling cascade. May phosphorylate regulators of WRKY transcription factors. Mediates the phosphorylation of VIP1 and subsequent stress genes transcription in response to Agrobacterium | WRKY DNA-binding protein 40; Transcription factor. Interacts specifically with the W box (5’-(T)TGAC[CT]-3’), a frequently occurring elicitor- responsive cis-acting element (By similarity) | 0.745 | |
| MPK4 | | ERF104 | AT4G01370.1 | AT5G61600.1 | MAP kinase 4; Involved in cortical microtubules organization and stabilization by regulating the phosphorylation state of microtubule-associated proteins such as MAP65-1. Involved in root hair development process. Negative regulator of systemic acquired resistance (SAR) and salicylic acid- (SA) mediated defense response. Required for jasmonic acid- (JA) mediated defense gene expression. May regulate activity of transcription factor controlling pathogenesis-related (PR) gene expression. Seems to act independently of the SAR regulatory protein NPR1 (None presser of PR1). Phosphorylates M [...] | Ethylene response factor 104; Probably acts as a transcriptional activator. Binds to the GCC-box pathogenesis-related promoter element. May be involved in the regulation of gene expression by stress factors and by components of stress signal transduction pathways (By similarity) | 0.414 | |
| MPK4 | | MKS1 | AT4G01370.1 | AT3G18690.1 | MAP kinase 4; Involved in cortical microtubules organization and stabilization by regulating the phosphorylation state of microtubule-associated proteins such as MAP65-1. Involved in root hair development process. Negative regulator of systemic acquired resistance (SAR) and salicylic acid- (SA) mediated defense response. Required for jasmonic acid- (JA) mediated defense gene expression. May regulate activity of transcription factor controlling pathogenesis-related (PR) gene expression. Seems to act independently of the SAR regulatory protein NPR1 (Nonexpresser of PR1). Phosphorylates M [...] | Protein MKS1; Regulator of plant defense response. May contribute to MPK4-regulated defense activation by coupling the kinase to specific WRKY transcription factors | 0.999 | |
| MPK4 | | MPK3 | AT4G01370.1 | AT3G45640.1 | MAP kinase 4; Involved in cortical microtubules organization and stabilization by regulating the phosphorylation state of microtubule-associated proteins such as MAP65-1. Involved in root hair development process. Negative regulator of systemic acquired resistance (SAR) and salicylic acid- (SA) mediated defense response. Required for Jasmonic acid- (JA) mediated defense gene expression. May regulate activity of transcription factor controlling pathogenesis-related (PR) gene expression. Seems to act independently of the SAR regulatory protein NPR1 (Nonexpresser of PR1). Phosphorylates M [...] | Mitogen-activated protein kinase 3; Involved in oxidative stress-mediated signaling cascade to oxidative (such as ozone). Involved in the innate immune MAP kinase signaling cascade (MEKK1, MKK4/MKK5 and MPK3/MPK6) downstream of bacterial flagellin receptor FLS2. May be involved in hypersensitive response (HR)-mediated signaling cascade. May phosphorylate regulators of WRKY transcription factors. Mediates the phosphorylation of VIP1 and subsequent stress genes transcription in response to Agrobacterium | 0.903 | |

| MPK4 | | WRKY33 | | AT4G01370.1 | | AT2G38470.1 | | MAP kinase 4; Involved in cortical microtubules organization and stabilization by regulating the phosphorylation state of microtubule-associated proteins such as MAP65-1. Involved in root hair development process. Negative regulator of systemic acquired resistance (SAR) and salicylic acid- (SA) mediated defense response. Required for jasmonic acid- (JA) mediated defense gene expression. May regulate activity of transcription factor controlling pathogenesis-related (PR) gene expression. Seems to act independently of the SAR regulatory protein NPR1 (Nonexpresser of PR1). Phosphorylates M [...] | | WRKY DNA-binding protein 33; Transcription factor. Interacts specifically with the W box (5’-(T)TGAC[CT]-3’), a frequently occurring elicitor- responsive cis-acting element (By similarity) | | 0.992 |
| --- | --- | --- | --- | --- | --- | --- | --- | --- | --- | --- | --- | --- |
| SIB1 | | MKS1 | | AT3G56710.1 | | AT3G18690.1 | | sigma factor binding protein 1; Contributes to plant defense. May regulate chloroplast metabolism upon infection with necrotrophic pathogens such as Pseudomonas syringae | | Protein MKS1; Regulator of plant defense response. May contribute to MPK4-regulated defense activation by coupling the kinase to specific WRKY transcription factors | | 0.765 |
| SIB1 | | MPK3 | | AT3G56710.1 | | AT3G45640.1 | | Sigma factor binding protein 1; Contributes to plant defense. May regulate chloroplast metabolism upon infection with necrotrophic pathogens such as Pseudomonas syringae | | Mitogen-activated protein kinase 3; Involved in oxidative stress-mediated signaling cascade to oxidative (such as ozone). Involved in the innate immune MAP kinase signaling cascade (MEKK1, MKK4/MKK5 and MPK3/MPK6) downstream of bacterial flagellin receptor FLS2. May be involved in hypersensitive response (HR)-mediated signaling cascade. May phosphorylate regulators of WRKY transcription factors. Mediates the phosphorylation of VIP1 and subsequent stress genes transcription in response to Agrobacterium | | 0.402 |
| SIB1 | | WRKY33 | | AT3G56710.1 | | AT2G38470.1 | | Sigma factor binding protein 1; Contributes to plant defense. May regulate chloroplast metabolism upon infection with necrotrophic pathogens such as Pseudomonas syringae | | WRKY DNA-binding protein 33; Transcription factor. Interacts specifically with the W box (5’-(T)TGAC[CT]-3’), a frequently occurring elicitor- responsive cis-acting element (By similarity) | | 0.989 |
| SIB1 | | WRKY40 | | AT3G56710.1 | | AT1G80840.1 | | Sigma factor binding protein 1; Contributes to plant defense. May regulate chloroplast metabolism upon infection with necrotrophic pathogens such as Pseudomonas syringae | | WRKY DNA-binding protein 40; Transcription factor. Interacts specifically with the W box (5’-(T)TGAC[CT]-3’), a frequently occurring elicitor- responsive cis-acting element (By similarity) | | 0.436 |
| SIB2 | | MKS1 | | AT2G41180.1 | | AT3G18690.1 | | VQ motif-containing protein | | protein MKS1; Regulator of plant defense response. May contribute to MPK4-regulated defense activation by coupling the kinase to specific WRKY transcription factors | | 0.457 |
| SIB2 | | WRKY33 | | AT2G41180.1 | | AT2G38470.1 | | VQ motif-containing protein | | WRKY DNA-binding protein 33; Transcription factor. Interacts specifically with the W box (5’-(T)TGAC[CT]-3’), a frequently occurring elicitor- responsive cis-acting element (By similarity) | | 0.988 |
| STZ | | CNI1 | | AT1G27730.1 | | AT5G27420.1 | | salt tolerance zinc finger; Transcriptional repressor involved in abiotic stress responses. Can repress the stress responsive genes DREB1A and LTI78. Probably involved in jasmonate (JA) early signaling response. May regulate the expression of the JA biosynthesis gene LOX3 and control the expression of TIFY10A/JAZ1, a key repressor in the JA signaling cascade | | Carbon/nitrogen insensitive 1; E3 ubiquitin-protein ligase that is required for the plant C/N response during seedling growth transition. May be involved in the early steps of the plant defense signaling pathway | | 0.896 |
| STZ | | CZF1 | | AT1G27730.1 | | AT2G40140.1 | | salt tolerance zinc finger; Transcriptional repressor involved in abiotic stress responses. Can repress the stress responsive genes DREB1A and LTI78. Probably involved in jasmonate (JA) early signaling response. May regulate the expression of the JA biosynthesis gene LOX3 and control the expression of TIFY10A/JAZ1, a key repressor in the JA signaling cascade | | zinc finger CCCH domain-containing protein 29; Involved in salt stress response. May positively modulate plant tolerance to salt stress | | 0.958 |
| STZ | | ERF104 | | AT1G27730.1 | | AT5G61600.1 | | Salt tolerance zinc finger; Transcriptional repressor involved in abiotic stress responses. Can repress the stress responsive genes DREB1A and LTI78. Probably involved in jasmonate (JA) early signaling response. May regulate the expression of the JA biosynthesis gene LOX3 and control the expression of TIFY10A/JAZ1, a key repressor in the JA signaling cascade | | Ethylene response factor 104; Probably acts as a transcriptional activator. Binds to the GCC-box pathogenesis-related promoter element. May be involved in the regulation of gene expression by stress factors and by components of stress signal transduction pathways (By similarity) | | 0.946 |
| STZ | | MPK3 | | AT1G27730.1 | | AT3G45640.1 | | salt tolerance zinc finger; Transcriptional repressor involved in abiotic stress responses. Can repress the stress responsive genes DREB1A and LTI78. Probably involved in jasmonate (JA) early signaling response. May regulate the expression of the JA biosynthesis gene LOX3 and control the expression of TIFY10A/JAZ1, a key repressor in the JA signaling cascade | | Mitogen-activated protein kinase 3; Involved in oxidative stress-mediated signaling cascade to oxidative (such as ozone). Involved in the innate immune MAP kinase signaling cascade (MEKK1, MKK4/MKK5 and MPK3/MPK6) downstream of bacterial flagellin receptor FLS2. May be involved in hypersensitive response (HR)-mediated signaling cascade. May phosphorylate regulators of WRKY transcription factors. Mediates the phosphorylation of VIP1 and subsequent stress genes transcription in response to Agrobacterium | | 0.958 |
| STZ | | WRKY33 | | AT1G27730.1 | | AT2G38470.1 | | salt tolerance zinc finger; Transcriptional repressor involved in abiotic stress responses. Can repress the stress responsive genes DREB1A and LTI78. Probably involved in jasmonate (JA) early signaling response. May regulate the expression of the JA biosynthesis gene LOX3 and control the expression of TIFY10A/JAZ1, a key repressor in the JA signaling cascade | | WRKY DNA-binding protein 33; Transcription factor. Interacts specifically with the W box (5’-(T)TGAC[CT]-3’), a frequently occurring elicitor- responsive cis-acting element (By similarity) | | 0.958 |
| STZ | | WRKY40 | | AT1G27730.1 | | AT1G80840.1 | | salt tolerance zinc finger; Transcriptional repressor involved in abiotic stress responses. Can repress the stress responsive genes DREB1A and LTI78. Probably involved in jasmonate (JA) early signaling response. May regulate the expression of the JA biosynthesis gene LOX3 and control the expression of TIFY10A/JAZ1, a key repressor in the JA signaling cascade | | WRKY DNA-binding protein 40; Transcription factor. Interacts specifically with the W box (5’-(T)TGAC[CT]-3’), a frequently occurring elicitor- responsive cis-acting element (By similarity) | | 0.974 |
| WRKY33 | | CNI1 | | AT2G38470.1 | | AT5G27420.1 | | WRKY DNA-binding protein 33; Transcription factor. Interacts specifically with the W box (5’-(T)TGAC[CT]-3’), a frequently occurring elicitor- responsive cis-acting element (By similarity) | | Carbon/nitrogen insensitive 1; E3 ubiquitin-protein ligase that is required for the plant C/N response during seedling growth transition. May be involved in the early steps of the plant defense signaling pathway | | 0.954 |
| WRKY33 | | CZF1 | | AT2G38470.1 | | AT2G40140.1 | | WRKY DNA-binding protein 33; Transcription factor. Interacts specifically with the W box (5’-(T)TGAC[CT]-3’), a frequently occurring elicitor- responsive cis-acting element (By similarity) | | zinc finger CCCH domain-containing protein 29; Involved in salt stress response. May positively modulate plant tolerance to salt stress | | 0.967 |
| WRKY33 | | ERF104 | | AT2G38470.1 | | AT5G61600.1 | | WRKY DNA-binding protein 33; Transcription factor. Interacts specifically with the W box (5’-(T)TGAC[CT]-3’), a frequently occurring elicitor- responsive cis-acting element (By similarity) | | Ethylene response factor 104; Probably acts as a transcriptional activator. Binds to the GCC-box pathogenesis-related promoter element. May be involved in the regulation of gene expression by stress factors and by components of stress signal transduction pathways (By similarity) | | 0.956 |
| WRKY33 | | MKS1 | | AT2G38470.1 | | AT3G18690.1 | | WRKY DNA-binding protein 33; Transcription factor. Interacts specifically with the W box (5’-(T)TGAC[CT]-3’), a frequently occurring elicitor- responsive cis-acting element (By similarity) | | Protein MKS1; Regulator of plant defense response. May contribute to MPK4-regulated defense activation by coupling the kinase to specific WRKY transcription factors | | 0.997 |
| WRKY33 | | MPK3 | | AT2G38470.1 | | AT3G45640.1 | | WRKY DNA-binding protein 33; Transcription factor. Interacts specifically with the W box (5’-(T)TGAC[CT]-3’), a frequently occurring elicitor- responsive cis-acting element (By similarity) | | Mitogen-activated protein kinase 3; Involved in oxidative stress-mediated signaling cascade to oxidative (such as ozone). Involved in the innate immune MAP kinase signaling cascade (MEKK1, MKK4/MKK5 and MPK3/MPK6) downstream of bacterial flagellin receptor FLS2. May be involved in hypersensitive response (HR)-mediated signaling cascade. May phosphorylate regulators of WRKY transcription factors. Mediates the phosphorylation of VIP1 and subsequent stress genes transcription in response to Agrobacterium | | 0.996 |
| WRKY33 | | MPK4 | | AT2G38470.1 | | AT4G01370.1 | | WRKY DNA-binding protein 33; Transcription factor. Interacts specifically with the W box (5’-(T)TGAC[CT]-3’), a frequently occurring elicitor- responsive cis-acting element (By similarity) | | MAP kinase 4; Involved in cortical microtubules organization and stabilization by regulating the phosphorylation state of microtubule-associated proteins such as MAP65-1. Involved in root hair development process. Negative regulator of systemic acquired resistance (SAR) and salicylic acid- (SA) mediated defense response. Required for Jasmonic acid- (JA) mediated defense gene expression. May regulate activity of transcription factor controlling pathogenesis-related (PR) gene expression. Seems to act independently of the SAR regulatory protein NPR1 (Nonexpresser of PR1). Phosphorylates M [...] | | 0.992 |
| WRKY33 | | SIB1 | | AT2G38470.1 | | AT3G56710.1 | | WRKY DNA-binding protein 33; Transcription factor. Interacts specifically with the W box (5’-(T)TGAC[CT]-3’), a frequently occurring elicitor- responsive cis-acting element (By similarity) | | sigma factor binding protein 1; Contributes to plant defense. May regulate chloroplast metabolism upon infection with necrotrophic pathogens such as Pseudomonas syringae | | 0.989 |
| WRKY33 | SIB2 | AT2G38470.1 | | AT2G41180.1 | | WRKY DNA-binding protein 33; Transcription factor. Interacts specifically with the W box (5’-(T)TGAC[CT]-3’), a frequently occurring elicitor- responsive cis-acting element (By similarity) | | VQ motif-containing protein | | 0.988 | |  |
| WRKY33 | STZ | AT2G38470.1 | | AT1G27730.1 | | WRKY DNA-binding protein 33; Transcription factor. Interacts specifically with the W box (5’-(T)TGAC[CT]-3’), a frequently occurring elicitor- responsive cis-acting element (By similarity) | | salt tolerance zinc finger; Transcriptional repressor involved in abiotic stress responses. Can repress the stress responsive genes DREB1A and LTI78. Probably involved in jasmonate (JA) early signaling response. May regulate the expression of the JA biosynthesis gene LOX3 and control the expression of TIFY10A/JAZ1, a key repressor in the JA signaling cascade | | 0.958 | |  |
| WRKY33 | WRKY40 | AT2G38470.1 | | AT1G80840.1 | | WRKY DNA-binding protein 33; Transcription factor. Interacts specifically with the W box (5’-(T)TGAC[CT]-3’), a frequently occurring elicitor- responsive cis-acting element (By similarity) | | WRKY DNA-binding protein 40; Transcription factor. Interacts specifically with the W box (5’-(T)TGAC[CT]-3’), a frequently occurring elicitor- responsive cis-acting element (By similarity) | | 0.974 | |  |
| WRKY40 | CNI1 | AT1G80840.1 | | AT5G27420.1 | | WRKY DNA-binding protein 40; Transcription factor. Interacts specifically with the W box (5’-(T)TGAC[CT]-3’), a frequently occurring elicitor- responsive cis-acting element (By similarity) | | carbon/nitrogen insensitive 1; E3 ubiquitin-protein ligase that is required for the plant C/N response during seedling growth transition. May be involved in the early steps of the plant defense signaling pathway | | 0.839 | |  |
| WRKY40 | CZF1 | AT1G80840.1 | | AT2G40140.1 | | WRKY DNA-binding protein 40; Transcription factor. Interacts specifically with the W box (5’-(T)TGAC[CT]-3’), a frequently occurring elicitor- responsive cis-acting element (By similarity) | | zinc finger CCCH domain-containing protein 29; Involved in salt stress response. May positively modulate plant tolerance to salt stress | | 0.957 | |  |
| WRKY40 | ERF104 | AT1G80840.1 | | AT5G61600.1 | | WRKY DNA-binding protein 40; Transcription factor. Interacts specifically with the W box (5’-(T)TGAC[CT]-3’), a frequently occurring elicitor- responsive cis-acting element (By similarity) | | ethylene response factor 104; Probably acts as a transcriptional activator. Binds to the GCC-box pathogenesis-related promoter element. May be involved in the regulation of gene expression by stress factors and by components of stress signal transduction pathways (By similarity) | | 0.762 | |  |
| WRKY40 | MPK3 | AT1G80840.1 | | AT3G45640.1 | | WRKY DNA-binding protein 40; Transcription factor. Interacts specifically with the W box (5’-(T)TGAC[CT]-3’), a frequently occurring elicitor- responsive cis-acting element (By similarity) | | mitogen-activated protein kinase 3; Involved in oxidative stress-mediated signaling cascade to oxidative (such as ozone). Involved in the innate immune MAP kinase signaling cascade (MEKK1, MKK4/MKK5 and MPK3/MPK6) downstream of bacterial flagellin receptor FLS2. May be involved in hypersensitive response (HR)-mediated signaling cascade. May phosphorylate regulators of WRKY transcription factors. Mediates the phosphorylation of VIP1 and subsequent stress genes transcription in response to Agrobacterium | | 0.745 | |  |
| WRKY40 | SIB1 | AT1G80840.1 | | AT3G56710.1 | | WRKY DNA-binding protein 40; Transcription factor. Interacts specifically with the W box (5’-(T)TGAC[CT]-3’), a frequently occurring elicitor- responsive cis-acting element (By similarity) | | sigma factor binding protein 1; Contributes to plant defense. May regulate chloroplast metabolism upon infection with necrotrophic pathogens such as Pseudomonas syringae | | 0.436 | |  |
| WRKY40 | STZ | AT1G80840.1 | | AT1G27730.1 | | WRKY DNA-binding protein 40; Transcription factor. Interacts specifically with the W box (5’-(T)TGAC[CT]-3’), a frequently occurring elicitor- responsive cis-acting element (By similarity) | | salt tolerance zinc finger; Transcriptional repressor involved in abiotic stress responses. Can repress the stress responsive genes DREB1A and LTI78. Probably involved in jasmonate (JA) early signaling response. May regulate the expression of the JA biosynthesis gene LOX3 and control the expression of TIFY10A/JAZ1, a key repressor in the JA signaling cascade | | 0.974 | |  |
| WRKY40 | WRKY33 | AT1G80840.1 | | AT2G38470.1 | | WRKY DNA-binding protein 40; Transcription factor. Interacts specifically with the W box (5’-(T)TGAC[CT]-3’), a frequently occurring elicitor- responsive cis-acting element (By similarity) | | WRKY DNA-binding protein 33; Transcription factor. Interacts specifically with the W box (5’-(T)TGAC[CT]-3’), a frequently occurring elicitor- responsive cis-acting element (By similarity) | | 0.974 | |  |

Table: Protein-Protein functional interactive association: List of all the possible interactors from Node 1 and Node2 with their functional annotation, interacting score values and accession number in tomato (*Solanum lycopersicum*)

| CZFP1 | MPK3 | Solyc04g077980.1.1 | Solyc06g005170.2.1 | C2H2-type zinc finger protein; Cold zinc finger protein 1 | Mitogen-activated protein kinase 3 | 0.803 |
| --- | --- | --- | --- | --- | --- | --- |
| CZFP1 | Solyc03g116890.2.1 | Solyc04g077980.1.1 | Solyc03g116890.2.1 | C2H2-type zinc finger protein; Cold zinc finger protein 1 | WRKY | 0.679 |
| CZFP1 | Solyc06g066370.2.1 | Solyc04g077980.1.1 | Solyc06g066370.2.1 | C2H2-type zinc finger protein; Cold zinc finger protein 1 | probable WRKY transcription factor 33-like | 0.558 |
| CZFP1 | Solyc06g068460.2.1 | Solyc04g077980.1.1 | Solyc06g068460.2.1 | C2H2-type zinc finger protein; Cold zinc finger protein 1 | WRKY transcription factor 1 | 0.679 |
| CZFP1 | Solyc08g067340.2.1 | Solyc04g077980.1.1 | Solyc08g067340.2.1 | C2H2-type zinc finger protein; Cold zinc finger protein 1 | probable WRKY transcription factor 40-like | 0.679 |
| MAPK5 | Solyc03g095770.2.1 | Solyc01g094960.2.1 | Solyc03g095770.2.1 | Mitogen-activated protein kinase 5 | probable WRKY transcription factor 70-like | 0.527 |
| MAPK5 | Solyc06g066370.2.1 | Solyc01g094960.2.1 | Solyc06g066370.2.1 | Mitogen-activated protein kinase 5 | probable WRKY transcription factor 33-like | 0.953 |
| MPK3 | CZFP1 | Solyc06g005170.2.1 | Solyc04g077980.1.1 | Mitogen-activated protein kinase 3 | C2H2-type zinc finger protein; Cold zinc finger protein 1 | 0.803 |
| MPK3 | Solyc06g066370.2.1 | Solyc06g005170.2.1 | Solyc06g066370.2.1 | Mitogen-activated protein kinase 3 | probable WRKY transcription factor 33-like | 0.931 |
| Solyc01g096510.2.1 | Solyc06g066370.2.1 | Solyc01g096510.2.1 | Solyc06g066370.2.1 | sigma factor binding protein 1, chloroplastic-like | probable WRKY transcription factor 33-like | 0.955 |
| Solyc03g095770.2.1 | MAPK5 | Solyc03g095770.2.1 | Solyc01g094960.2.1 | probable WRKY transcription factor 70-like | Mitogen-activated protein kinase 5 | 0.527 |
| Solyc03g095770.2.1 | Solyc03g116890.2.1 | Solyc03g095770.2.1 | Solyc03g116890.2.1 | probable WRKY transcription factor 70-like | WRKY | 0.568 |
| Solyc03g095770.2.1 | Solyc06g066370.2.1 | Solyc03g095770.2.1 | Solyc06g066370.2.1 | probable WRKY transcription factor 70-like | probable WRKY transcription factor 33-like | 0.572 |
| Solyc03g095770.2.1 | Solyc06g068460.2.1 | Solyc03g095770.2.1 | Solyc06g068460.2.1 | probable WRKY transcription factor 70-like | WRKY transcription factor 1 | 0.568 |
| Solyc03g095770.2.1 | Solyc08g067340.2.1 | Solyc03g095770.2.1 | Solyc08g067340.2.1 | probable WRKY transcription factor 70-like | probable WRKY transcription factor 40-like | 0.568 |
| Solyc03g116890.2.1 | CZFP1 | Solyc03g116890.2.1 | Solyc04g077980.1.1 | WRKY | C2H2-type zinc finger protein; Cold zinc finger protein 1 | 0.679 |
| Solyc03g116890.2.1 | Solyc03g095770.2.1 | Solyc03g116890.2.1 | Solyc03g095770.2.1 | WRKY | probable WRKY transcription factor 70-like | 0.568 |
| Solyc03g116890.2.1 | Solyc06g066370.2.1 | Solyc03g116890.2.1 | Solyc06g066370.2.1 | WRKY | probable WRKY transcription factor 33-like | 0.771 |
| Solyc06g060470.1.1 | Solyc06g066370.2.1 | Solyc06g060470.1.1 | Solyc06g066370.2.1 | protein MKS1-like | probable WRKY transcription factor 33-like | 0.913 |
| Solyc06g066370.2.1 | CZFP1 | Solyc06g066370.2.1 | Solyc04g077980.1.1 | probable WRKY transcription factor 33-like | C2H2-type zinc finger protein; Cold zinc finger protein 1 | 0.558 |

| Solyc06g066370.2.1 | MAPK5 | Solyc06g066370.2.1 | Solyc01g094960.2.1 | probable WRKY transcription factor 33-like | Mitogen-activated protein kinase 5 | 0.953 |
| --- | --- | --- | --- | --- | --- | --- |
| Solyc06g066370.2.1 | MPK3 | Solyc06g066370.2.1 | Solyc06g005170.2.1 | probable WRKY transcription factor 33-like | Mitogen-activated protein kinase 3 | 0.931 |
| Solyc06g066370.2.1 | Solyc01g096510.2.1 | Solyc06g066370.2.1 | Solyc01g096510.2.1 | probable WRKY transcription factor 33-like | sigma factor binding protein 1, chloroplastic-like | 0.955 |
| Solyc06g066370.2.1 | Solyc03g095770.2.1 | Solyc06g066370.2.1 | Solyc03g095770.2.1 | probable WRKY transcription factor 33-like | probable WRKY transcription factor 70-like | 0.572 |
| Solyc06g066370.2.1 | Solyc03g116890.2.1 | Solyc06g066370.2.1 | Solyc03g116890.2.1 | probable WRKY transcription factor 33-like | WRKY | 0.771 |
| Solyc06g066370.2.1 | Solyc06g060470.1.1 | Solyc06g066370.2.1 | Solyc06g060470.1.1 | probable WRKY transcription factor 33-like | protein MKS1-like | 0.913 |
| Solyc06g066370.2.1 | Solyc06g068460.2.1 | Solyc06g066370.2.1 | Solyc06g068460.2.1 | probable WRKY transcription factor 33-like | WRKY transcription factor 1 | 0.772 |
| Solyc06g066370.2.1 | Solyc08g067340.2.1 | Solyc06g066370.2.1 | Solyc08g067340.2.1 | probable WRKY transcription factor 33-like | probable WRKY transcription factor 40-like | 0.762 |
| Solyc06g066370.2.1 | Solyc10g078440.1.1 | Solyc06g066370.2.1 | Solyc10g078440.1.1 | probable WRKY transcription factor 33-like | uncharacterized LOC101268236 | 0.809 |
| Solyc06g068460.2.1 | CZFP1 | Solyc06g068460.2.1 | Solyc04g077980.1.1 | WRKY transcription factor 1 | C2H2-type zinc finger protein; Cold zinc finger protein 1 | 0.679 |
| Solyc06g068460.2.1 | Solyc03g095770.2.1 | Solyc06g068460.2.1 | Solyc03g095770.2.1 | WRKY transcription factor 1 | probable WRKY transcription factor 70-like | 0.568 |
| Solyc06g068460.2.1 | Solyc06g066370.2.1 | Solyc06g068460.2.1 | Solyc06g066370.2.1 | WRKY transcription factor 1 | probable WRKY transcription factor 33-like | 0.772 |
| Solyc08g067340.2.1 | CZFP1 | Solyc08g067340.2.1 | Solyc04g077980.1.1 | probable WRKY transcription factor 40-like | C2H2-type zinc finger protein; Cold zinc finger protein 1 | 0.679 |
| Solyc08g067340.2.1 | Solyc03g095770.2.1 | Solyc08g067340.2.1 | Solyc03g095770.2.1 | probable WRKY transcription factor 40-like | probable WRKY transcription factor 70-like | 0.568 |
| Solyc08g067340.2.1 | Solyc06g066370.2.1 | Solyc08g067340.2.1 | Solyc06g066370.2.1 | probable WRKY transcription factor 40-like | probable WRKY transcription factor 33-like | 0.762 |
| Solyc10g078440.1.1 | Solyc06g066370.2.1 | Solyc10g078440.1.1 | Solyc06g066370.2.1 | uncharacterized LOC101268236 | probable WRKY transcription factor 33-like | 0.809 |
